# Understanding acute metabolic decompensation in propionic and methylmalonic acidemias: a deep metabolic phenotyping approach

**DOI:** 10.1186/s13023-020-1347-3

**Published:** 2020-03-06

**Authors:** H. A. Haijes, J. J. M. Jans, M. van der Ham, P. M. van Hasselt, N. M. Verhoeven-Duif

**Affiliations:** 1 Section Metabolic Diagnostics, Department of Genetics, Wilhelmina Children’s Hospital, University Medical Centre Utrecht, Utrecht University, Lundlaan 6, 3584 EA Utrecht, The Netherlands; 2 Section Metabolic Diseases, Department of Child Health, Wilhelmina Children’s Hospital, University Medical Centre Utrecht, Utrecht University, Lundlaan 6, 3584 EA Utrecht, The Netherlands

**Keywords:** Propionic acidemia, PA, Methylmalonic acidemia, MMA, Pathophysiology, Acute metabolic decompensation

## Abstract

**Background:**

Pathophysiology of life-threatening acute metabolic decompensations (AMD) in propionic acidemia (PA) and isolated methylmalonic acidemia (MMA) is insufficiently understood. Here, we study the metabolomes of PA and MMA patients over time, to improve insight in which biochemical processes are at play during AMD.

**Methods:**

Longitudinal data from clinical chemistry analyses and metabolic assays over the life-course of 11 PA and 13 MMA patients were studied retrospectively. Direct-infusion high-resolution mass spectrometry was performed on 234 and 154 remnant dried blood spot and plasma samples of PA and MMA patients, respectively. In addition, a systematic literature search was performed on reported biomarkers. All results were integrated in an assessment of biochemical processes at play during AMD.

**Results:**

We confirmed many of the metabolite alterations reported in literature, including increases of plasma valine and isoleucine during AMD in PA patients. We revealed that plasma leucine and phenylalanine, and urinary pyruvic acid were increased during AMD in PA patients. 3-hydroxyisovaleric acid correlated positively with plasma ammonia. We found that known diagnostic biomarkers were not significantly further increased, while intermediates of the branched-chain amino acid (BCAA) degradation pathway were significantly increased during AMD.

**Conclusions:**

We revealed that during AMD in PA and MMA, BCAA and BCAA intermediates accumulate, while known diagnostic biomarkers remain essentially unaltered. This implies that these acidic BCAA intermediates are responsible for metabolic acidosis. Based on this, we suggest to measure plasma 3-hydroxyisovaleric acid and urinary ketones or 3-hydroxybutyric acid for the biochemical follow-up of a patient’s metabolic stability.

## Introduction

Propionic acidemia (PA) and isolated methylmalonic acidemia (MMA) are disorders affecting the catabolic pathway of the branched-chain amino acids (BCAA) L-isoleucine and L-valine, and the amino acids L-threonine and L-methionine. PA is caused by a deficiency of propionyl-CoA carboxylase (encoded by *PCCA* and *PCCB*), and isolated MMA is either caused by a deficiency of methylmalonyl-CoA mutase, methylmalonyl-CoA epimerase or by a defect in the metabolism of the cofactor of methylmalonyl-CoA mutase, 5′-deoxyadenosylcobalamin (encoded by *MUT, MCEE, MMAA, MMAB or MMADHC*, respectively).

The clinical course of PA and MMA is characterized by life-threatening acute metabolic decompensations (AMD). Clinically, AMD result in lethargy, anorexia, vomiting, dehydration, hypotonia, Kussmaul breathing and potentially coma and even death. Biochemically, AMD are characterized by hyperammonemia, metabolic acidosis with a high anion gap and lactic acidosis [1, 2]. AMD are held responsible for the neurological deficits that patients present with, such as psychomotor retardation, cognitive impairment, movement disorders and epilepsy [3].

AMD are thought to arise as a result of a catabolic stressor, which induces protein catabolism and increases the load of toxic metabolites, causing clinical distress. However, despite increasing knowledge on specific metabolite alterations during these decompensations, pathophysiology of AMD is not entirely understood. Plasma ammonia for example, is considered the best available biochemical read-out of AMD, but does not provide insight in other pathophysiological processes that may occur during AMD. Increased insight in the exact pathophysiological events during AMD could potentially pave the way towards optimized clinical care and better neurological outcomes [4, 5].

To increase pathophysiological understanding of AMD, we here study the metabolomes throughout life of 24 PA and MMA patients. Hereto, longitudinal results from targeted biochemical assays and from untargeted metabolomics were analyzed, and integrated with the results of a systematic search for biomarkers reported in literature.

## Materials and methods

### Patient inclusion

Patients were eligible for inclusion when PA or isolated MMA was enzymatically or genetically confirmed (patients with combined methylmalonic aciduria and homocystinuria were excluded), and when targeted metabolic analyses had been performed or when remnant samples were available at the University Medical Centre Utrecht. All 24 included patients or their legal guardians provided written approval for the analysis of their medical records and the use of their remnant samples for this research. All procedures followed were in accordance with the ethical standards of the University Medical Centre Utrecht (17–490/C) and with the Helsinki Declaration of 1975, as revised in 2000.

Eleven patients were diagnosed with PA and 13 with MMA, of whom four were nonresponsive and nine were responsive to cobalamin supplementation (Table 1). Median age at follow-up was 17.9 and 11.9 years for PA and MMA patients, respectively (Table 1). Three PA patients and one MMA patient died (Table 1). Patients were treated in line with current treatment protocols [3].

**Table 1:** patient and sample inclusion

**Patient**	**Sex**	**Age (y)**	**Gene**	**Cobalamin resp**	**Presentation**	**Number of AMD per patient year**	**Number of samples**											
							*Targeted analysis clinical laboratory*			*Targeted metabolic assays*			*Untargeted DI-HRMS in plasma*			*Untargeted DI-HRMS in DBS*		
							Total	No AMD	AMD	Total	No AMD	AMD	Total	No AMD	AMD	Total	No AMD	AMD
P.01	F	19.6	*MUT*	No	Early	0.4	217	46	19	96	43	5	20	11	1	15	0	1
P.02	M	11.9	*MUT*	No	Late	0.9	43	28	0	69	59	0	20	19	0	11	10	0
P.03	F	11.0	*MUT*	Yes	Late	0.1	6	6	0	18	17	1	8	8	0	7	7	0
P.04	F	7.2	*MUT*	No	Late	0.5	1	1	0	2	2	0	1	1	0	0	0	0
P.05	M	7.5	*MUT*	Yes	Late	2.1	1	0	0	6	0	0	0	0	0	0	0	0
P.06	F	20.2	*MMAA*	Yes	Early	0.1	79	52	1	76	59	1	18	14	0	7	7	0
P.07^a^	F	16.2	*MMAA*	Yes	Early	0.1	87	37	2	57	41	0	6	4	0	3	3	0
P.08^a^	M	^b^, 0.3	*MMAA*	Yes	Late		0	0	0	2	0	0	0	0	0	0	0	0
P.09	M	13.9	*MMAA*	Yes	Late	0.1	7	7	0	7	7	0	2	2	0	2	2	0
P.10	F	11.1	*MMAA*	Yes	Late	0.1	44	0	3	60	1	1	13	0	1	19	1	1
P.11	F	40.6	*MMAB*	Yes	Late	0.0	25	10	0	16	11	0	2	1	0	0	0	0
P.12	M	0.1	*MUT*	No	Early		0	0	0	7	0	0	0	0	0	0	0	0
P.13	M	37.3	MMA, unclass	Yes	Early	0.0	0	0	0	19	0	0	0	0	0	0	0	0
							**510**	**187**	**25**	**435**	**240**	**8**	**90**	**60**	**2**	**64**	**30**	**2**
P.14	F	23.1	PA, unclass		Early	0.6	369	18	38	106	3	14	18	0	2	7	0	1
P.15^a^	F	^b^, 2.9	PA, unclass		Early	4.1	33	28	3	0	0	0	1	1	0	0	0	0
P.16^a^	M	12.5	PA, unclass		Family	2.4	92	6	19	21	3	3	9	1	2	13	0	2
P.17	F	17.6	*PCCB*		Early	0.7	206	6	23	92	0	9	29	9	4	24	0	4
P.18	F	17.9	*PCCA*		Early	0.7	251	64	31	128	50	12	37	11	4	30	0	4
P.19	F	8.6	*PCCB*		Early	2.6	157	5	36	14	1	3	9	1	3	7	0	2
P.20	M	^b^, 7.5	*PCCB*		Early	0.3	0	0	0	19	0	0	0	0	0	0	0	0
P.21	F	24.6	*PCCA*		Late	0.2	16	0	0	7	0	0	1	0	0	0	0	0
P.22^a^	M	^b^, 19.1	*PCCB*		Late	0.3	80	1	2	72	2	0	12	0	0	0	0	0
P.23^a^	M	19.1	*PCCB*		Family	0.1	62	32	4	61	32	1	19	9	0	11	0	0
P.24	M	20.4	*PCCA*		Late	0.2	11	0	1	7	0	0	4	0	0	3	0	0
							**1277**	**160**	**157**	**527**	**91**	**42**	**139**	**23**	**15**	**95**	**0**	**13**

Subtotals are depicted in bold. ^a^P.07 and P.08, P.15 and P.16, and P.22 and P.23 are pairs of siblings. Early onset: presentation < 28 days of life; Late onset: presentation > 28 days of life. Family: diagnosis at birth through an affected sibling. *Abbreviations*: *AMD* acute metabolic decompensation, *F* female, *M* male, *unclass* genetic defect is unclassified, (*y*) years; ^b^passed away

### Targeted biochemical analyses

Available results of all targeted analyses performed throughout the patient’s life in the clinical chemical laboratory and in the metabolic diagnostic laboratory of the University Medical Centre Utrecht were systematically retrieved (Table 1).

### Untargeted biochemical analyses: direct-infusion high-resolution mass spectrometry

Blood samples were drawn and stored as described before [6]. DI-HRMS was performed on 234 remnant DBS and plasma samples of PA patients and on 154 remnant DBS and plasma samples of MMA patients (Table 1), as described before [6]. DI-HRMS analysis resulted in the identification of 1905 mass peaks that could be annotated as 3929 metabolites that are expected to occur endogenously. For each mass peak per patient sample, the deviation from the intensities in the 30 control samples was indicated by a Z-score [6]. Z-scores were calculated for both patient and control samples and were considered aberrant when > 2.0 or < − 1.5.

### Data analysis

A sample was classified as ‘no AMD’ when the sample was obtained at an outpatient, scheduled visit that did not result in hospitalization. A sample was classified as ‘AMD’ when the sample was drawn on the first day of admission, when the patient was hospitalized for an (impending) AMD. All other samples, for example samples drawn during hospitalizations but not on the first day, were considered neither ‘no AMD’ nor ‘AMD’ (Table 1).

To identify diagnostic biomarkers in targeted analyses, a metabolite was considered a biochemical disease marker when the median was below the lower limit of the reference range (RR), or above the upper limit of the RR. For DI-HRMS, Mann-Whitney U tests were performed comparing samples classified as ‘no AMD’ to control samples, for PA and MMA separately.

To identify metabolites associated with metabolic instability, Mann-Whitney U tests were performed comparing samples classified as ‘AMD’ with samples classified as ‘no AMD’, for both PA and MMA separately. In addition, Spearman correlation tests were performed comparing concentrations and Z-scores of every measured metabolite in every patient in this study to plasma ammonia concentrations.

*P*-values were adjusted according to the Bonferroni method, and considered statistically significant when < 0.05. R^2^ values were considered biologically relevant when > 0.50 or < − 0.50. Data analysis was performed in R programming language. Data and R code are available on request.

Supervised clustering analyses were performed using the software of MetaboAnalyst 4.0 [7]. Z-score tables with unpaired samples in columns were uploaded. No missing value estimation, data filtering or normalization was performed. The analysis paths ‘Partial Least Squares – Discriminant Analysis’ (PLS-DA) including the 2D scores plot and the variable importance in projection score, and heatmaps were analyzed.

### Literature study

To obtain an overview on known diagnostic biomarkers and on biochemical parameters reported to be associated with AMD in PA and MMA, we performed an extensive literature search [4, 5]. All reports discussing biomarkers were systematically evaluated and results were tabulated in Table S1 [1, 2, 8–31]. This table lists, to the best of our knowledge, the first reports of diagnostic biomarkers, of biochemical parameters associated with the presence of AMD and of biochemical parameters that correlate with plasma ammonia in PA and MMA [1, 2, 8–31].

### Integration of findings reported in literature and study results

**Fig. 1:**
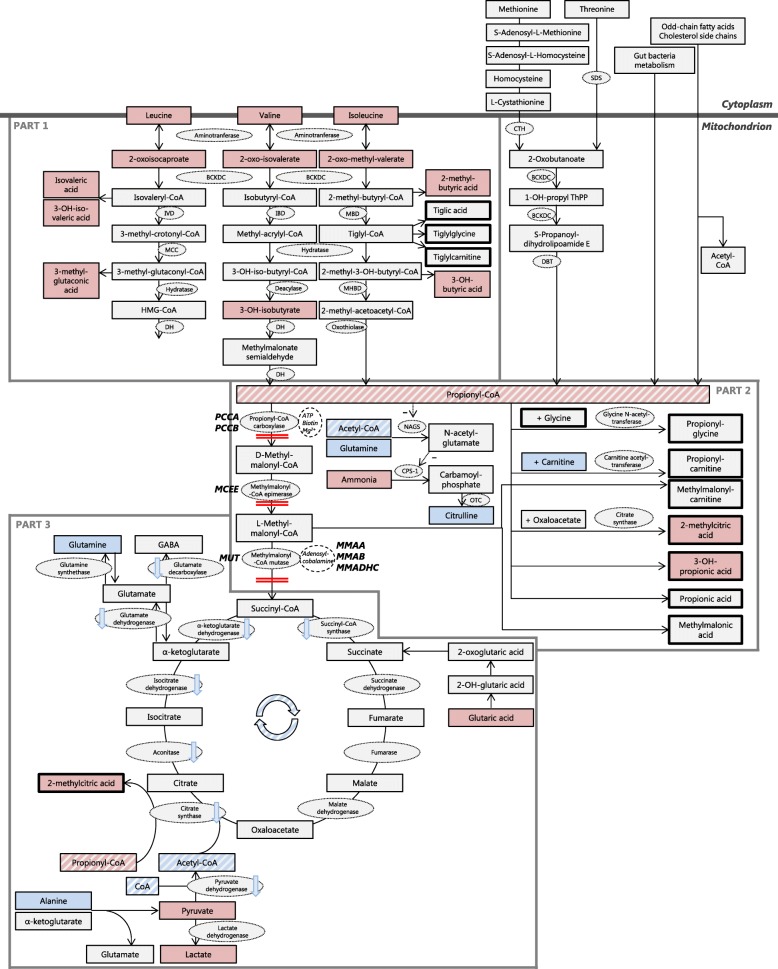
Alterations of the propionate pathway in times of acute metabolic decompensation. Metabolites are depicted in rectangles. Metabolites with significantly increased values during acute metabolic decompensations (AMD) are depicted in red, metabolites with normal values during AMD are depicted in light grey and metabolites with significantly decreased values during AMD are depicted in blue. The potential blockages of the pathway, due to enzyme deficiencies in PA and MMA, are depicted by double red lines. Genes involved in PA and MMA are depicted in bold capitals. Propionyl-CoA and acetyl-CoA, central metabolites in the pathway, are highlighted by diagonal stripes. Enzymes are depicted in light grey ovals, cofactors are depicted in white ovals. Decreased activity of enzymes is depicted by light blue arrows. The pathway is distinguished in three parts, indicated by dark gray lines. Cytoplasm is distinguished from the mitochondrion, indicated by a broad dark gray line. Abbreviations: BCKDC: branched-chain α- ketoacid dehydrogenase complex. CPS-1: carbamoyl phosphate synthase I. CTH: cystathionine gamma-lyase. DBT: dihydrolipoamide branched chain transacylase E2. DH: dehydrogenase. IBD: isobutyryl-CoA dehydrogenase. IVD: isovaleryl-CoA dehydrogenase. MBD: 2-methylbutyryl-CoA dehydrogenase. MCC: 3-methylcrotonyl-CoA carboxylase. MHBD: 2-methyl-3-hydroxy-butyryl-CoA dehydrogenase. NAGS: N-acetylglutamate synthase. OTC: ornithine transcarbamylase. SDS: L-serine dehydratase

Findings reported in the reviewed literature as listed in Table S1 [1, 2, 8–31] were combined with the results obtained in this study and visualized in an overview of the catabolic pathway of L-isoleucine, L-valine, L-threonine and L-methionine, derived from Fig. 1 and 2 of Haijes et al. 2019 [5], to obtain an overview of the all known metabolite alterations during AMD in PA and MMA. For each of the alterations, the significance for the pathophysiology of AMD was assessed.

Since we did not quantify propionyl-CoA or acetyl-CoA, propionylcarnitine and acetylcarnitine were used as proxy to estimate these concentrations. In healthy individuals, acetylcarnitine is 10-fold higher than propionylcarnitine (RR 0.7–9.7 μmol/L for acetylcarnitine, RR 0.0–0.8 μmol/L for propionylcarnitine). Extrapolation of these concentrations suggests that the C2/C3 ratio in healthy individuals approximates 10. The C2/C3 ratio in PA and MMA patients was calculated based on targeted quantification of acetylcarnitine and propionylcarnitine.

## Results

### Confirmation of metabolite alterations already reported in literature

Targeted metabolic assays in the included samples confirmed abnormalities in 13 diagnostic biomarkers reported in the reviewed literature, of which six are shared between PA and MMA, three were only observed in PA patients and four were only observed in MMA patients (Table S2, Figure S1). Untargeted DI-HRMS confirmed six disease biomarkers, three of which are shared between PA and MMA, two were only observed in PA patients and one was only observed in MMA patients (Table S2, Figure S2-S3). PLS-DA of untargeted DI-HRMS data from both DBS and plasma confirmed propionylcarnitine, propionylglycine, glycine, 3-dehydroxycarnitine and 2-methylcitric acid as biomarkers for PA (Figure S4-S11), and methylmalonic acid, propionylcarnitine, glycine, 2-methylcitric acid, 3-dehydroxycarnitine and methylmalonyl-carnitine as biomarkers for MMA (Figure S12-S17).

With respect to the samples obtained during AMD, clinical chemistry tests confirmed that urea was increased in MMA patients (Table 2, Figure S18-S19) and that ammonia was increased in PA patients (Table 2, Figure S18, S20). Targeted metabolic assays confirmed the increases of valine and isoleucine, the decreases of glutamine, citrulline and free carnitine in plasma, and the increase of lactic acid and 3-hydroxybutyric acid in urine, in PA patients during AMD (Table 2, Figure S21-S29). Untargeted DI-HRMS confirmed 3-hydroxyisovaleric acid (10 isomers) as a biochemical marker increased during AMD in PA patients (Table 2, Figure S30-S31). Targeted metabolic assays and untargeted DI-HRMS disclosed that 2-methylcitric acid, 3-hydroxypropionic acid and propionylglycine correlated positively with plasma ammonia (Table 3, Figure S32-S33).

**Table 2:** biochemical parameters associated with presence of AMD for PA and MMA

**Analyte**	**Matrix**	**PROPIONIC ACIDURIA**				**METHYLMALONIC ACIDURIA**			
		**No AMD**	**AMD**	*P-value*	*Known*	**No AMD**	**AMD**	*P-value*	*Known*
		*Median + SD [Min-Max] (N)*	*Median* *+* *SD [Min-Max] (N)*			*Median* *+* *SD [Min-Max] (N)*	*Median* *+* *SD [Min-Max] (N)*		
Clinical chemistry									
Urea	Plasma	3.5 + 1.8 [0.4–8.0] (84)	6.3 + 2.9 [0.6–15.3] (103)	< 0.0001	No	3.0 + 1.6 [1.0–8.2] (93)	9.1 + 8.0 [1.8–25.3] (10)	0.0043	Yes
Glucose	Plasma	5.1 + 1.1 [3.8–10.9] (104)	6.2 + 5.4 [1.2–44.8] (194)	< 0.0001	Yes	5.2 + 1.1 [3.7–11.2] (86)	6.1 + 1.2 [4.8–9.5] (27)	0.0288	No
Ammonia	Plasma	75 + 41 [9–222] (107)	115 + 221 [21–1807] (170)	< 0.0001	Yes	45 + 22 [13–97] (66)	49 + 33 [20–161] (22)	NS	
Calcium ionized	Plasma	1.30 + 0.06 [1.09–1.38] (33)	1.22 + 0.08 [0.91–1.32] (75)	0.0001	No	1.27 + 0.05 [1.07–1.35] (40)	1.22 + 0.06 [1.14–1.32] (10)	NS	
Targeted metabolic assays									
Leucine	Plasma	59 + 23 [31–152] (73)	113 + 65 [16–318] (34)	< 0.0001	No	77 + 40 [34–223] (77)	97 + 11 [89–104] (2)	NS	
Phenylalanine	Plasma	37 + 8 [26–61] (73)	54 + 22 [32–146] (34)	0.0001	No	45 + 14 [24–110] (77)	77 + 16 [65–88] (2)	NS	
Valine	Plasma	66 + 26 [20–183] (73)	121 + 72 [25+ 344] (34)	0.0019	Yes	107 + 42 [41–217] (77)	143 + 11 [135–150] (2)	NS	
Isoleucine	Plasma	21 + 9 [7–48] (73)	40 + 28 [8–131] (29)	0.0104	Yes	32 + 15 [7–84] (77)	43 + 12 [34–51] (2)	NS	
Lactic acid	Urine	46 + 206 [18–1067] (30)	878 + 3462 [201–11,273] (16)	< 0.0001	Yes	41 + 314 [2–3424] (149)	123 + 133 [40–425] (7)	NS	
3-Hydroxybutyric acid	Urine	10 + 190 [2–766] (16)	366 + 1311 [152–5284] (15)	0.0052	Yes	9 + 503 [0–3377] (50)	14 + 2867 [9–6436] (5)	NS	
Pyruvic acid	Urine	29 + 24 [4–121] (24)	103 + 180 [23–712] (15)	0.0115	No	15 + 12 [0–76] (72)	33 + 13 [11–45] (5)	NS	
Glutamine	Plasma	494 + 105 [252–793] (73)	383 + 110 [189–698] (34)	0.0066	Yes	380 + 126 [190–736] (77)	303 + 13 [294–312] (2)	NS	
Citrulline	Plasma	28 + 8 [6–50] (73)	17 + 11 [4–53] (34)	0.0073	Yes	24 + 16 [8–133] (77)	19 + 6 [14–23] (2)	NS	
C10-carnitine	Plasma	0.08 + 0.02 [0.04–0.14] (31)	0.04 + 0.03 [0.02–0.15] (22)	0.0296	No	0.16 + 0.79 [0.03–5.09] (68)	0.10 + 0.07 [0.04–0.18] (3)	NS	
Free carnitine	Plasma	38.9 + 16.8 [7.0–93.3] (56)	22.0 + 12.4 [8.0–70.1] (25)	0.0370	Yes	42.8 + 21.2 [3.0–105.7] (89)	45.5 + 66.4 [18.1–144.3] (3)	NS	
C8-carnitine	Plasma	0.05 + 0.01 [0.03–0.09] (29)	0.03 + 0.02 [0.01–0.10] (22)	0.0446	No	0.11 + 0.59 [0.03–3.69] (68)	0.09 + 0.04 [0.04–0.12] (3)	NS	
Homovanillic acid	Urine	8.3 + 7.5 [4.0–35.0] (22)	2.0 + 1.9 [1.0–7.0] (15)	0.0166	No	5.0 + 4.9 [1.0–27.5] (71)	1.0–5.0 [1.0–12.0] (5)	NS	
Untargeted DI-HRMS									
Acetylcysteine	Plasma	−0.2 + 1.6 [− 1.6–4.2] (23)	3.9 + 10.1 [0.5–41.6] (15)	0.0031	No	0.2 + 1.0 [− 2.2–2.7] (51)	0.9 + 1.6 [− 0.3–2.1] (2)	NS	
Cortisol (2 isomers)	Plasma	0.1 + 0.9 [− 1.8–0.8] (23)	1.6 + 1.1 [− 0.8–4.0] (15)	0.0208	No	0.1 + 1.0 [− 1.8–2.8] (51)	0.2 + 2.9 [− 1.9–2.2] (2)	NS	
3-Hydroxyisovaleric acid (10 isomers)	Plasma	0.0 + 7.5 [− 1.9–33.8] (23)	12.3 + 13.6 [4.3–58.7] (15)	0.0412	Yes	0.0 + 0.7 [− 0.6–3.4] (51)	1.9 + 2.7 [− 0.1–3.8] (2)	NS	
Fructoseglycine	Plasma	7.6 + 5.7 [− 0.8–25.8] (23)	0.9 + 2.6 [− 1.3–6.2] (15)	0.0073	No	2.2 + 2.2 [− 1.2–7.6] (51)	3.0 + 1.6 [1.8–4.1] (2)	NS	

Results of clinical chemistry are presented in mmol/L, except for ammonia for which results are presented in μmol/L. Results of targeted metabolic assays in plasma are presented in μmol/L, results of targeted metabolic assays in urine are presented in mmol/mol creatinine. All *p*-values were adjusted according to the Bonferroni method. A *p*-value < 0.05 was considered statistically significant. *Abbreviations DI-HRMS* direct-infusion high-resolution mass spectrometry, *Max* maximum value, *Min* minimum value, (*N*) number of samples, *NS* not significant, *SD* standard deviation

**Table 3:** biochemical parameters that correlate with plasma ammonia for PA and MMA

**Analyte**	**Matrix**	**N**	**R**^**2**^	***P*-value**	**Known**
Targeted metabolic assays					
Ketones	Plasma	5	1.00	< 0.0001	No
2-Methylcitric acid^a^	Plasma	42	0.77	< 0.0001	Yes
Arachidonic acid	Plasma	44	0.72	< 0.0001	No
3-Hydroxyisovaleric acid	Plasma	39	0.70	0.0058	No
2-Methylcitric acid^a^	Plasma	79	0.67	< 0.0001	Yes
3-Hydroxyisovaleric acid	Urine	163	0.67	< 0.0001	No
3-Hydroxypropionic acid	Plasma	46	0.66	< 0.0001	Yes
Glutaric acid	Urine	149	0.64	< 0.0001	No
Pipecolinic acid	Plasma	68	0.52	0.0432	No
Alanine/lysine ratio	Plasma	94	−0.68	< 0.0001	No
C4-DC carnitine	Plasma	225	−0.55	< 0.0001	No
C14:1 carnitine/C2 carnitine ratio	Plasma	88	−0.53	0.0011	No
Alanine/(phenylalanine+tyrosine) ratio	Plasma	94	−0.51	0.0013	No
Untargeted DI-HRMS					
2-Methylcitric acid (3 isomers)	Plasma	157	0.68	< 0.0001	Yes
Alanyl-Isoleucine (3 isomers)	Plasma	157	0.65	< 0.0001	No
3-Hydroxyisovaleric acid (10 isomers)	Plasma	157	0.64	< 0.0001	No
3-Methyl-2-oxovaleric acid (7 isomers)	DBS	122	0.56	< 0.0001	No
Isobutyrylglycine (6 isomers)	Plasma	157	0.55	< 0.0001	No
Indole-5,6-quinone	DBS	122	0.53	< 0.0001	No
Propionylglycine (9 isomers)	DBS	122	0.52	< 0.0001	Yes
3-Hydroxyphenylacetic acid (3 isomers)	Plasma	157	0.51	< 0.0001	No
Indole-5,6-quinone	Plasma	157	0.51	< 0.0001	No
Pyrocatechol sulfate	DBS	122	−0.65	< 0.0001	No
Threonic acid	DBS	122	−0.60	< 0.0001	No
Trimethylamine N-oxide	DBS	122	−0.59	< 0.0001	No
Stearoylcarnitine	DBS	122	−0.58	< 0.0001	No
Methylmalonic acid (3 isomers)	DBS	122	−0.57	< 0.0001	No
Ergothioneine	DBS	122	−0.55	< 0.0001	No

All *p*-values were adjusted according to the Bonferroni method. A *p*-value < 0.05 was considered statistically significant. An R^2^ value of > 0.50 or < − 0.50 was considered biologically relevant. ^a^Due to methodological developments over time, two different diagnostic assays for 2-methylcitric acid were included in the analysis, both demonstrating a solid positive correlation with plasma ammonia. *Abbreviations DI-HRMS* direct-infusion high-resolution mass spectrometry, *N* number of samples

### Identification of novel metabolite alterations

Targeted analysis of amino acids revealed that histidine was significantly decreased in (treated) PA patients and glutamine was significantly decreased in (treated) MMA patients (Table S2, Figure S1, S24, S34). Histidine was also relatively decreased in MMA, and glutamine in PA, although the medians were not below the lower limit of the RR (Table S2, Figure S1, S24, S34). Untargeted DI-HRMS showed that histidine and glutamine were significantly decreased in both PA and MMA patients (Table S2, Figure S2). In addition, untargeted DI-HRMS unveiled 17 unreported potential diagnostic biomarkers (Table S2, Figure S2-S3) including increased lysoPC (15:0) (2 isomers), lysoPC (17:0) (2 isomers) and 2-amino-3-phosphonopropionic acid, especially in PA patients, and increased propionic acid (2 isomers) in MMA patients (Table S2, Figure S35-S39). PLS-DA of untargeted DI-HRMS data from both DBS and plasma confirmed 2-amino-3-phosphopropionic acid and lysoPC (15:0) as biomarkers for PA, and propionic acid as biomarker for MMA (Figure S4, S12), supporting that these three metabolites could serve as diagnostic biomarkers.

Investigation of biochemical parameters associated with AMD disclosed that urea, as already reported for MMA, was increased in PA patients during AMD (Table 2, Figure S18-S19). In addition, glucose was slightly increased in both PA and MMA patients (Table 2, Figure S18, S20) and ionized calcium was decreased in PA patients during AMD (Table 2, Figure S18). Targeted metabolic assays uncovered six unreported biochemical parameters altered during AMD in PA patients.

Leucine and phenylalanine in plasma and pyruvic acid in urine were increased (Table 2, Figure S21, S39-S42), and C8- and C10-carnitine in plasma and homovanillic acid were decreased (Table 2, Figure S21). Untargeted DI-HRMS revealed an increase of acetylcysteine and cortisol, and a normalization of fructoseglycine during AMD in PA patients (Table 2), but PLS-DA of untargeted DI-HRMS data from both DBS and plasma demonstrated that AMD could not be distinguished clearly from no AMD, based on the patients’ metabolomes (Figure S43-S44).

Both targeted metabolic assays and untargeted DI-HRMS showed that 3-hydroxyisovaleric acid correlated positively with plasma ammonia (Table 3, Figure S30-S33). In addition, two biochemical parameters for mitochondrial disease were found to negatively correlate with ammonia: alanine/lysine ratio and alanine/(phenylalanine+tyrosine) ratio (Table 3, Figure S32).

### Integration of findings reported in literature and study results

Metabolite alterations during AMD, either reported in the reviewed literature or reported here, are visualized in Fig. 1. Contrary to what is often expected, diagnostic biomarkers such as propionylglycine, propionylcarnitine, methylmalonylcarnitine, propionic acid and methylmalonic acid remained essentially unaltered during AMD (Fig. 1 – Part 2, Table 4), although for methylmalonic acid, the non-significant test results could be due to the small number of samples drawn during AMD (*n* = 2, Table 4). Rather, AMD appeared to induce an increase of BCAA and BCAA intermediates, all upstream metabolites of propionyl-CoA (Fig. 1 – Part 1). Based on this observation, we propose that the acidic BCAA intermediates are responsible for the metabolic acidosis in PA and MMA patients during AMD.

**Table 4:** diagnostic biomarkers for PA and MMA that are not significantly altered during AMD

**Analyte**	**Matrix**	**PROPIONIC ACIDURIA**			**METHYLMALONIC ACIDURIA**		
		**No AMD**	**AMD**	*P-value*	**No AMD**	**AMD**	*P-value*
		*Median* *+* *SD [Min-Max] (N)*	*Median* *+* *SD [Min-Max] (N)*		*Median* *+* *SD [Min-Max] (N)*	*Median* *+* *SD [Min-Max] (N)*	
Targeted metabolic assays							
Propionylcarnitine	Plasma	54.4 + 19.6 [23.0–99.5] (34)	49.7 + 25.6 [24.0–128.0] (23)	NS	21.0 + 21.7 [2.7–83.7] (68)	78.0 + 81.0 [14.8–175.5] (3)	NS
Glycine	Plasma	1381 + 362 [350–1962] (73)	1160 + 514 [265–2051] (35)	NS	492 + 369 [168–1916] (77)	334 + 152 [226–441] (2)	NS
Methylcitric acid	Plasma		49.5 + 14.8 [24.9–78.9] (12)	NS	2.2 + 2.8 [1.0–10.9] (11)		NS
Methylcitric acid	Urine	697 + 383 [425–968] (3)		NS			NS
3-Hydroxy-propionic acid	Urine	232 + 99 [89–349] (10)	738 + 355 [184–1534] (13)	NS	13.0 + 18.6 [0. – 86.0] (57)	9.5 + 76.8 [4.0–161.0] (4)	NS
Methylmalonylcarnitine	Plasma				7.6 + 7.7 [3.4–20.2] (5)		NS
Methylmalonic acid	Plasma				74 + 346 [3–1327] (52)		NS
Methylmalonic acid	Urine				727 + 4651 [64–21,587] (174)	4062 + 3200 [536–9044] (7)	NS
Acetylcarnitine	Plasma	10.1 + 7.5 [1.6–42.2] (34)	10.9 + 9.5 [1.3–43.9] (23)	NS	9.2 + 5.8 [2.1–29.9] (68)	12.6 + 24.4 [3.9–49.9] (3)	NS
Untargeted DI-HRMS							
Propionylcarnitine	Plasma	12.0 + 80.8 [− 2.8–363] (23)	272 + 193 [1.5–730] (15)	NS	37.9 + 171 [− 1.4–684] (61)	596 + 835 [5.7–1186] (2)	NS
Glycine	Plasma	9.5 + 4.8 [− 2.4–18.5] (23)	4.9 + 4.1 [− 0.4–14.7 (15)	NS	1.8 + 2.2 [− 1.1–8.5] (61)	2.7 + 3.0 [0.5–4.8] (2)	NS
2-Methylcitric acid (3 isomers)	Plasma	57.4 + 43.1 [− 1.8–147] (23)	93.7 + 31.4 [30.4–139] (15)	NS	9.0 + 9.5 [− 0.6–41.3] (61)	41.1 + 27.0 [22.1–60.2] (2)	NS
Propionylglycine (9 isomers)	Plasma	28.5 + 29.3 [− 3.0–91.1] (23)	80.6 + 50.1 [4.0–177.1] (15)	NS	1.6 + 3.1 [− 2.3–10.4] (61)	5.8 + 5.3 [2.0–9.6] (2)	NS
Methylmalonic acid (3 isomers)	Plasma				25.3 + 77.5 [− 0.0–376] (61)	150 + 175 [26.3–274] (2)	NS
Methylmalonylcarnitine	Plasma				2.4 + 9.1 [− 2.3–29.7] (61)	22.9 + 31.7 [0.4–45.3] (2)	NS

Results of targeted metabolic assays in plasma are presented in μmol/L, results of targeted metabolic assays in urine are presented in mmol/mol creatinine. All *p*-values were adjusted according to the Bonferroni method. A *p*-value < 0.05 was considered statistically significant. *Abbreviations DI-HRMS* direct-infusion high-resolution mass spectrometry, *Max* maximum value, *Min* minimum value, (*N*) number of samples, *NS* not significant, *P p*-value, *SD* standard deviation

Interestingly, intermediates of the leucine degradation pathway were elevated as well, even though leucine is not degraded via propionyl-CoA (Fig. 1 – Part 1). The increase of leucine and 2-oxoisocaproate could be explained by inhibition of the branched-chain α-ketoacid dehydrogenase complex (BCKDC) by -CoA esters, including propionyl-CoA [2, 32]. BCKDC is the rate-limiting enzyme in the BCAA catabolism pathway and is responsible for the irreversible step that converts branched-chain α-ketoacids into isobutyryl-CoA, 2-methyl-butyryl-CoA and isovaleryl-CoA. Increases of valine and isoleucine, as well as increases of 2-oxoisovalerate and 2-oxo-methylvalerate, have also been explained by inhibition of BCKDC (Fig. 1 – Part 1). However, inhibition of BCKDC cannot explain all reported increases of BCAA intermediates. Reasons for accumulation of metabolites downstream of BCKDC, such as 3-hydroxyisovaleric acid, 2-methyl-butyric acid and 3-methyl-glutaconic acid await further elucidation.

Next, the increase of plasma ammonia, and the relative decrease of plasma citrulline can be explained as follows. Propionyl-CoA inhibits N-acetylglutamate synthase and consequently, there is a lack of stimulation of carbamoylphosphate synthase [33]. This process might be aggravated by the relative decrease of acetyl-CoA and glutamine (Fig. 1 – Part 2). The result is decreased detoxification of ammonia [33] and also a relative lack of carbamoylphosphate. As carbamoylphosphate is the precursor of citrulline, we hypothesize that the observed decrease of plasma citrulline is caused by hampered citrulline formation by ornithine transcarbamylase (Fig. 1 – Part 2).

Downstream consequences of accumulation propionyl-CoA appear to be centered around a relative shortage of acetyl-CoA (Fig. 1 – Part 3). The median value of propionylcarnitine is five-fold higher than the median value of acetylcarnitine (Table 4) in PA and MMA patients. Hence, the C2/C3 carnitine ratio, which we use as proxy for the ratio of acetyl-CoA over propionyl-CoA, approximates 0.5, which is a 20-fold decrease compared to healthy individuals. We hypothesize that as a consequence of this relative shortage, the high levels of propionyl-CoA compete with the relatively low levels of acetyl-CoA for citrate synthase, resulting in formation of excessive amounts of 2-methylcitric acid formed from propionyl-CoA and oxaloacetate, rather than citrate (Fig. 1 – Part 3). Reduced conversion of acetyl-CoA and oxaloacetate into citrate could augment, in turn, depletion of citric acid cycle intermediates, potentially causing an energy deficiency (Fig. 1 – Part 3).

To replenish the citric acid cycle, we hypothesize that two different routes are being utilized. Firstly, in line with others, we hypothesize that glutamine, and possibly glutamate, might be used to replenish α-ketoglutarate, and that for this reason glutamine is decreased [1, 2, 28, 34] (Fig. 1 – Part 3). Secondly, alanine might be used for anaplerosis as well. In combination with a propionyl-CoA induced decreased activity of pyruvate dehydrogenase [5] this could result in the observed increase of both pyruvate and lactate (Fig. 1 – Part 3), which could explain the observed lactic acidosis.

## Discussion

Through an extensive literature study and a longitudinal analysis of the metabolomes of a cohort of PA and MMA patients, we revealed that during AMD, BCAA and BCAA intermediates accumulate, while known diagnostic biomarkers remain essentially unaltered. This implies that these acidic metabolites are responsible for metabolic acidosis. In addition, we speculated that downstream consequences of accumulating propionyl-CoA are centered around a relative shortage of acetyl-CoA, potentially resulting in depletion of citric acid cycle intermediates and thereby explaining the observed energy deficiency in PA and MMA patients. A relative shortage of acetyl-CoA could also explain the decrease of glutamine, decrease of alanine and the increases of pyruvate and lactate, thereby explaining the observed lactic acidosis.

### Biochemical analyses to perform during AMD

This study presents an overview of what is currently known on the biochemical processes during AMD in PA and MMA. More insight in the metabolic stability of a patient at a certain point in time can be attained by measuring plasma ammonia and lactate, pH, pCO_2_, bicarbonate and base excess. To improve insight in the metabolic consequences of AMD, we suggest to measure 2-methylcitric acid and 3-hydroxyisovaleric acid in plasma, as these parameters are significantly correlated to plasma ammonia and since 3-hydroxyisovaleric acid is significantly increased during AMD. In addition, we suggest to measure urinary ketones – which is already common practice by many clinicians – or specifically urinary 3-hydroxybutyric acid, as this metabolite is also significantly increased during AMD. Altogether, quantification of these markers could increase insight in the patient’s current metabolic stability. Conversely, we conclude that quantification of diagnostic biomarkers does not contribute to insight in the current metabolic stability of a patient.

Although concentrations were often still in the normal range, isoleucine, valine and leucine, as well as glutamine, citrulline and alanine could also be determined in times of AMD, as a trend analysis on an individual patient basis could increase insight in the biochemical processes during AMD in that patient. In addition, we advise to determine free carnitine concentrations to monitor whether carnitine supplementation is sufficient during AMD, as free carnitine is relatively decreased during AMD.

### Therapeutic interventions during AMD

Since free carnitine is relatively decreased during AMD, increasing free carnitine might improve scavenging of propionyl-CoA and methylmalonyl-CoA. This could be achieved by emergency (increase of) carnitine supplementation, possibly in a much earlier stage than at the time of hospital admission.

In addition, during AMD there is also a significant decrease of plasma citrulline, although the lack of citrulline is not as distinct as in urea cycle disorders. We speculate that citrulline supplementation, as provided during AMD in urea cycle disorders as N-acetylglutamate synthase deficiency, carbamoyl phosphate synthase I deficiency and ornithine transcarbamylase deficiency to maximize ammonia excretion through the urea cycle [35], might also contribute to ammonia detoxification in severely decompensated PA and MMA patients, but this hypothesis requires further study.

### Potential novel diagnostic biomarkers

Untargeted DI-HRMS revealed four metabolites that could potentially be new diagnostic biomarkers for PA and MMA. However, as diagnosing PA and MMA is often quite straightforward, the added value of these four metabolic markers for diagnostic purposes is limited. Though, these markers could point towards important pathophysiological processes in PA and MMA, and thereby they could potentially serve as markers for certain disease conditions, such as long-term metabolic control or neurological damage.

Firstly, lysoPC (15:0) and lysoPC (17:0), phospholipids with an odd-chain tail, were unveiled as potential disease biomarkers, especially in PA patients. It has been reported that increased intracellular concentration of propionyl-CoA leads to a relative abundance of odd-numbered long-chain fatty acids (LCFA) in body lipids, for example in erythrocyte membrane lipids. These odd-numbered LCFA seem to be increased even higher in patients with a more severe clinical course [36]. It has been hypothesized that odd-numbered LCFA are a reflection of continuous burden of propionyl-CoA toxicity within the cells, and that this might serve as a reliable tool for evaluating the quality of the long-term metabolic control [37]. In line with this, we observed that lysoPC (15,0) and lysoPC (17,0) were higher in patients that experienced AMD more frequently.

Secondly, in PA patients, 2-amino-3-phosphonopropionic acid (AP3) was found to be increased. It could not be distinguished whether this increase was caused by L-AP3, D-AP3 or a combination thereof. Yuan et al. found that D-AP3 does not induce any neurotoxic effects [38], but, in contrast, that L-AP3 is a stereoselective metabotropic excitatory glutamate receptor antagonist that blocks activation of excitatory glutamate receptors [38] and thereby increases activity of the *N-*acetyl-D-aspartate receptor [38–40]. Activation of the *N-*acetyl-D-aspartate receptor by propionic acid and methylmalonic acid has been described as an important pathophysiological process in inducing apoptosis of neurons and thereby causing neurological complications in PA and MMA [4, 5, 41], although the exact cascade is not fully understood. L-AP3 could be an intermediate in this process. This is further supported by a study demonstrating that intracaudatal injection of L-AP3 in rats caused a neurotoxic effect, characterized by vasogenic brain edema and neuronal degeneration, processes that are also described in PA and MMA [4]. An *N-*acetyl-D-aspartate receptor antagonist attenuated this effect. Although a potential role of L-AP3 in inducing neural degeneration is interestingly, this is still speculative.

Thirdly, a metabolite with a molecular weight corresponding to propionic acid and its endogenous isomers lactaldehyde and hydroxyacetone and its exogenous isomers ethyl formate, 3-hydroxypropanal and methyl acetate, was found to be markedly increased in MMA. Unexpectedly, this feature was not increased in PA. However, for none of the possible annotations of this m/z, a role in MMA but not in PA could be hypothesized. Since a very consistent correlation between methylmalonic acid and this metabolite was identified, both in DBS and plasma, especially for Z-scores > 50, we hypothesize that this metabolite could be a potential biomarker for MMA, and that this yet unannotated compound is a derivative of methylmalonic acid.

### Limitations and strengths

An important limitation of this study, inherent to studying rare diseases, is the limited sample cohort (Table 1). Almost all samples were drawn during dietary and/or pharmacological treatment, complicating pathophysiological interpretation. In addition, identification of metabolic markers characterizing AMD was affected by a smaller sample size, especially for MMA, for which no significant results were obtained for both targeted and untargeted analyses. Also, due to the retrospective design, samples were not obtained for every patient at similar time points. Moreover, our limited understanding of metabolic stability in PA and MMA patients impeded classification of samples as drawn during AMD or in times of metabolic stability. This may have contributed to the fact that we were not able to detect metabolic markers that could clearly distinguish AMD from no AMD. Furthermore, interpretation of historical data was affected by methodological developments, leading to different assays performed to quantify one metabolite, for example for 2-methylcitric acid. Lastly, the fact that due to direct-infusion an observed mass can account for multiple metabolite annotations, hampered solid conclusions on for example L-AP3 as potential biomarker for PA patients, and a metabolite with an m/z corresponding to propionic acid as potential biomarker for MMA patients. Therefore, the implications of these findings remain to be further elucidated.

Due to the limited sample size and variation in assays performed at each time point, we were not able to calculate the fraction of the anion gap of the accumulating BCAA intermediates during an AMD. To test our hypothesis that accumulation of BCAA intermediates is responsible for metabolic acidosis during AMD, we suggest to determine the fraction of the anion gap of BCAA intermediates during AMD in a prospective study.

Despite these limitations, important strengths of this study are that historical, longitudinal results from both clinical chemistry and targeted metabolic assays were combined with untargeted analyses in remnant samples, in order to search for potential disease biomarkers as broad as possible. Our approach accurately verified the findings of previous studies and we identified potentially novel diagnostic biomarkers for both PA and MMA. By combining our results with an extensive literature search on biomarkers for PA and MMA during AMD, we generated new hypotheses regarding which biochemical processes could be at play in the pathophysiology of AMD in PA and MMA.

## Conclusion

In conclusion, we here verify and expand reported findings on altered metabolites during AMD in PA and MMA. We illustrate in detail what could be important pathophysiological processes during AMD. Based on our findings, we propose that accumulating acidic BCAA intermediates may be held responsible for inducing metabolic acidosis during AMD, instead of propionic acid and methylmalonic acid, which are essentially unaltered during AMD.

## Supplementary information

**Additional file 1.**

**Additional file 2.**

## Data Availability

The datasets supporting the conclusions of this article are available from the corresponding author upon reasonable request.

## Abbreviations

AMD: Acute metabolic decompensation
AP-3: 2-amino-3-phosphonopropionic acid
BCAA: Branched-chain amino acids
BCKDC: Branched-chain α-ketoacid dehydrogenase complex
DBS: Dried blood spot
DI-HRMS: Direct-infusion high-resolution mass spectrometry
LCFA: Long-chain fatty acids
MMA: Methylmalonic acidemia
PA: Propionic acidemia
PLS-DA: Partial Least Squares – Discriminant Analysis
RR: Reference range

## List of human genes

*PCCA*: Propionyl-CoA carboxylase subunit alpha
*PCCB*: Propionyl-CoA carboxylase subunit beta
*MMUT*: Methylmalonyl-CoA mutase, also commonly referred to as *MUT*
*MCEE*: Methylmalonyl-CoA epimerase
*MMAA*: Metabolism of cobalamin associated A
*MMAB*: Metabolism of cobalamin associated B
*MMADHC*: Metabolism of cobalamin associated D
