# Supplementary material for: Understanding acute metabolic decompensation in propionic and methylmalonic acidemias: a deep metabolic phenotyping approach
Source: Orphanet J Rare Dis. 2020 Mar 6;15:68. doi: 10.1186/s13023-020-1347-3 (PMC7060614; doi:10.1186/s13023-020-1347-3)

Supplementary Figure 1

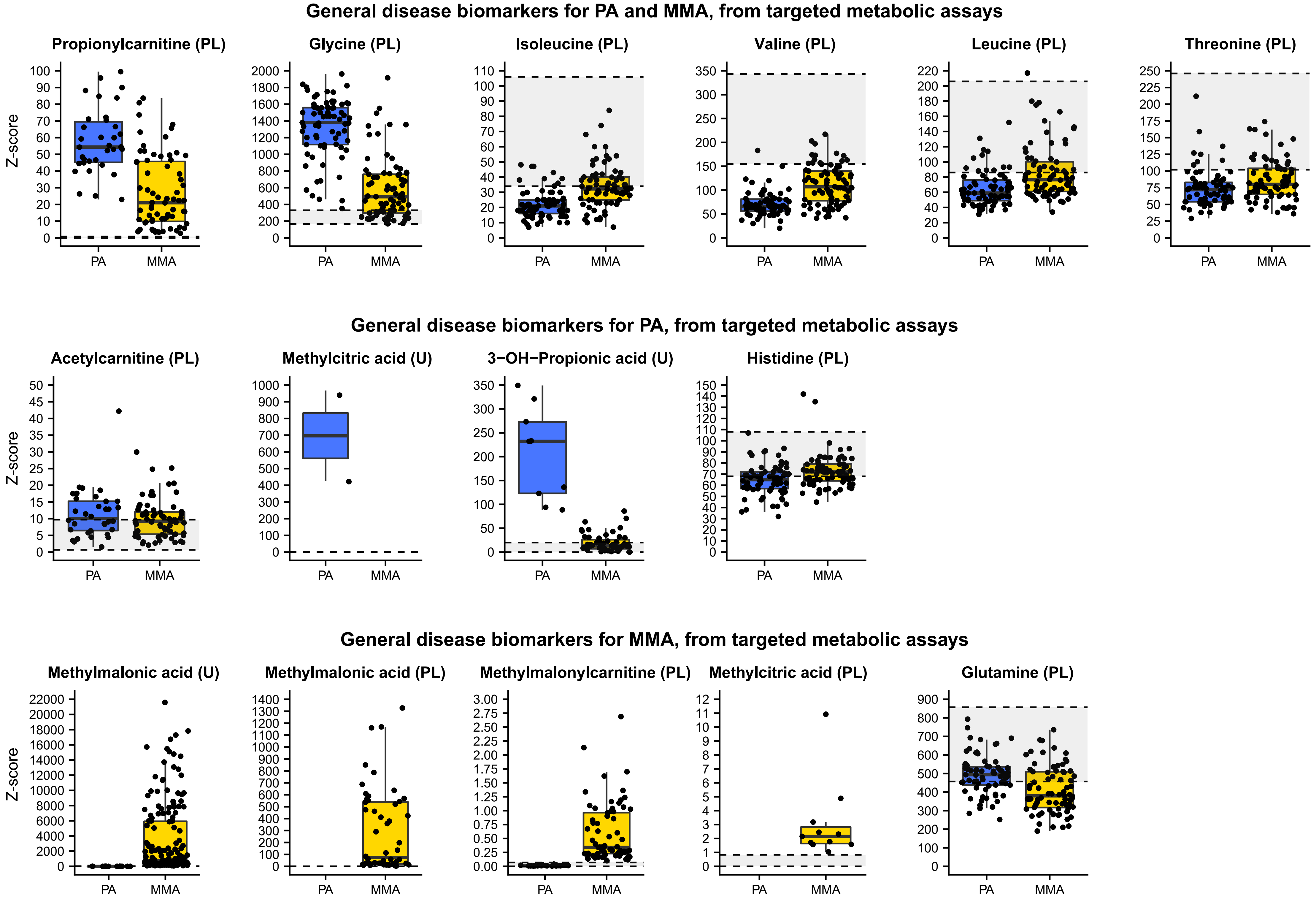

Supplementary Figure 2

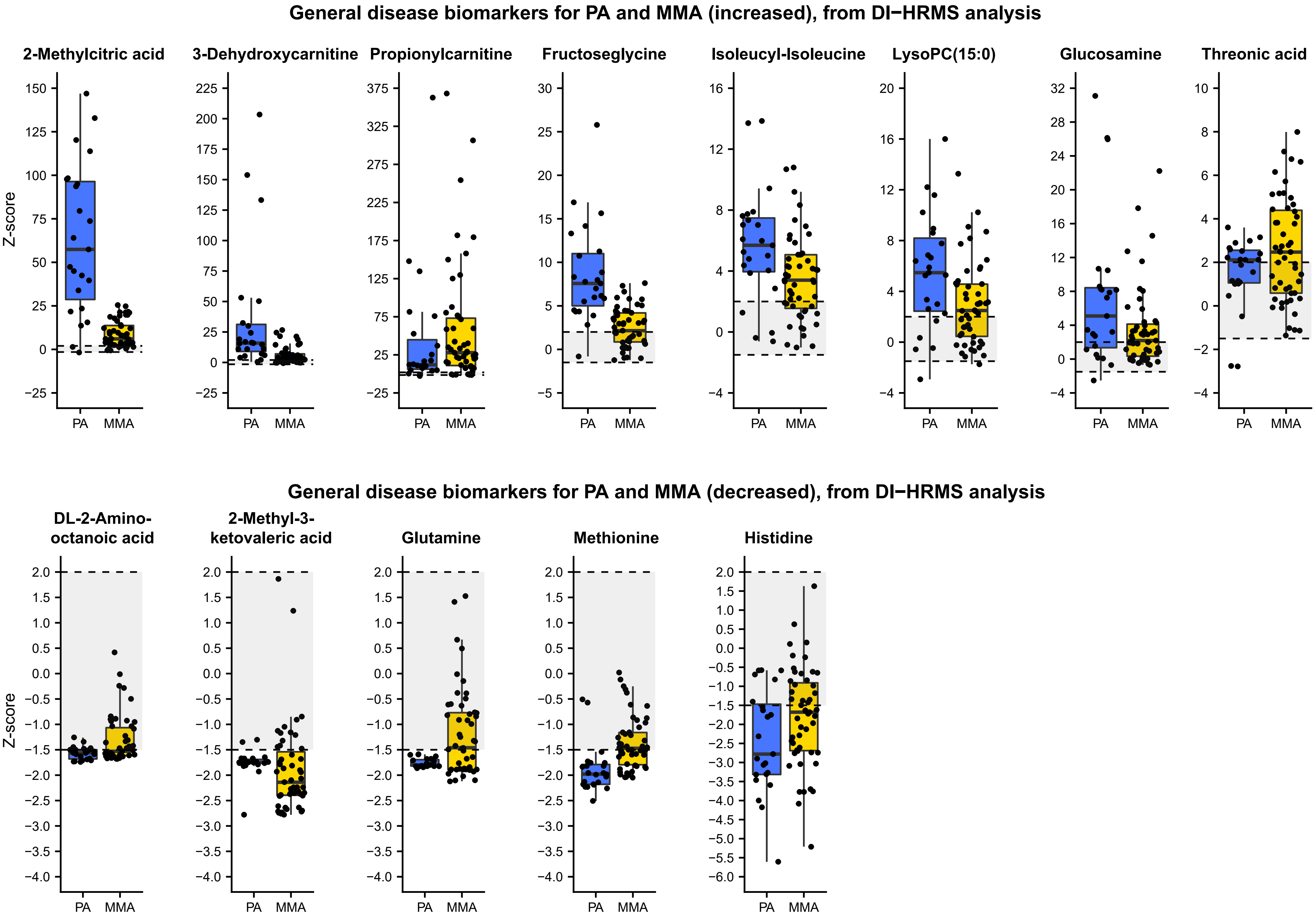

Supplementary Figure 3

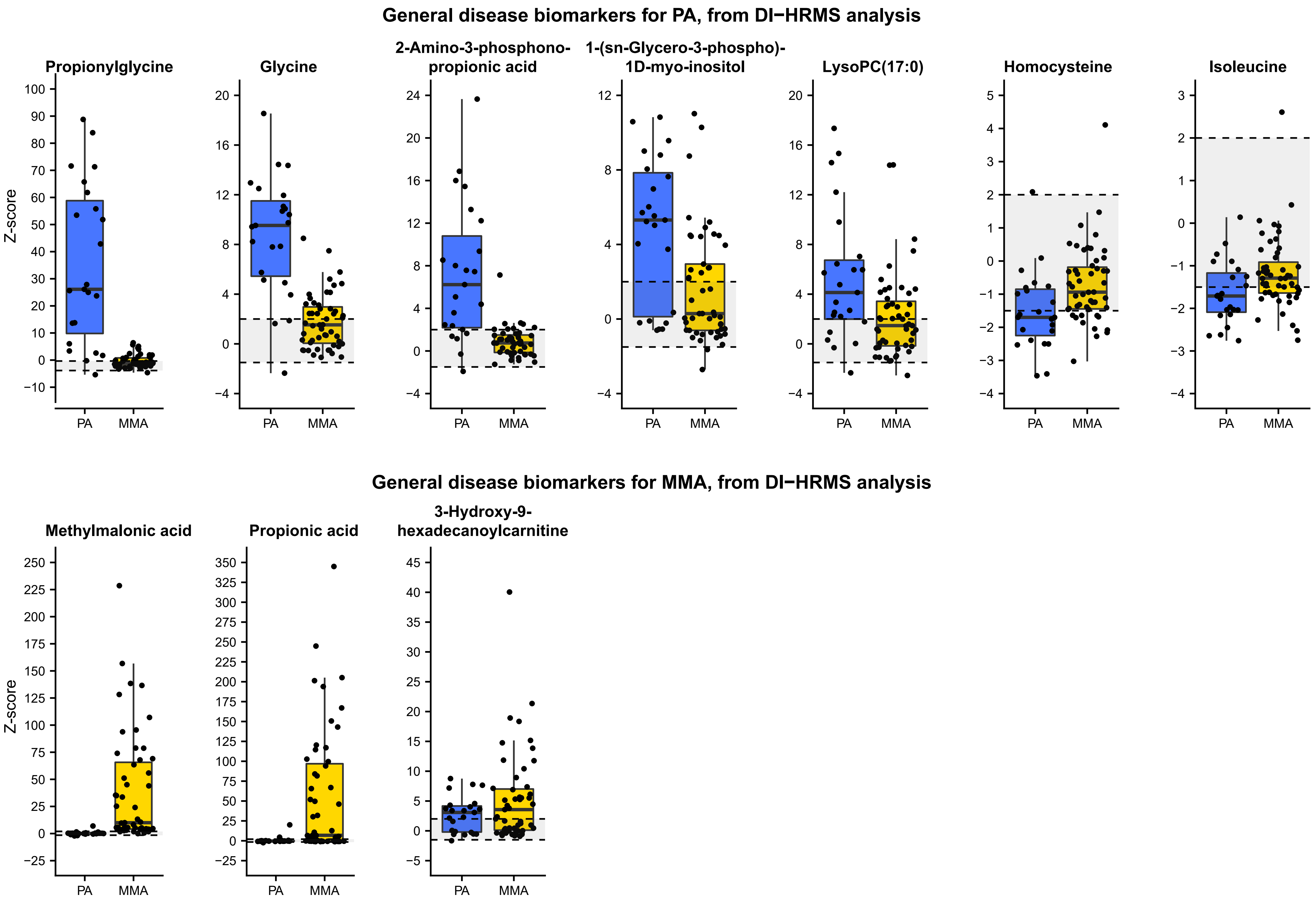

Supplementary Figure 4

PLS-DA, VIP-scores and heatmap for PA samples versus control samples

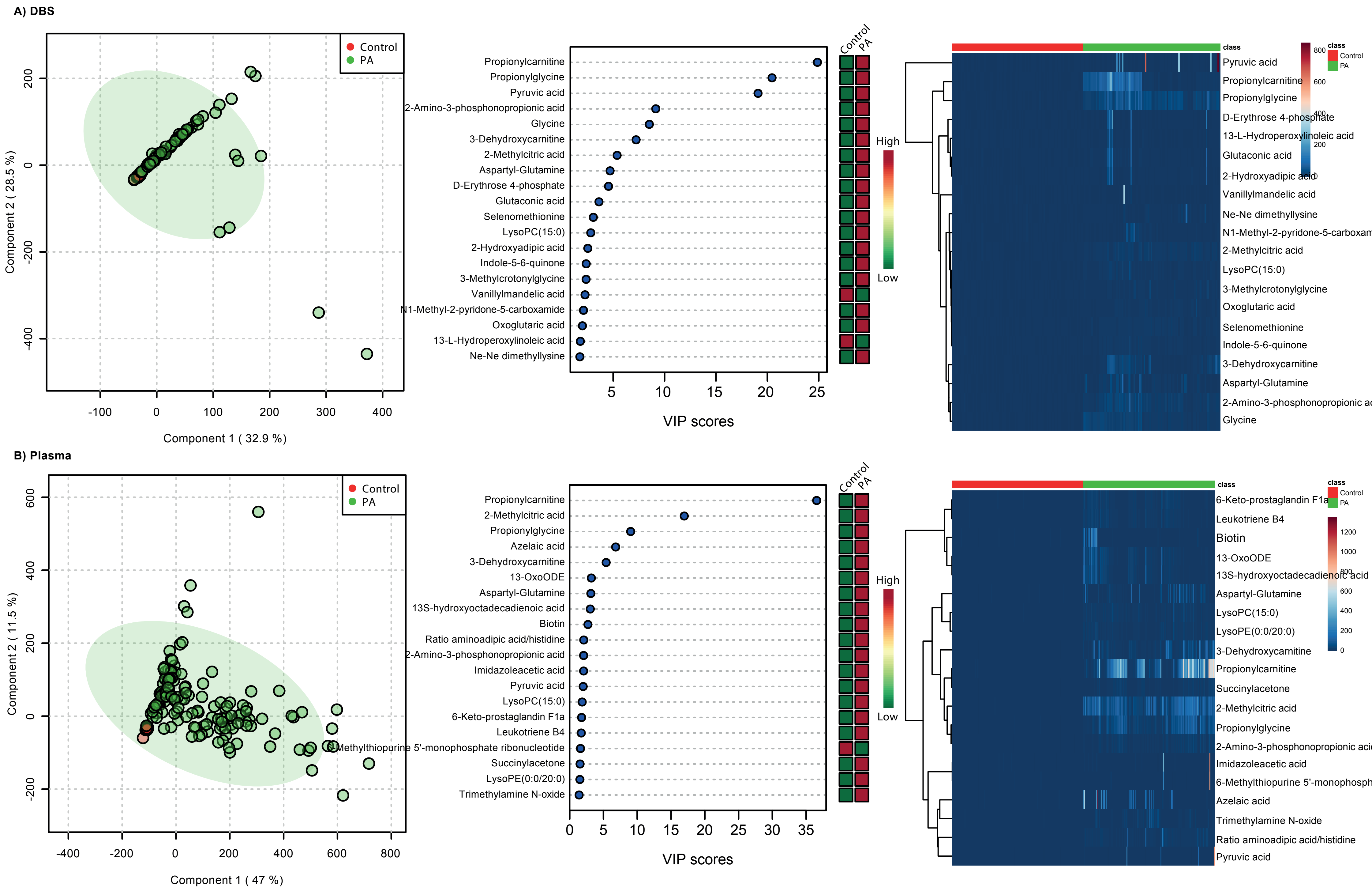

Supplementary Figure 5

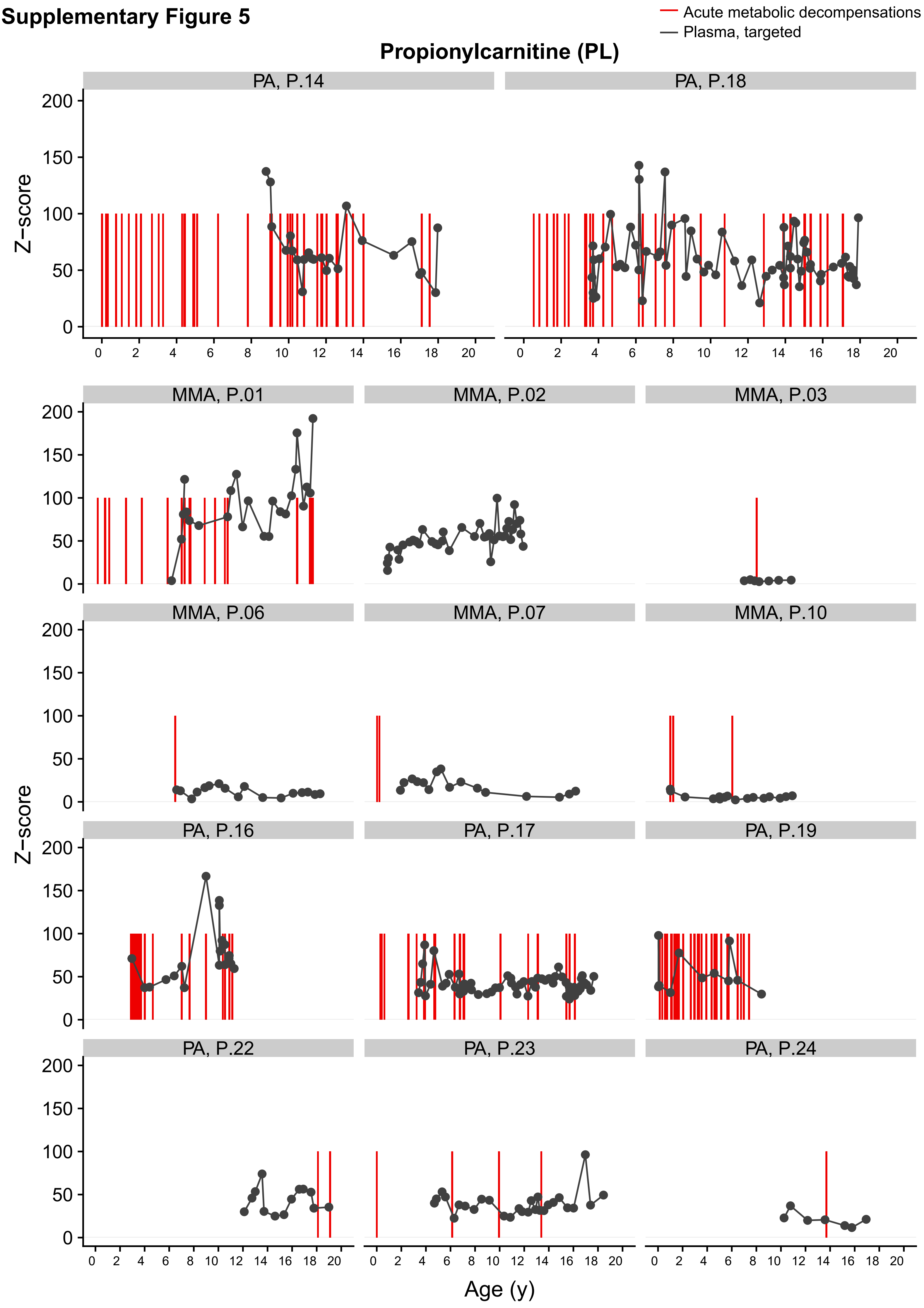

Supplementary Figure 6

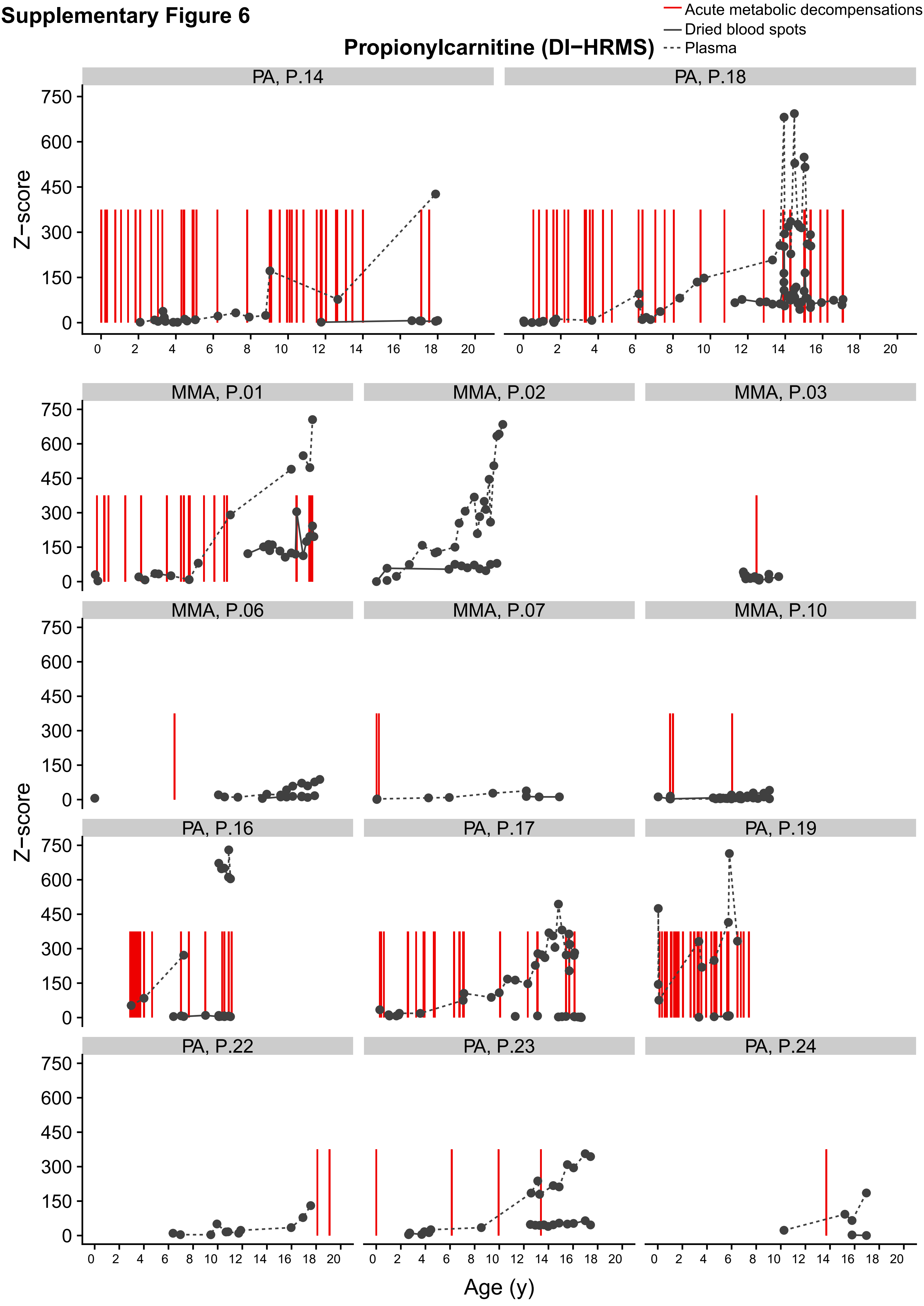

Supplementary Figure 7

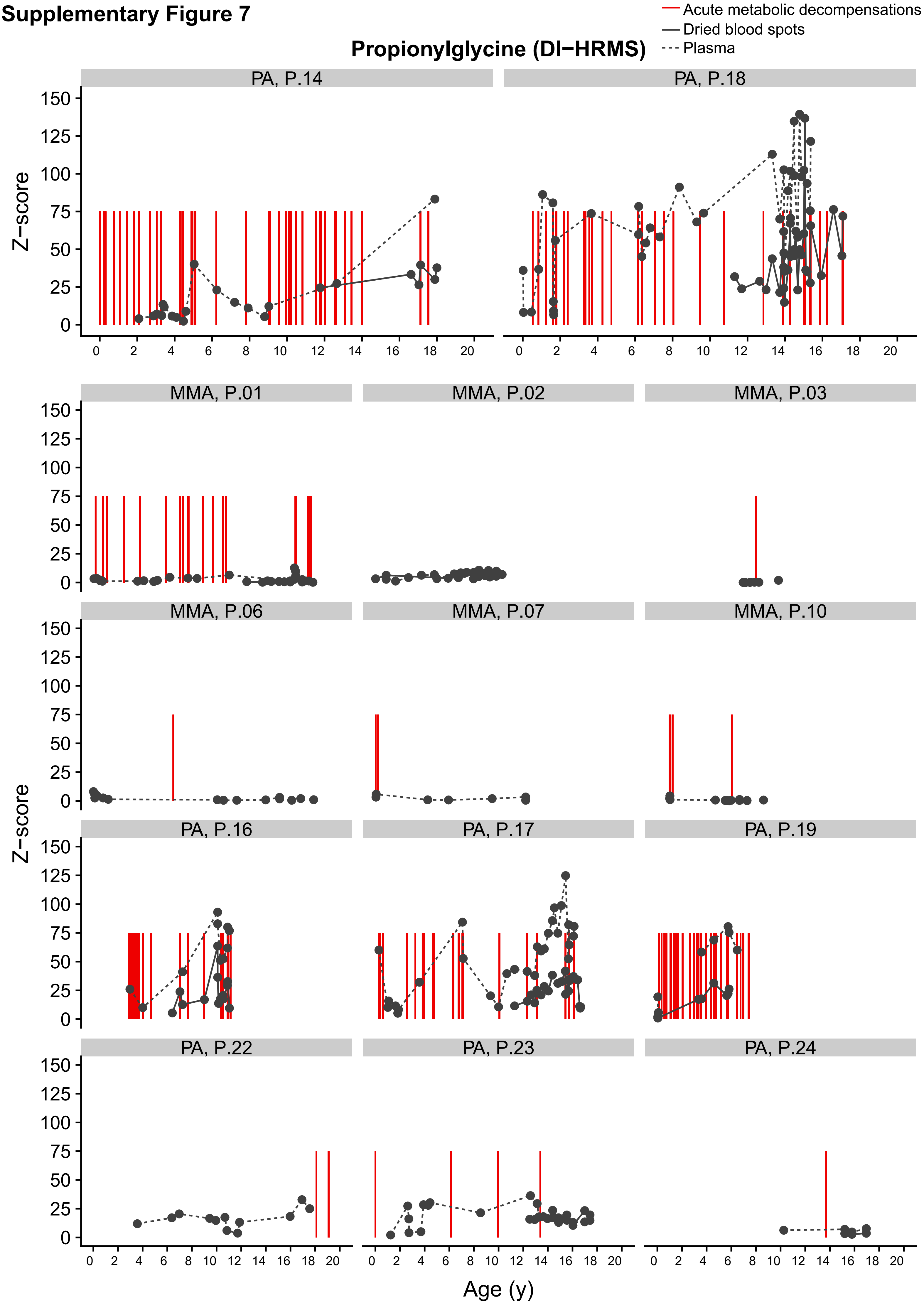

Supplementary Figure 8

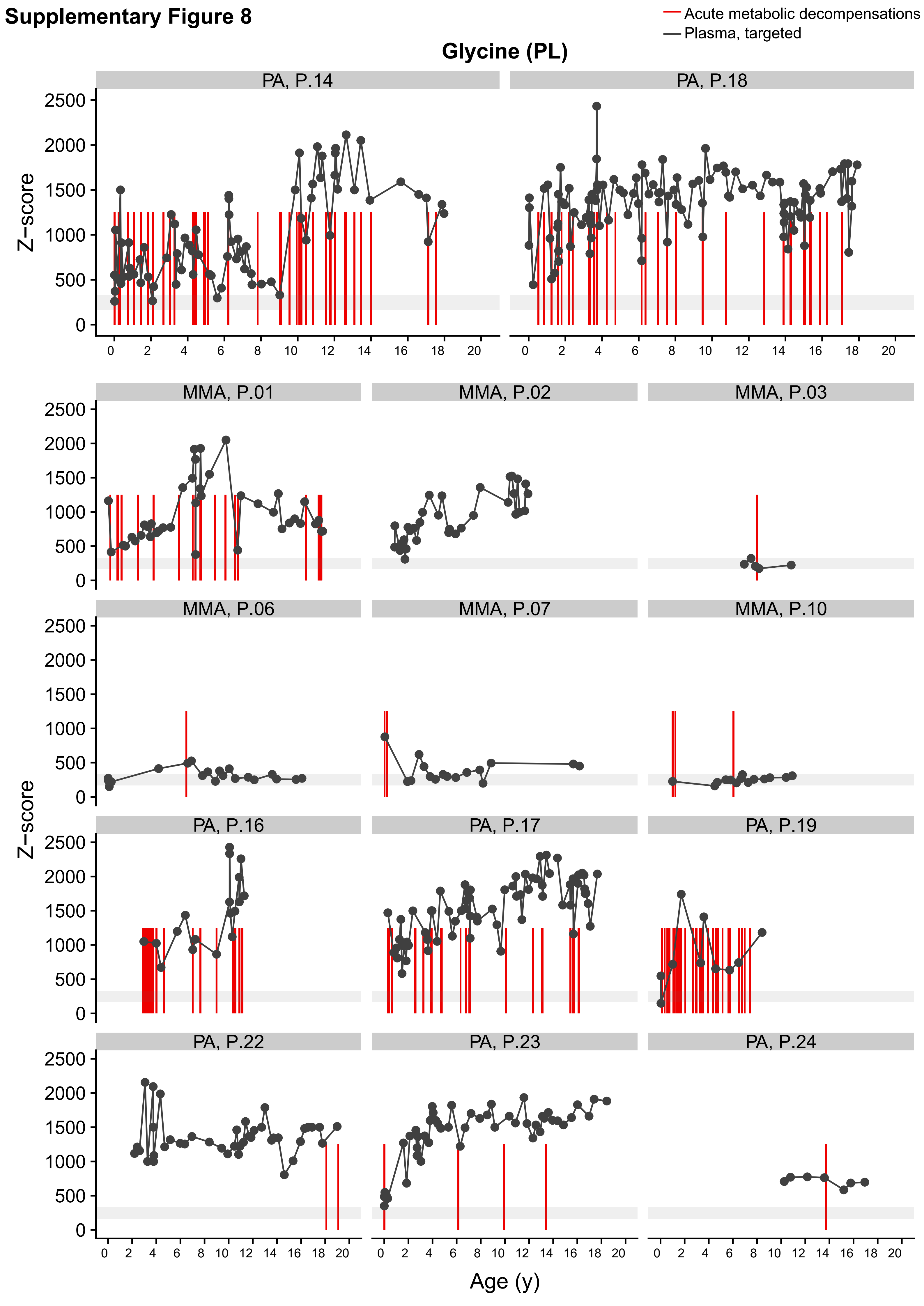

Supplementary Figure 9

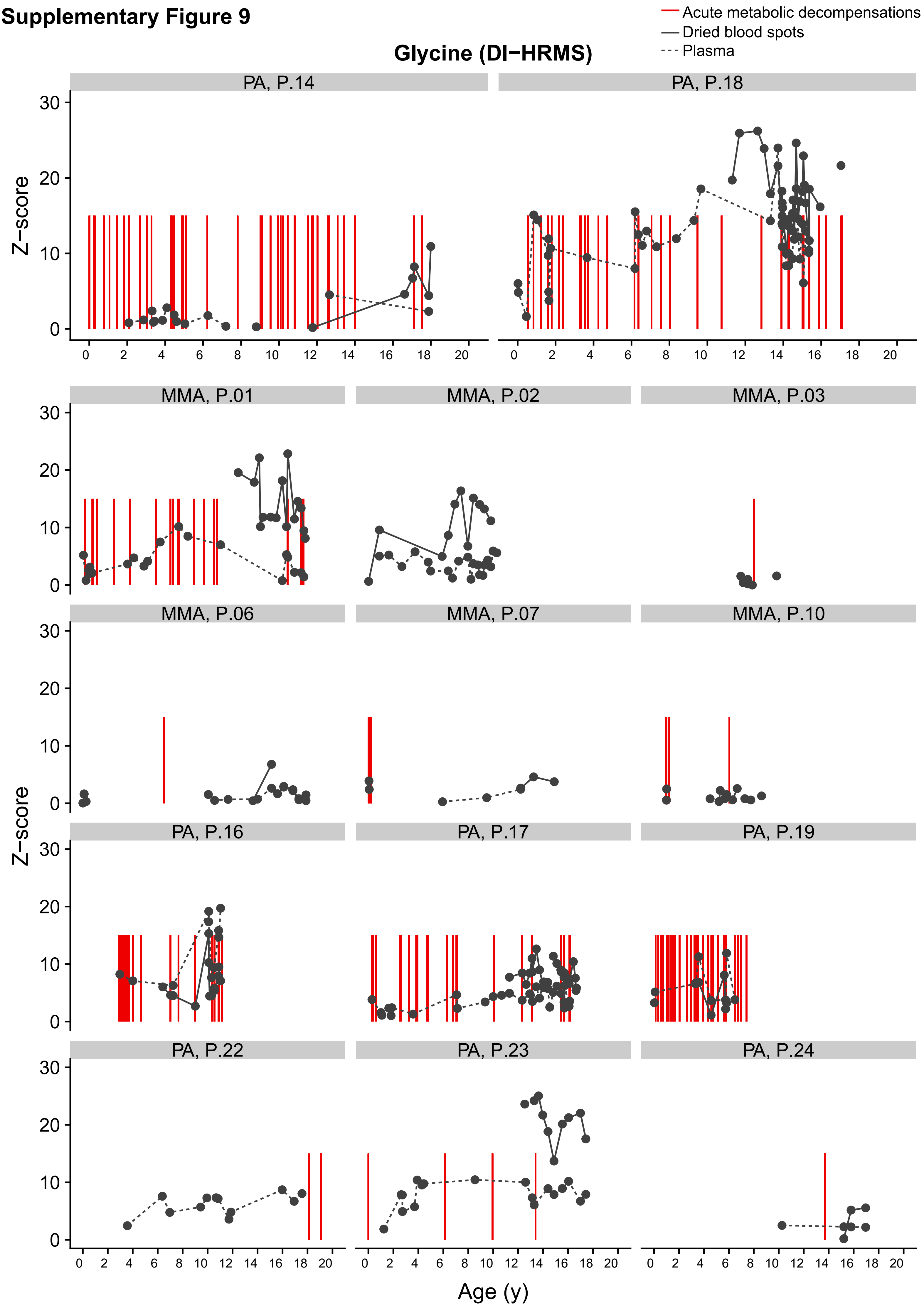

Supplementary Figure 10

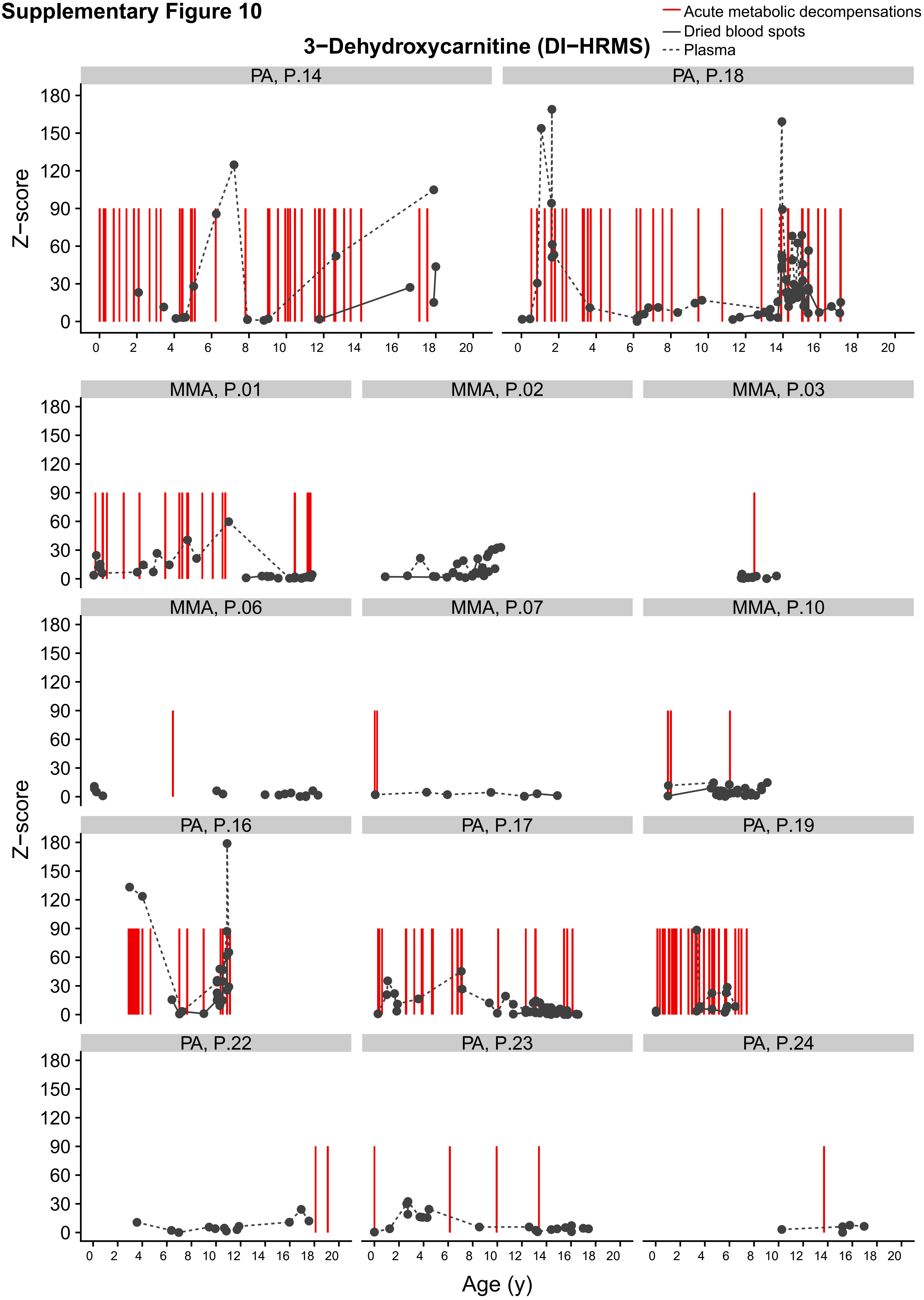

Supplementary Figure 11

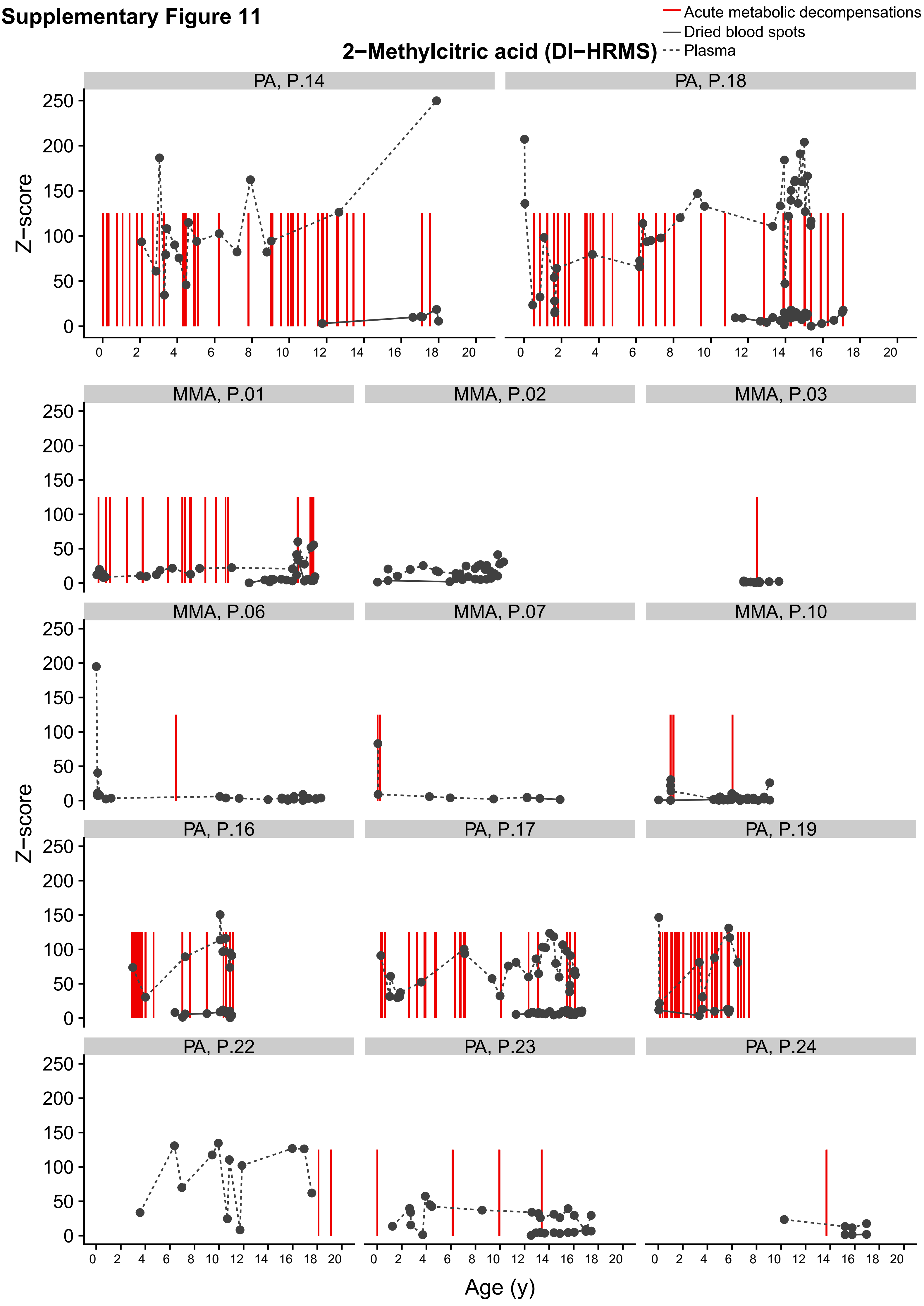

Supplementary Figure 12

PLS-DA, VIP-scores and heatmap for MMA samples versus control samples

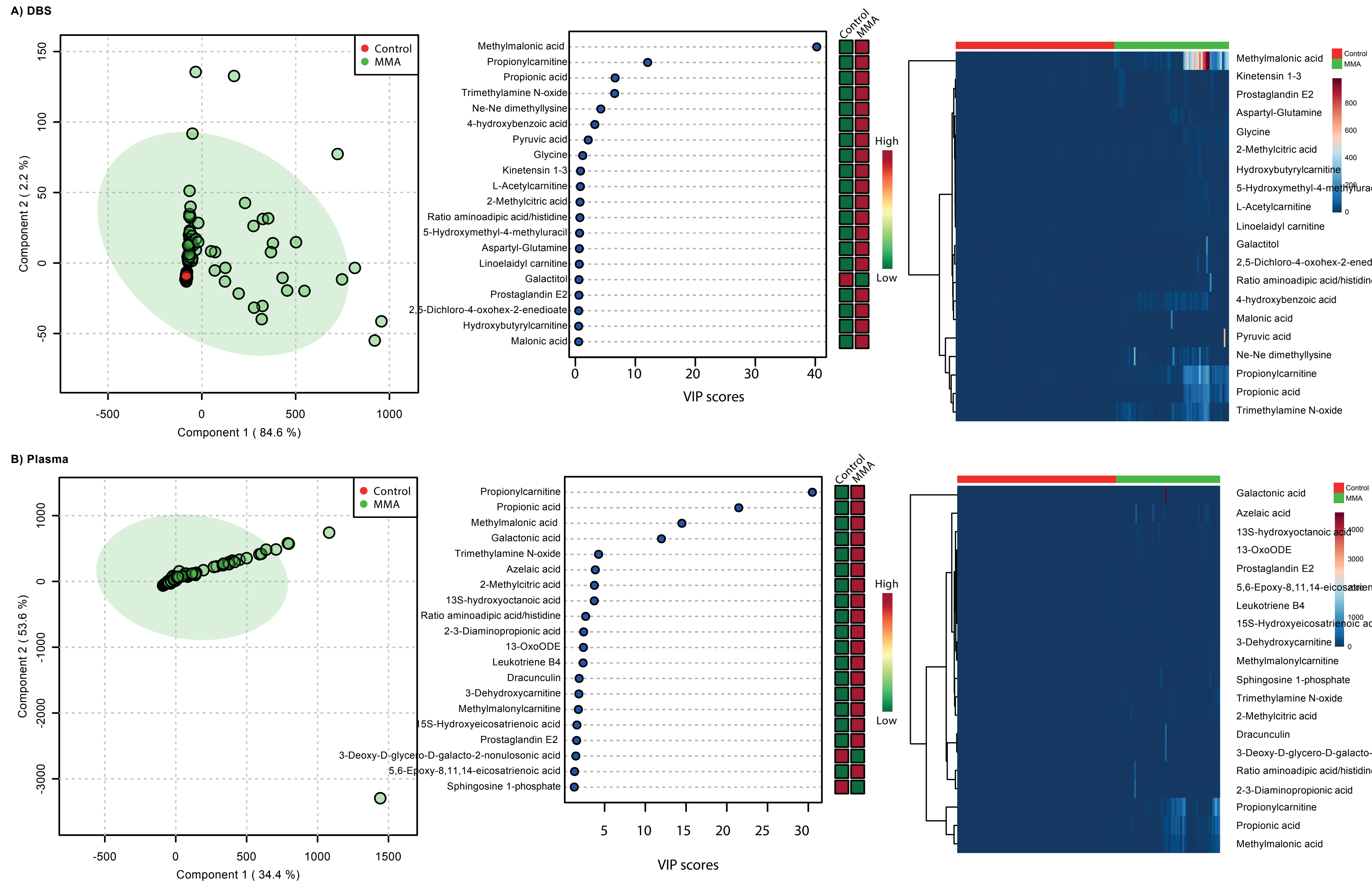

Supplementary Figure 13

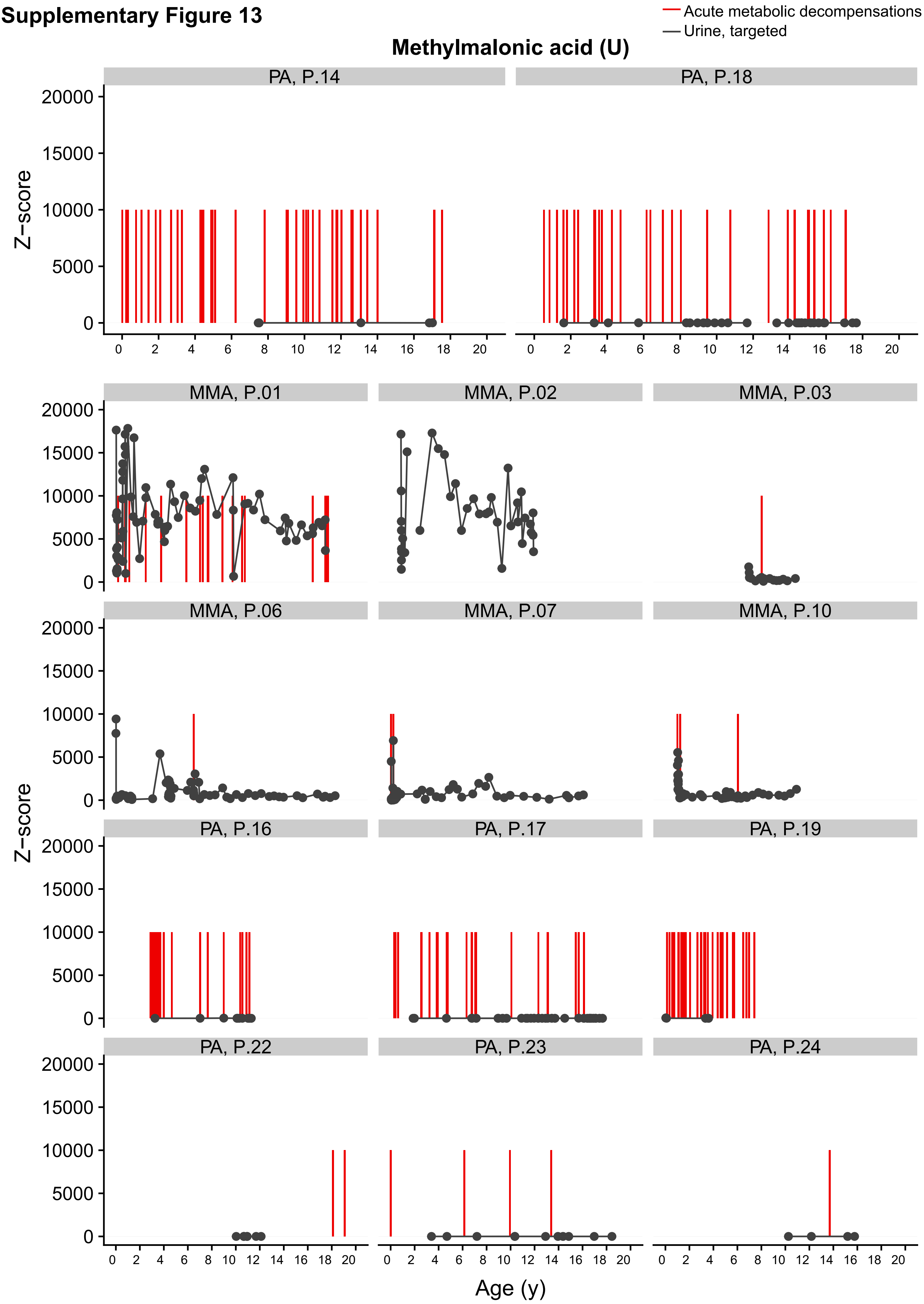

Supplementary Figure 14

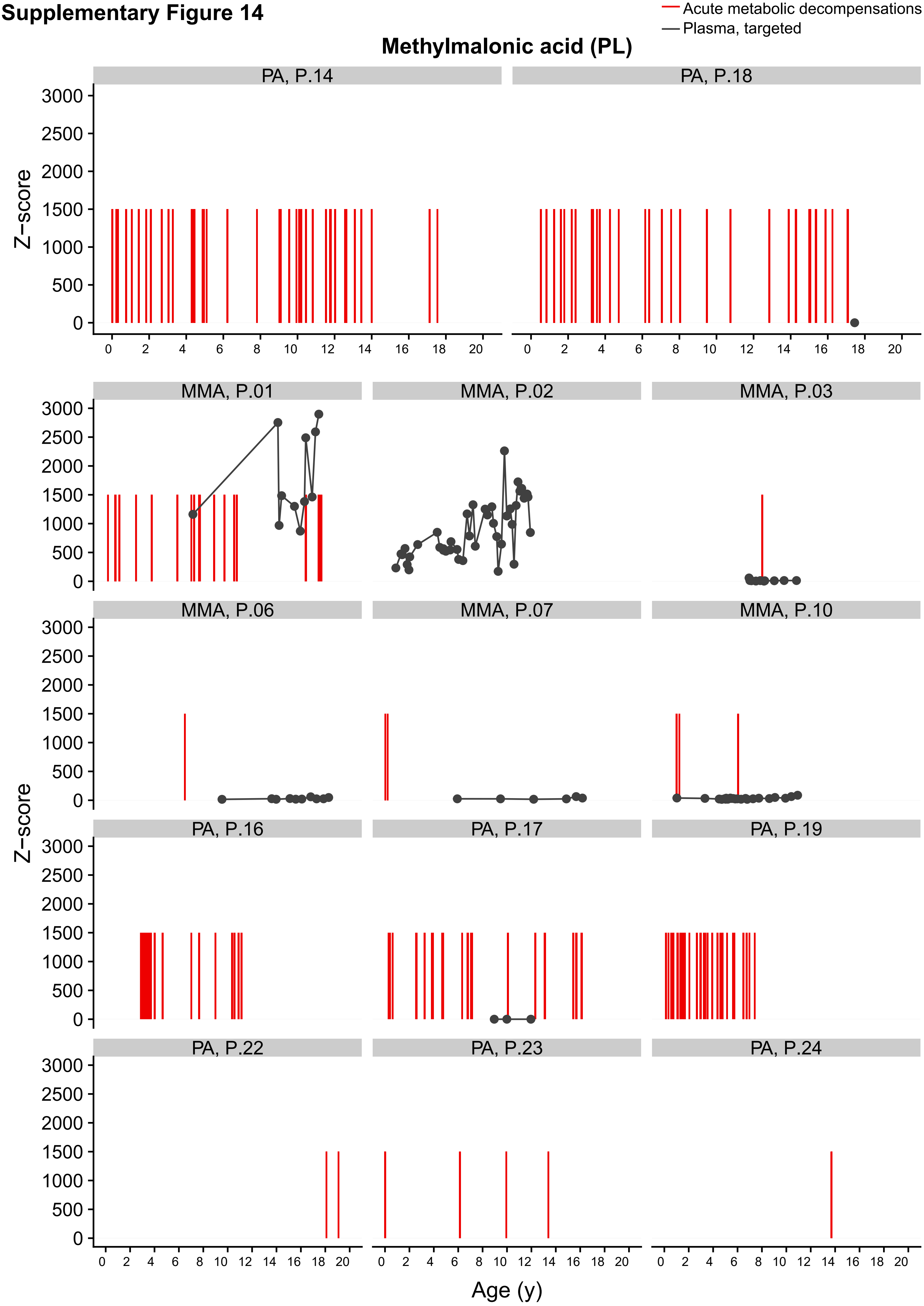

Supplementary Figure 15

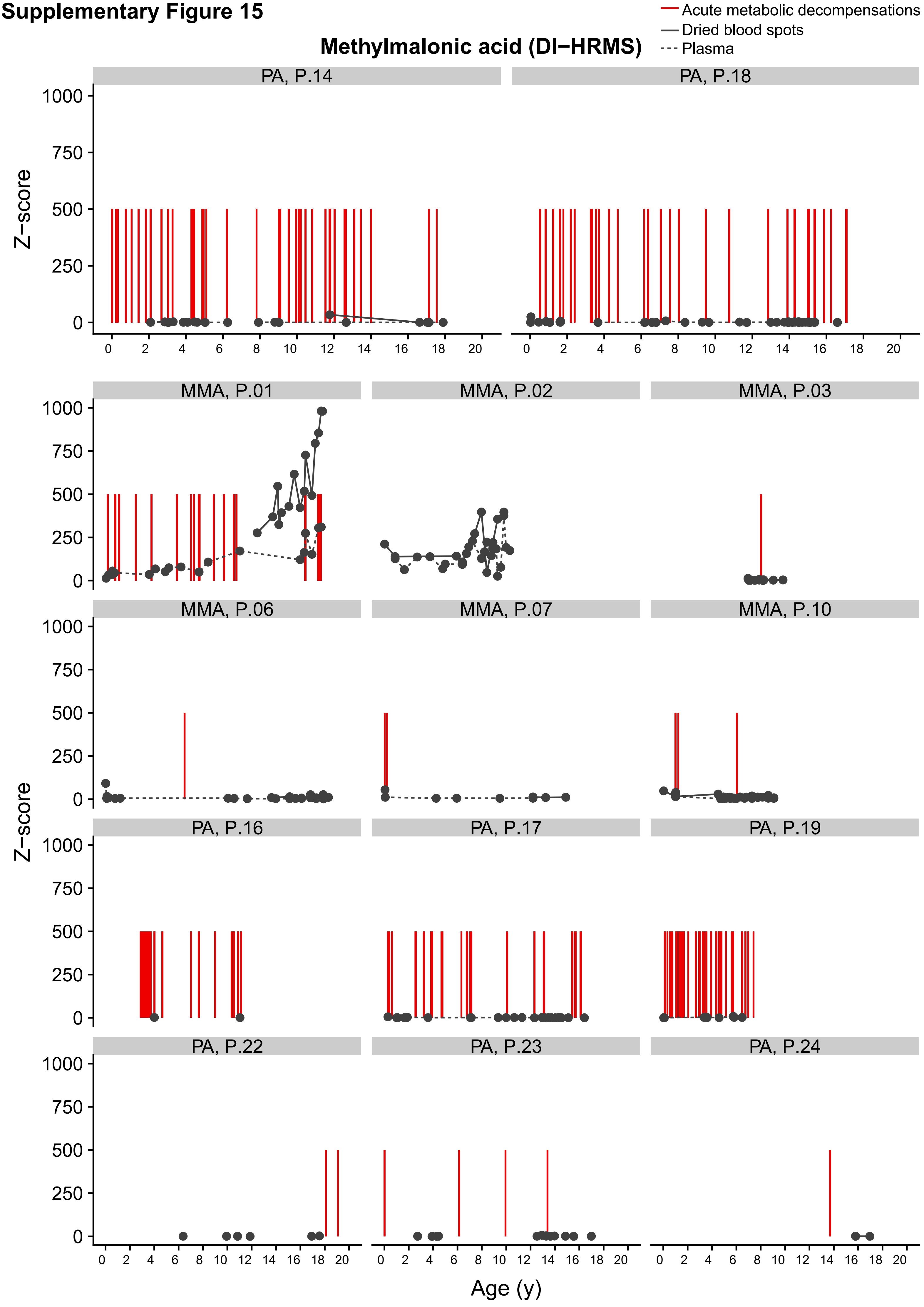

Supplementary Figure 16

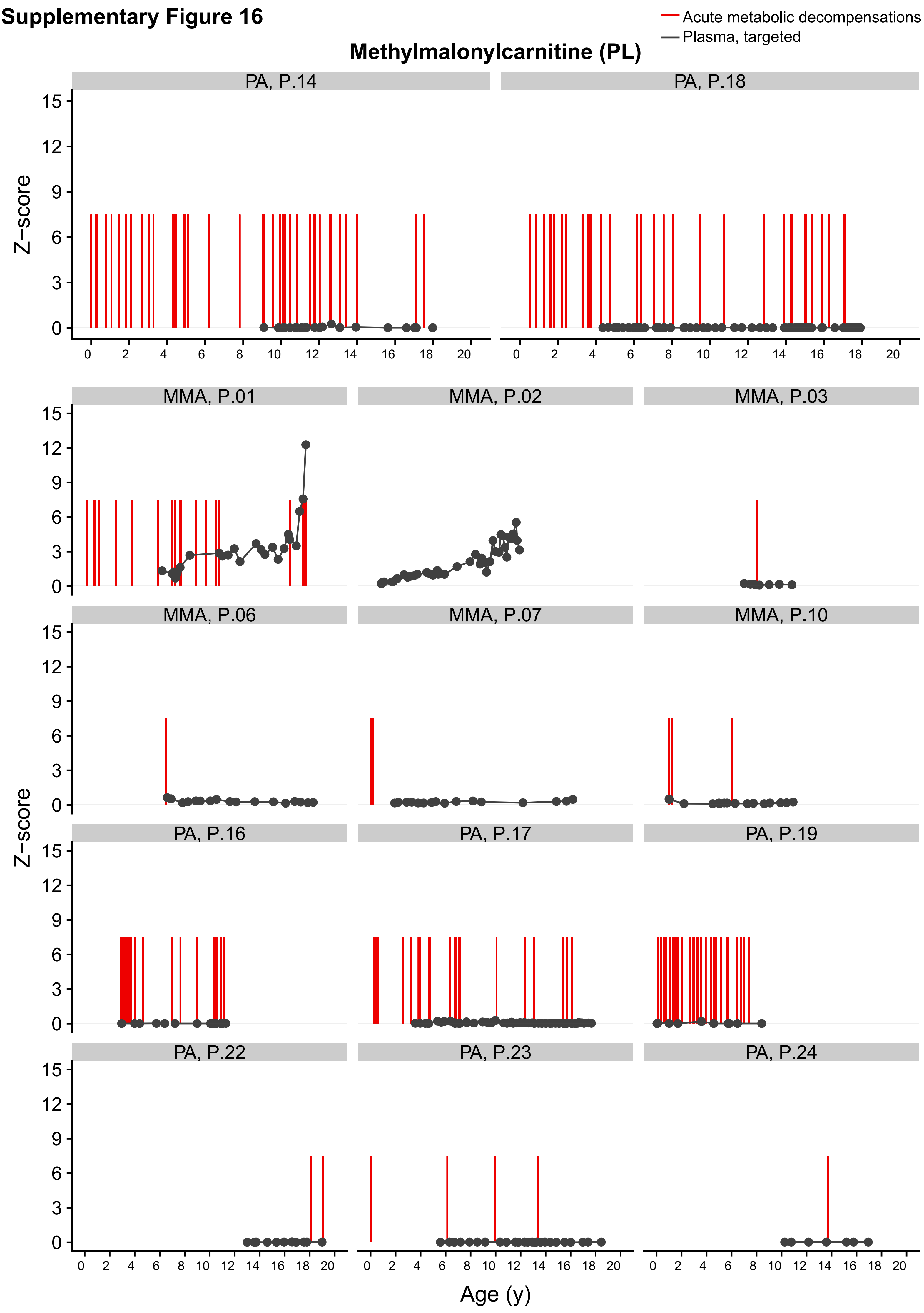

Supplementary Figure 17

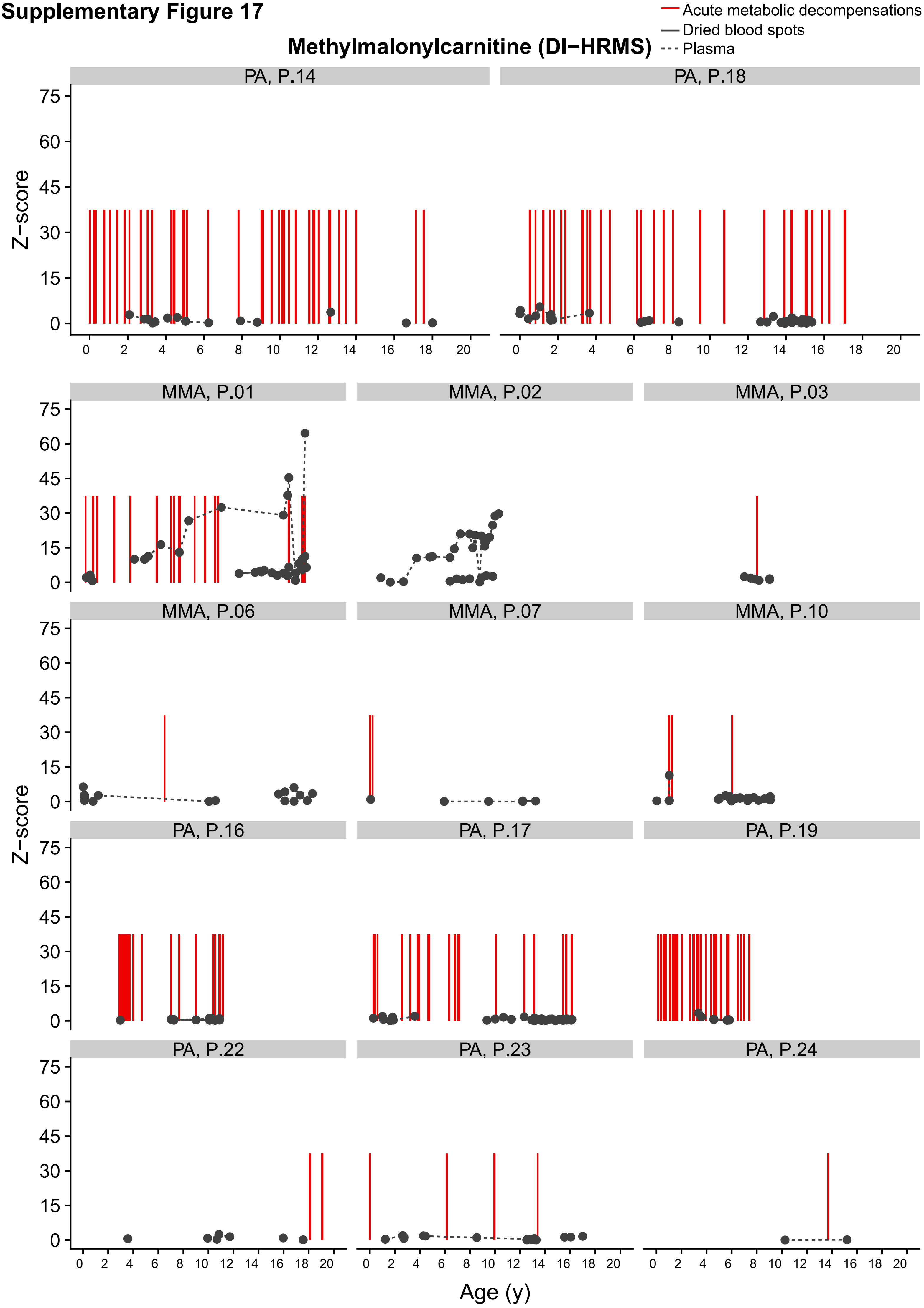

Supplementary Figure 18

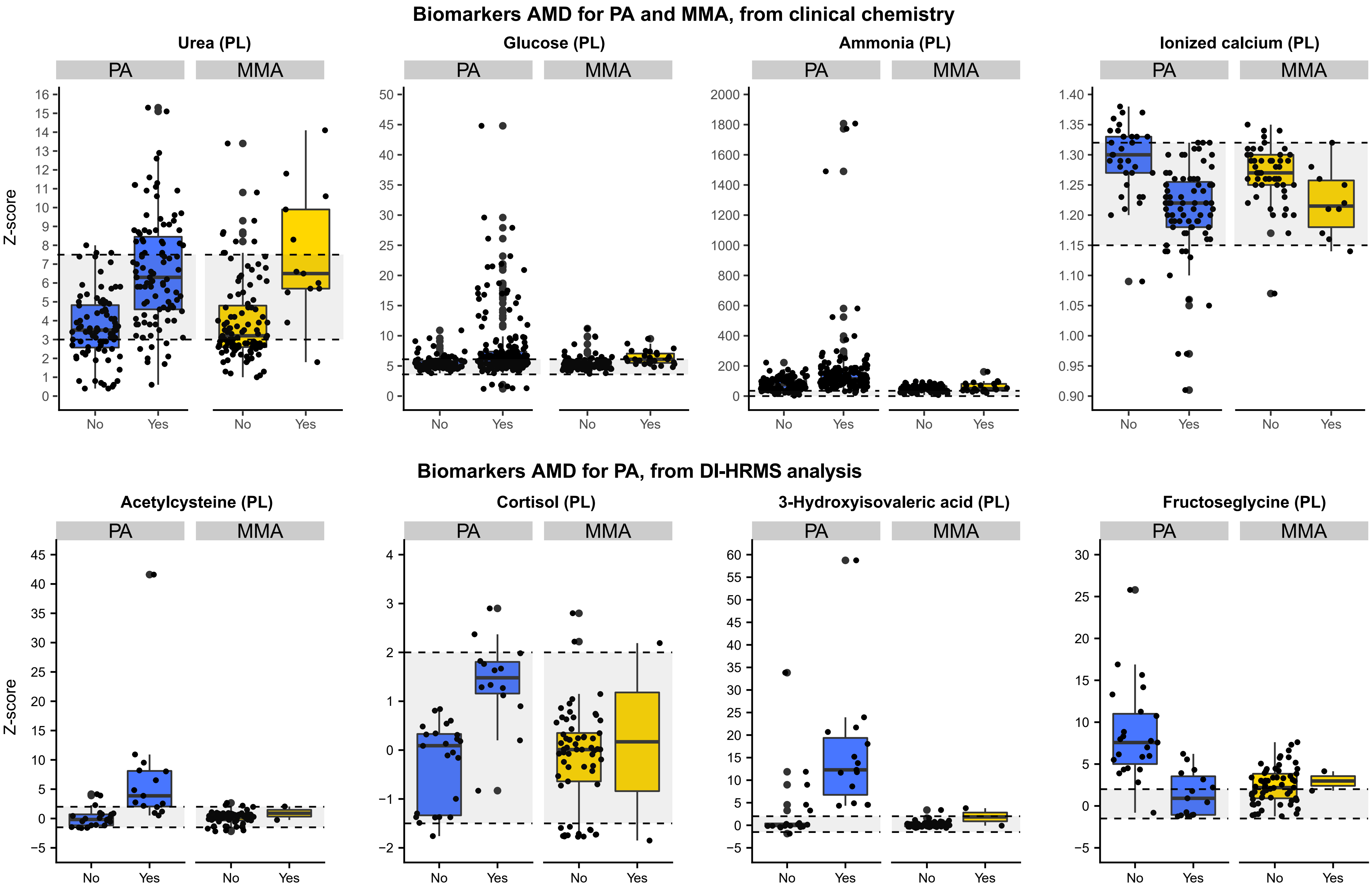

Supplementary Figure 19

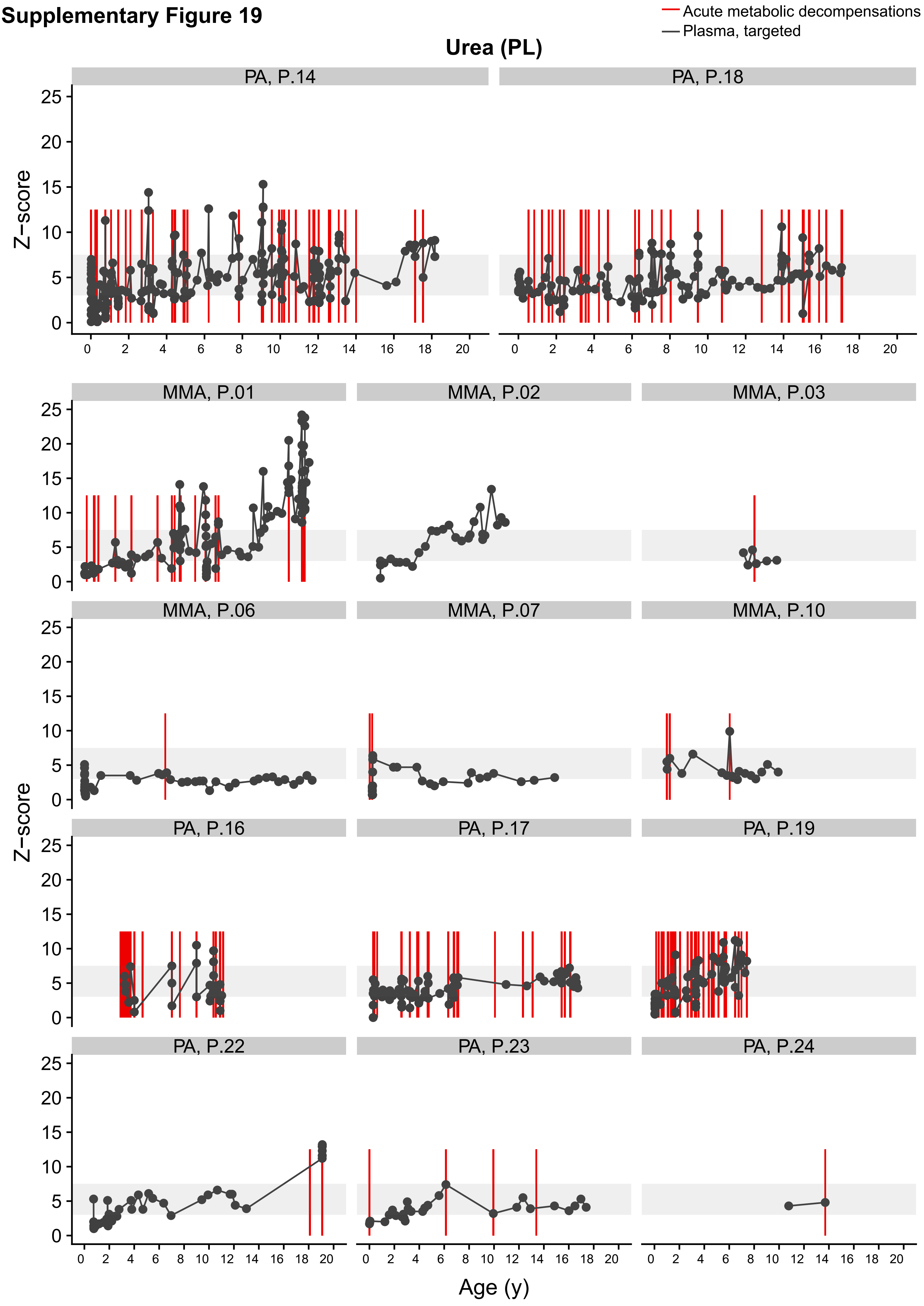

Supplementary Figure 20

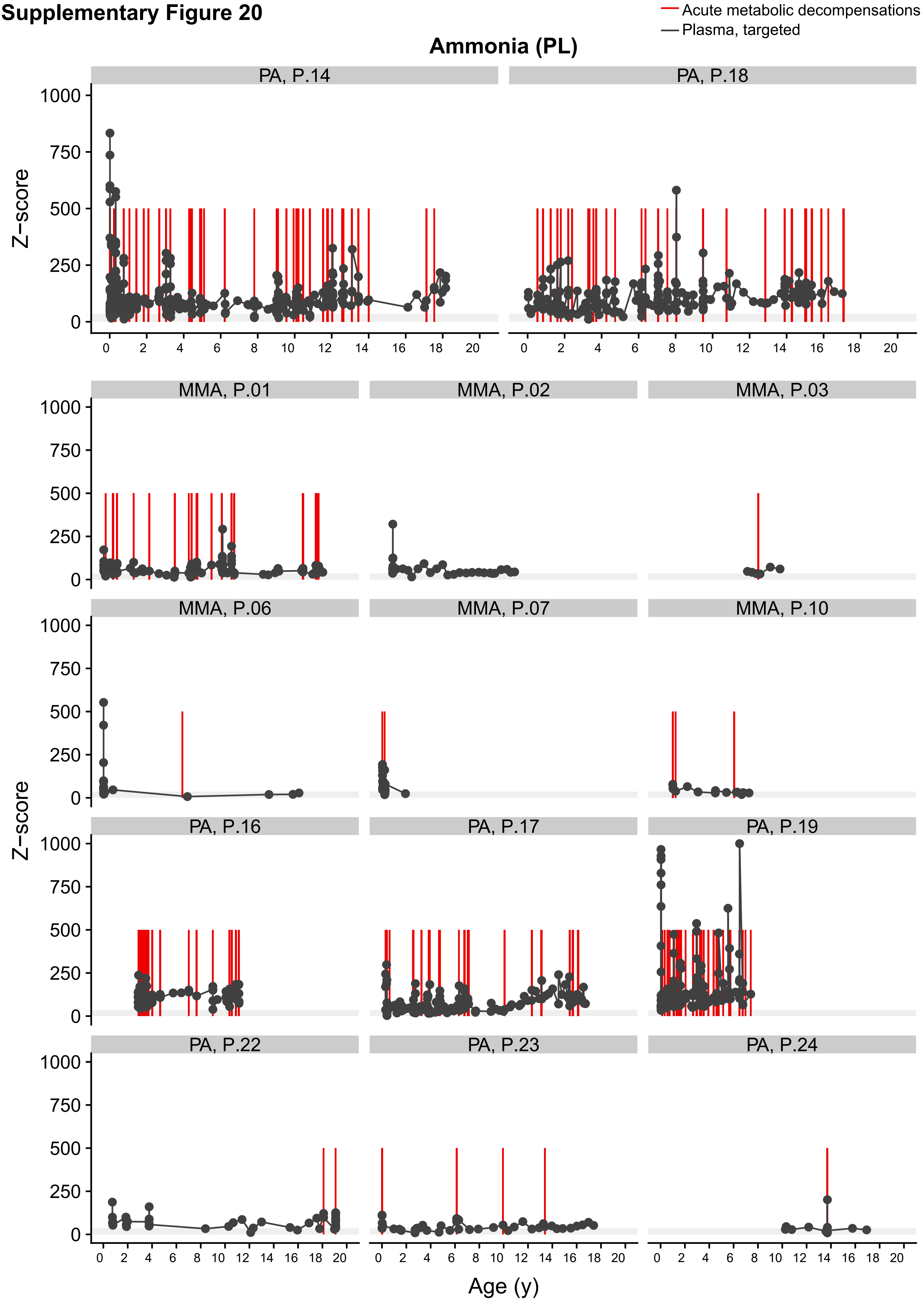

Supplementary Figure 21

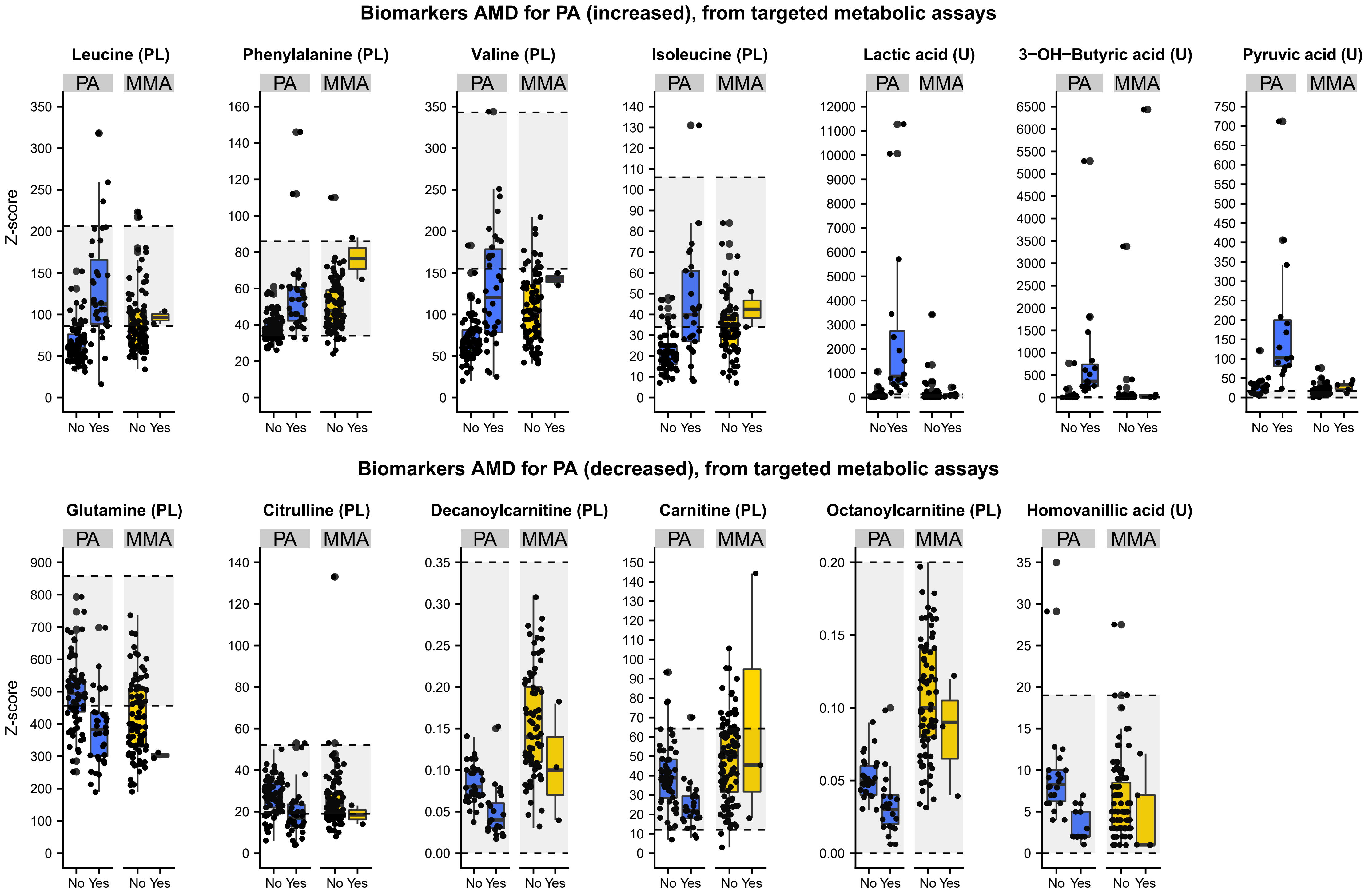

Supplementary Figure 22

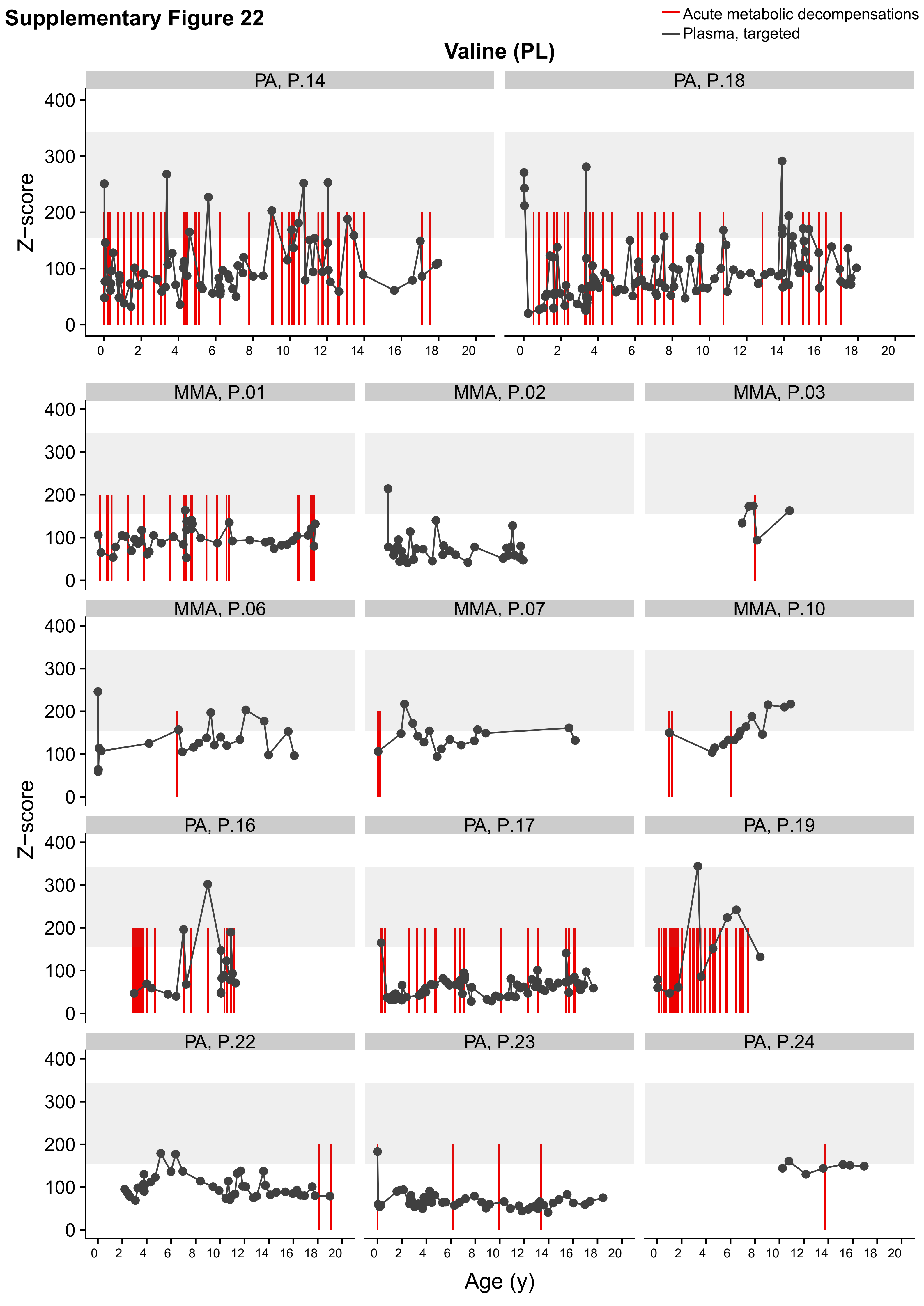

Supplementary Figure 23

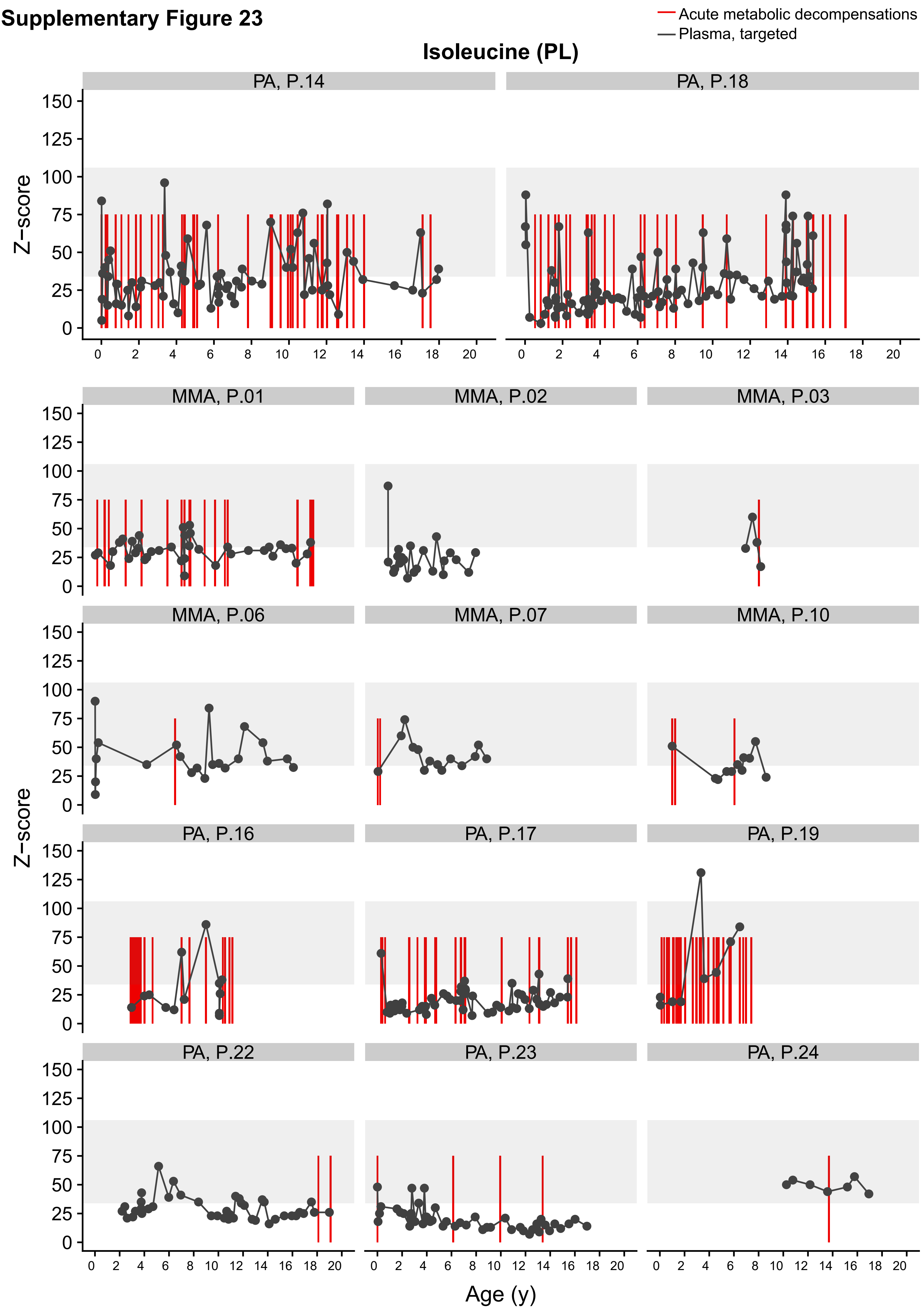

Supplementary Figure 24

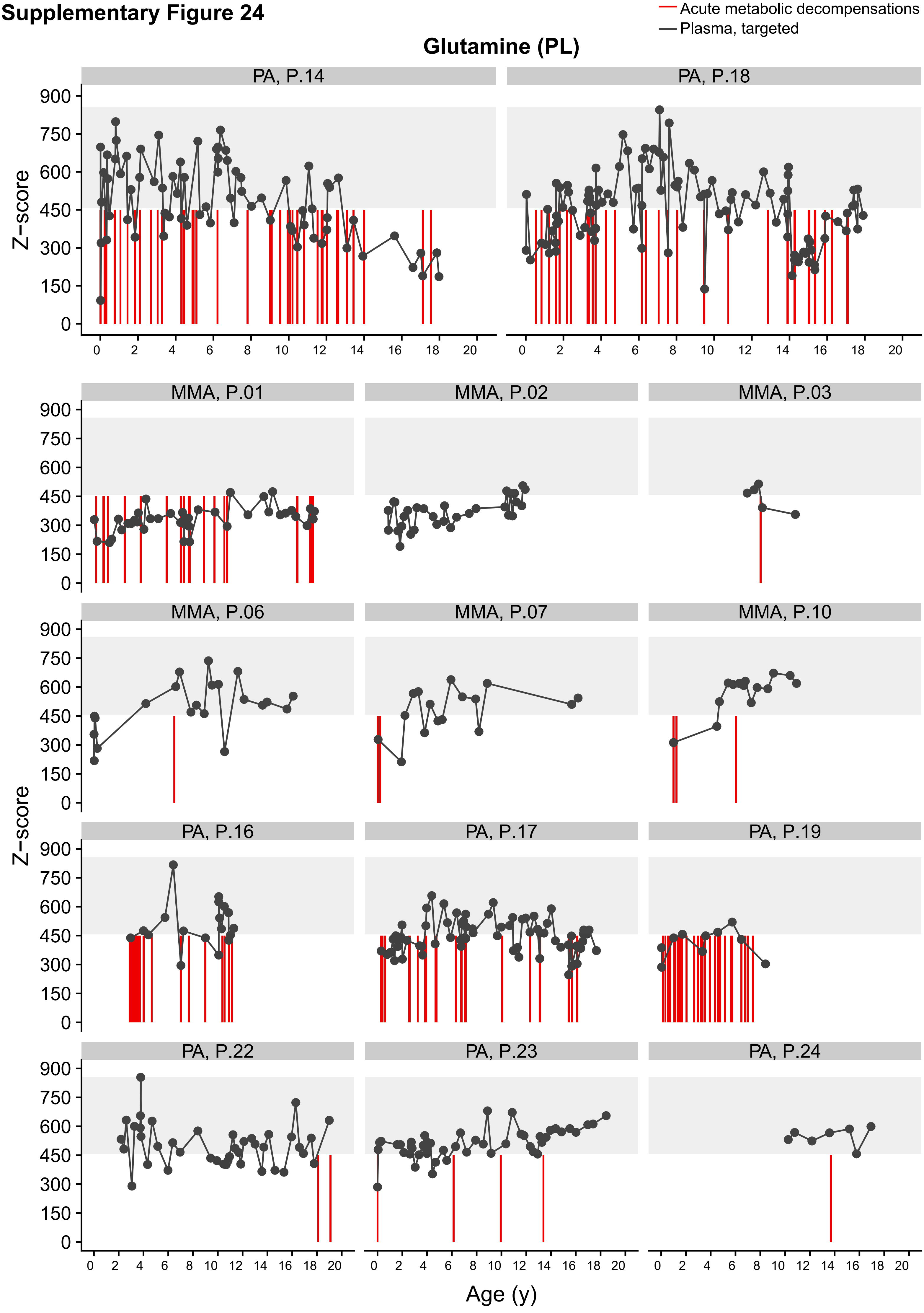

Supplementary Figure 25

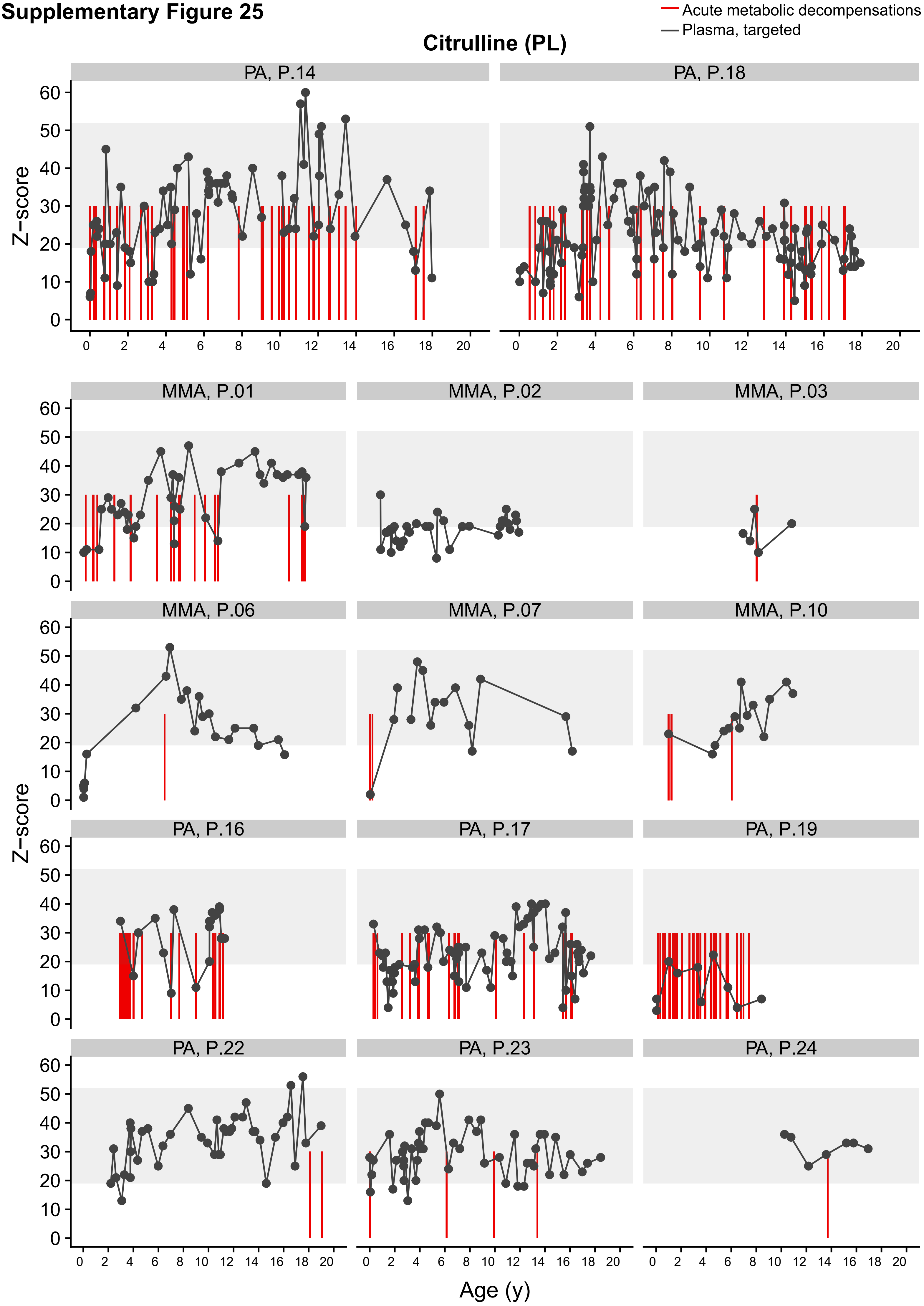

Supplementary Figure 26

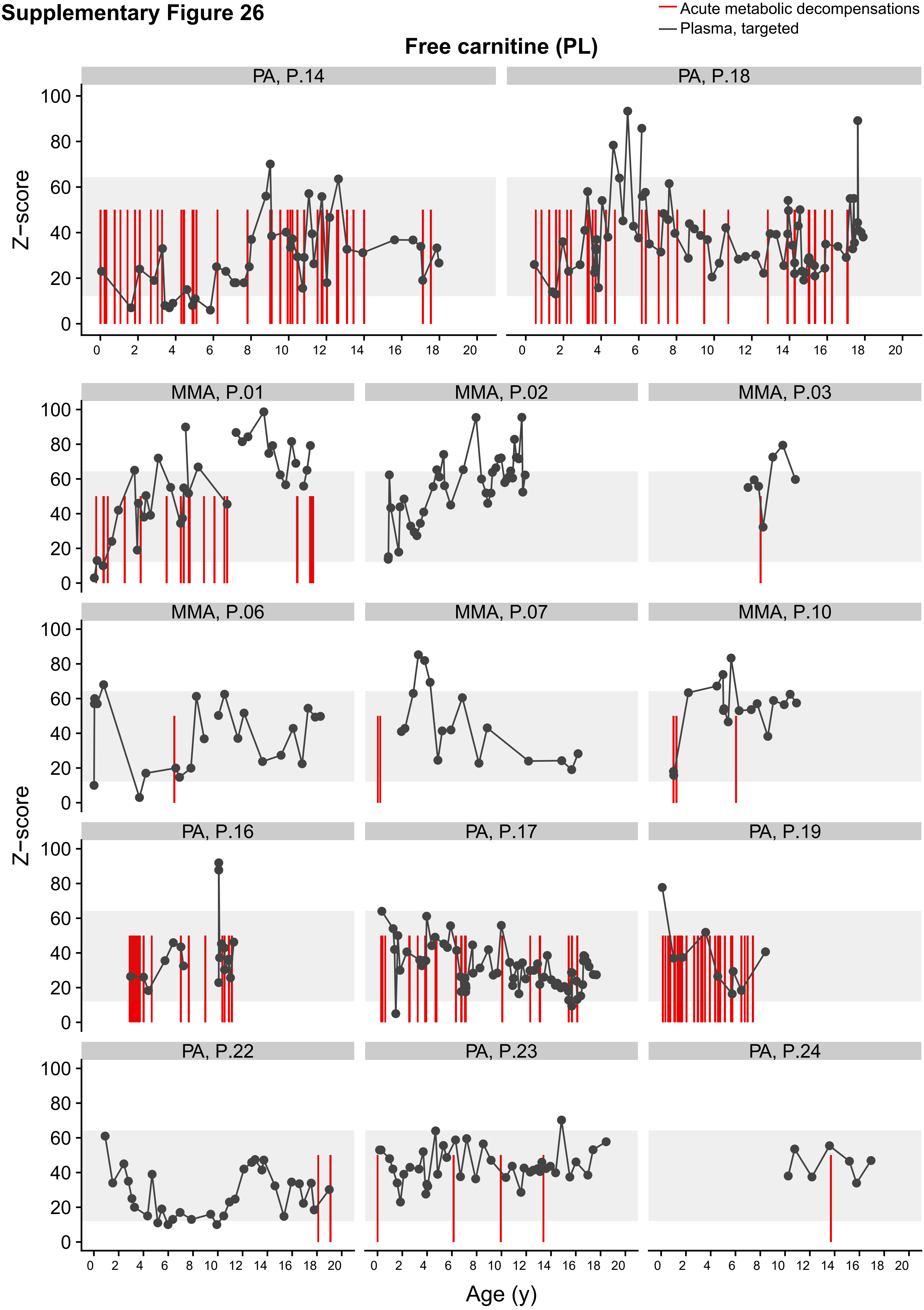

Supplementary Figure 27

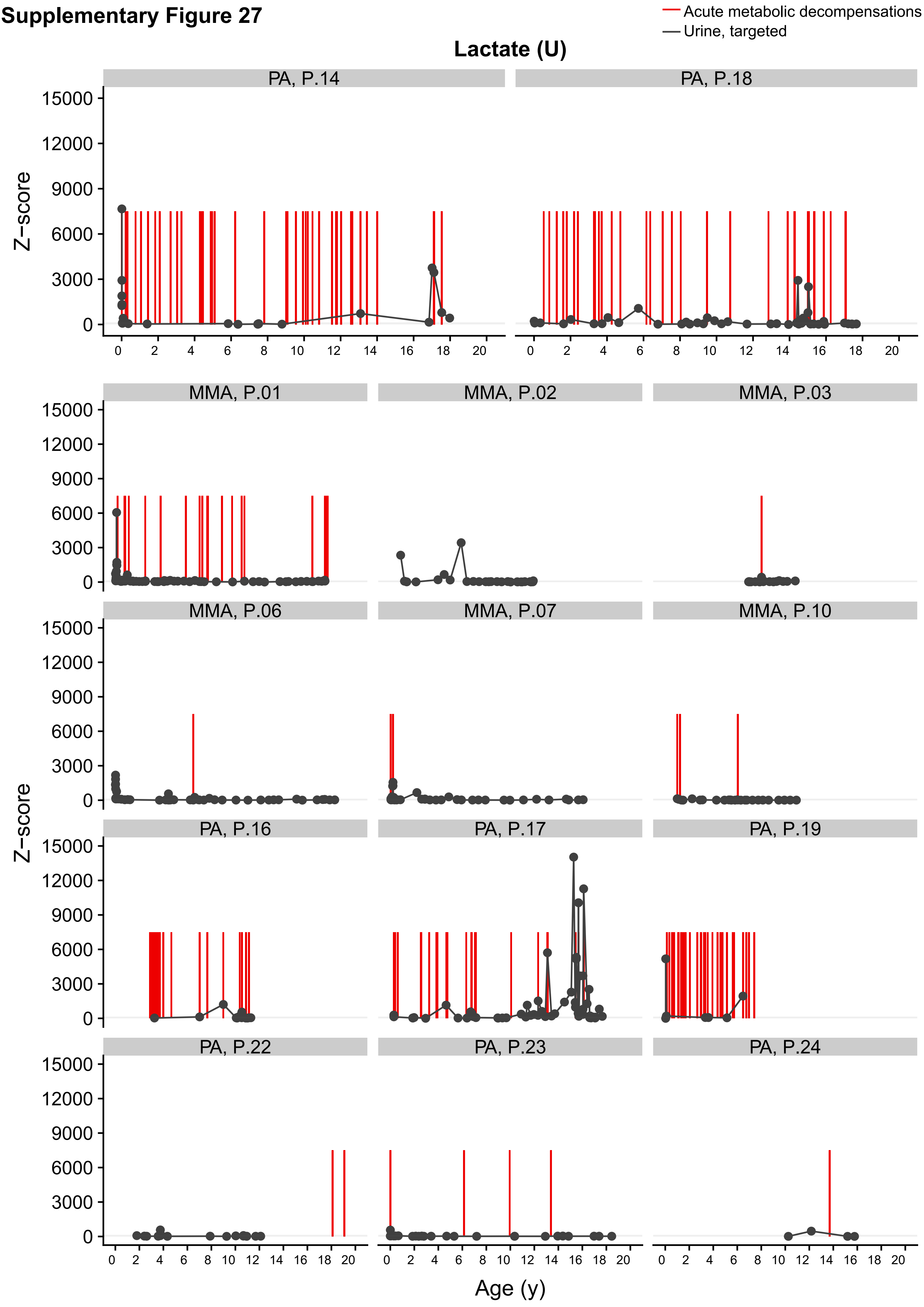

Supplementary Figure 28

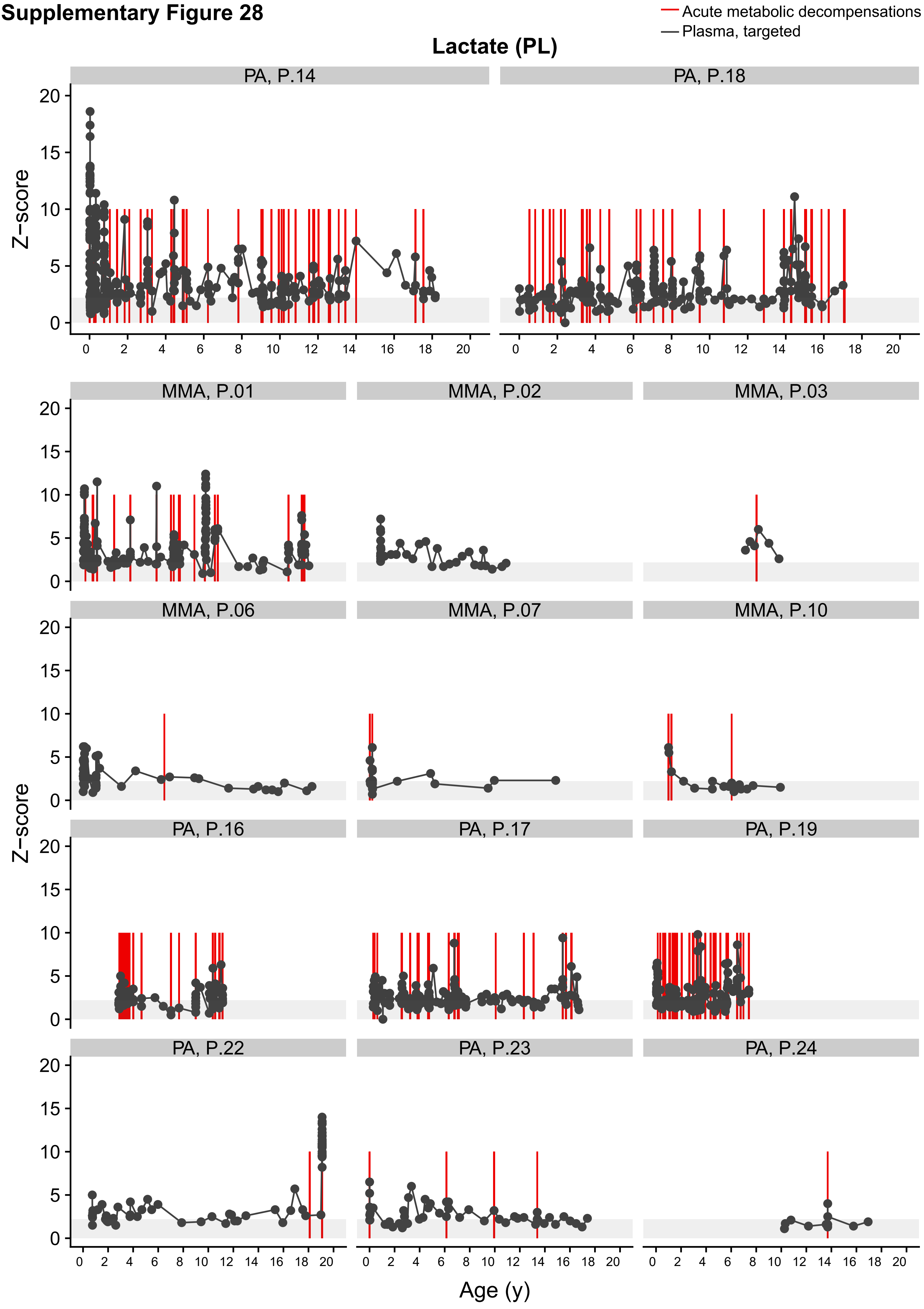

Supplementary Figure 29

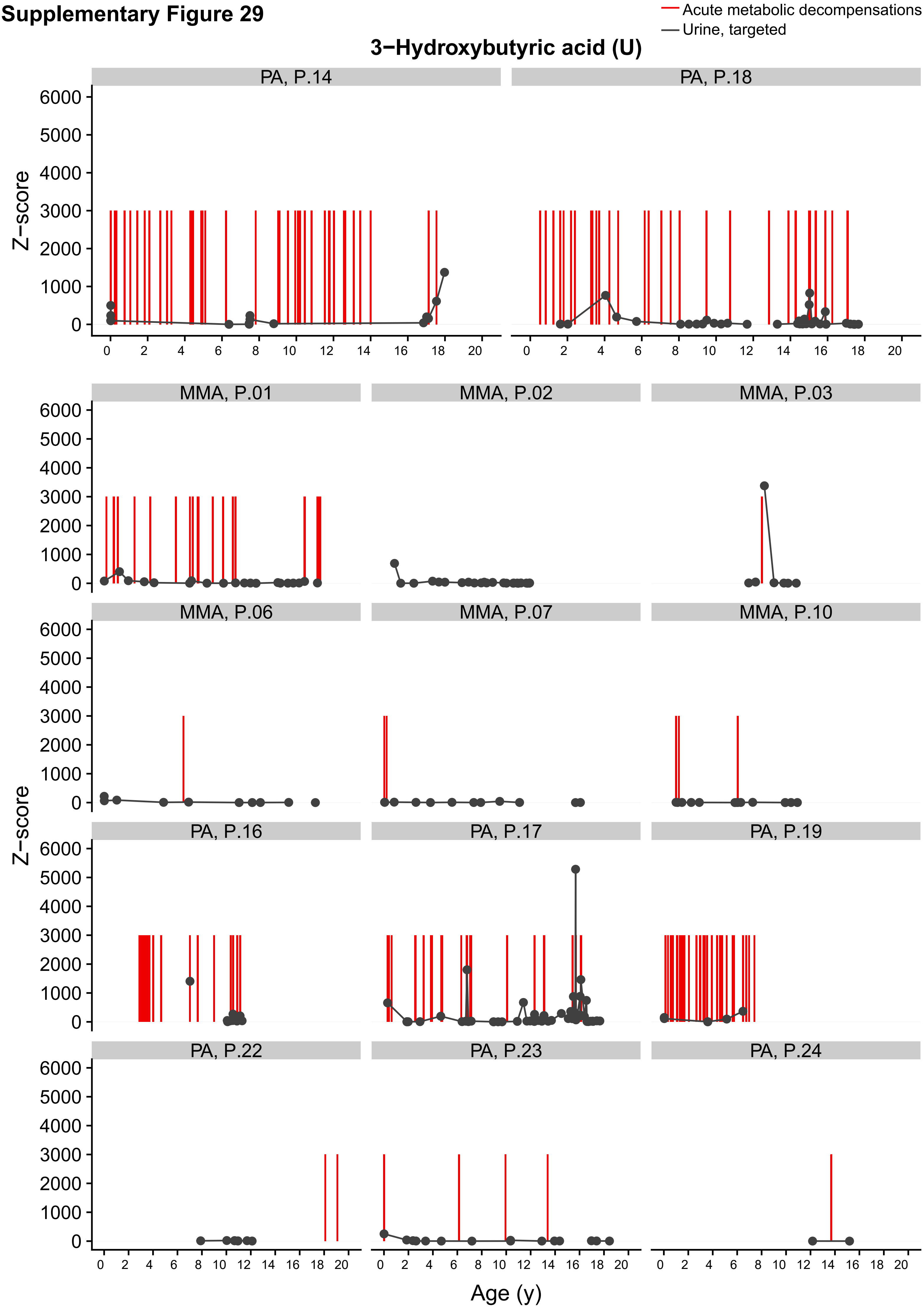

Supplementary Figure 30

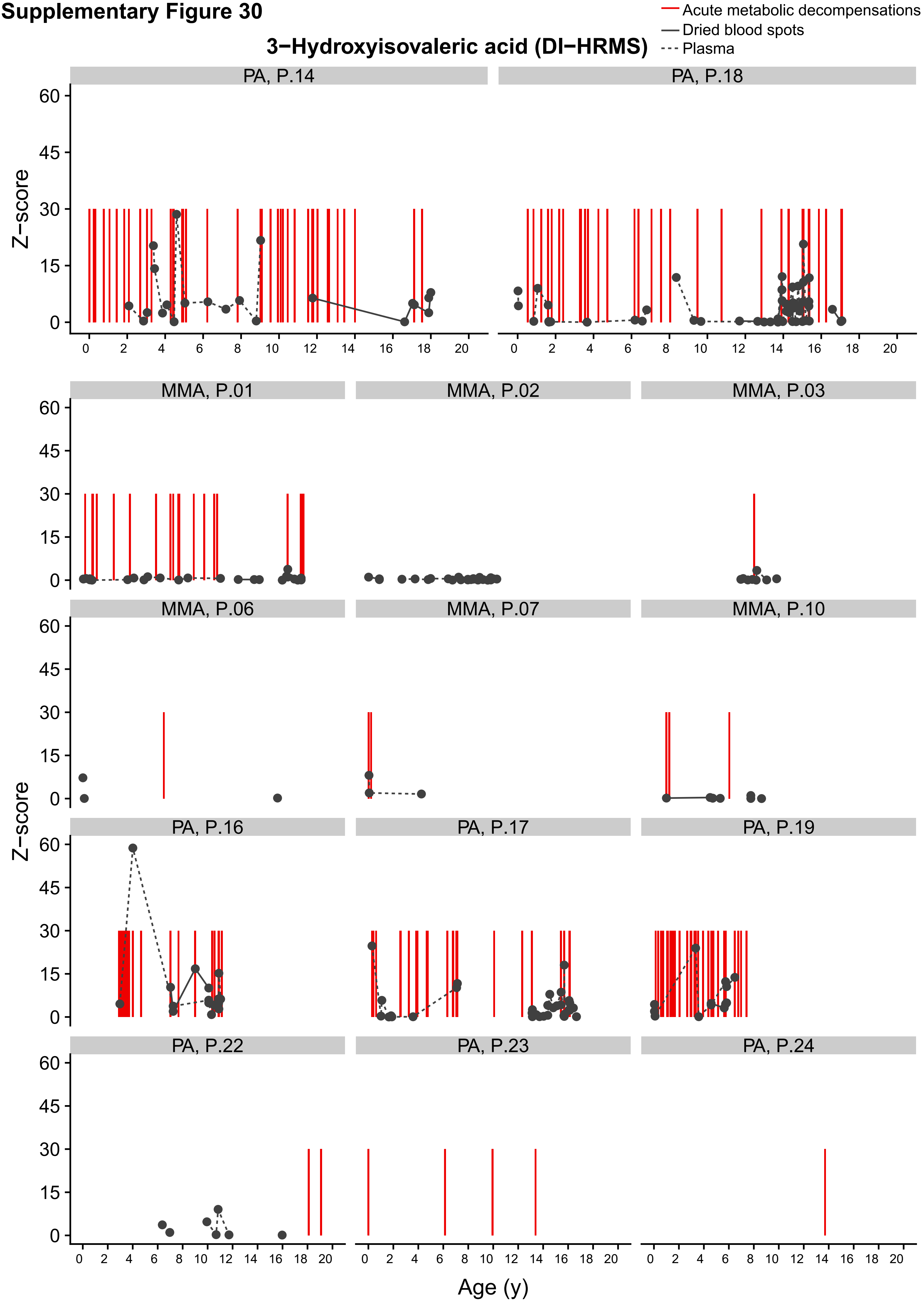

Supplementary Figure 31

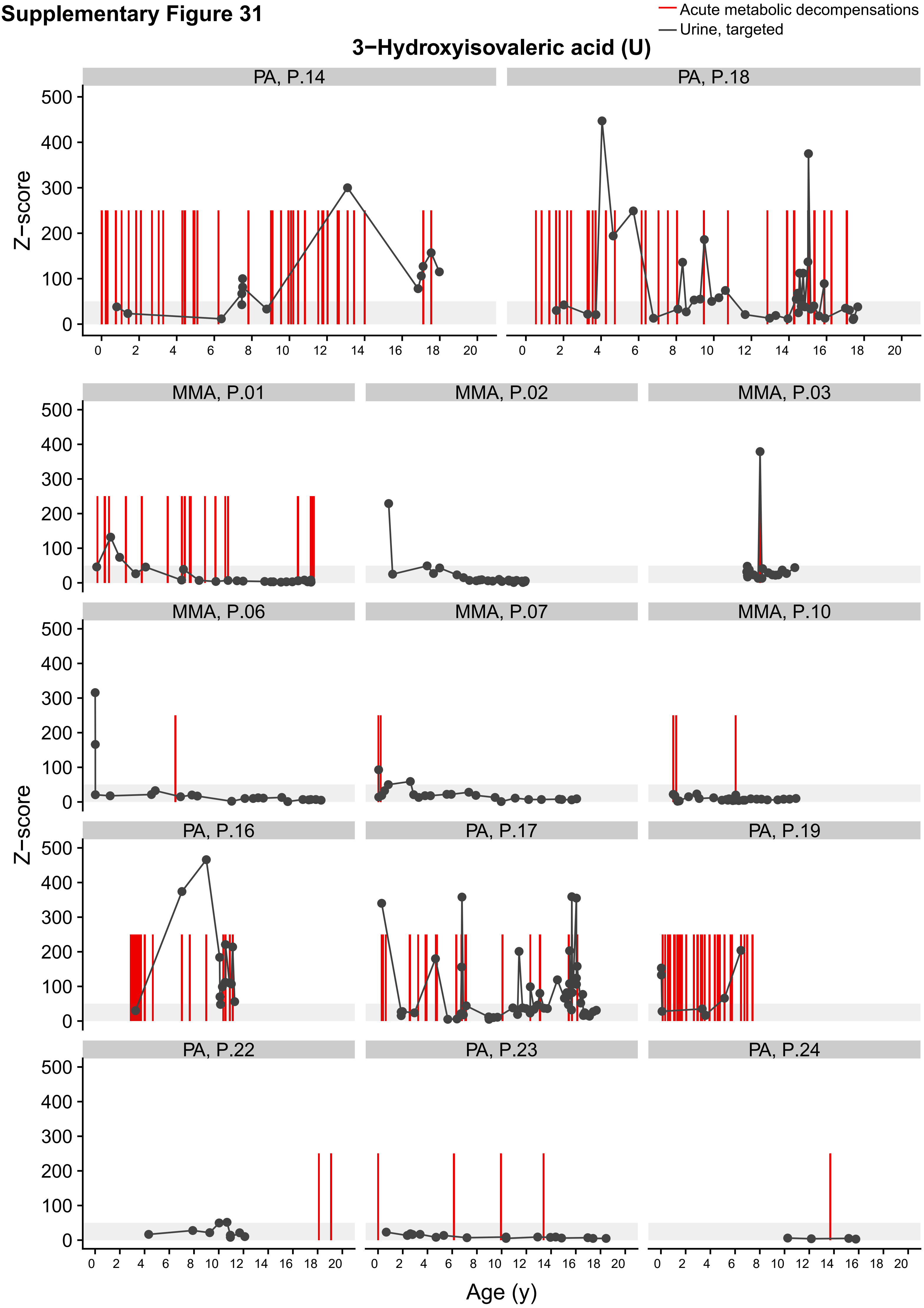

Supplementary Figure 32

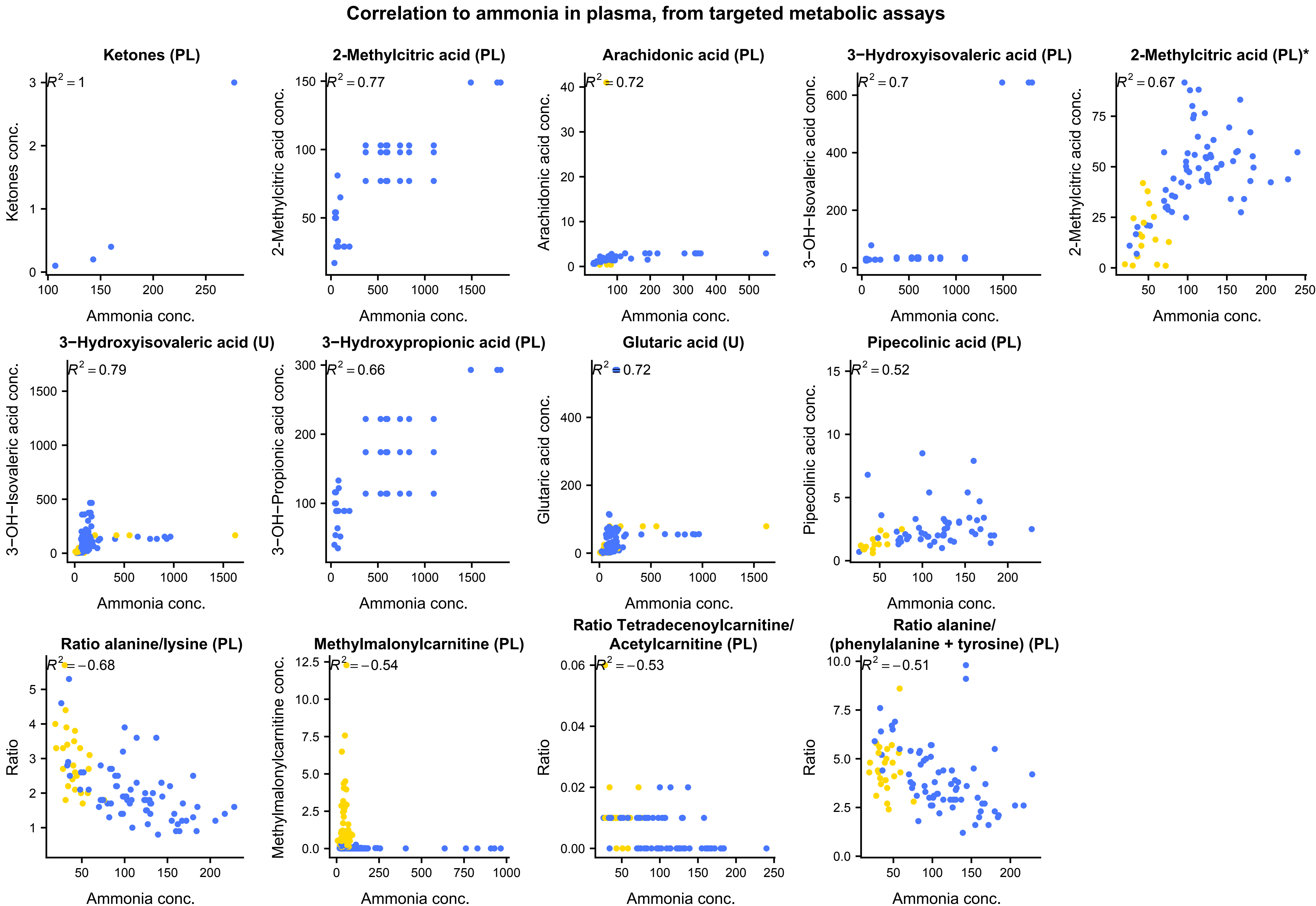

Supplementary Figure 33

Correlation to Ammonia in plasma, from DI−HRMS analysis

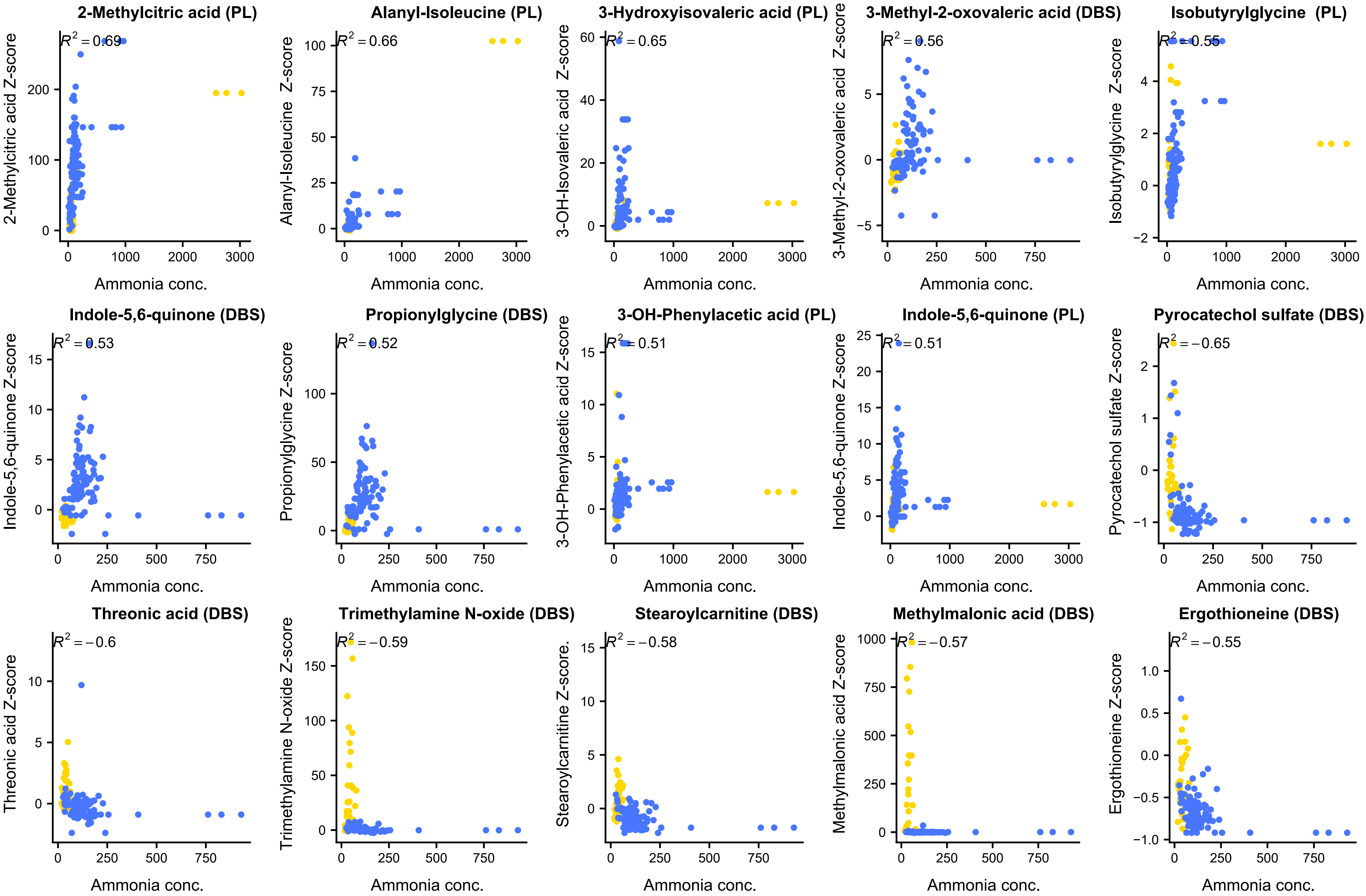

Supplementary Figure 34

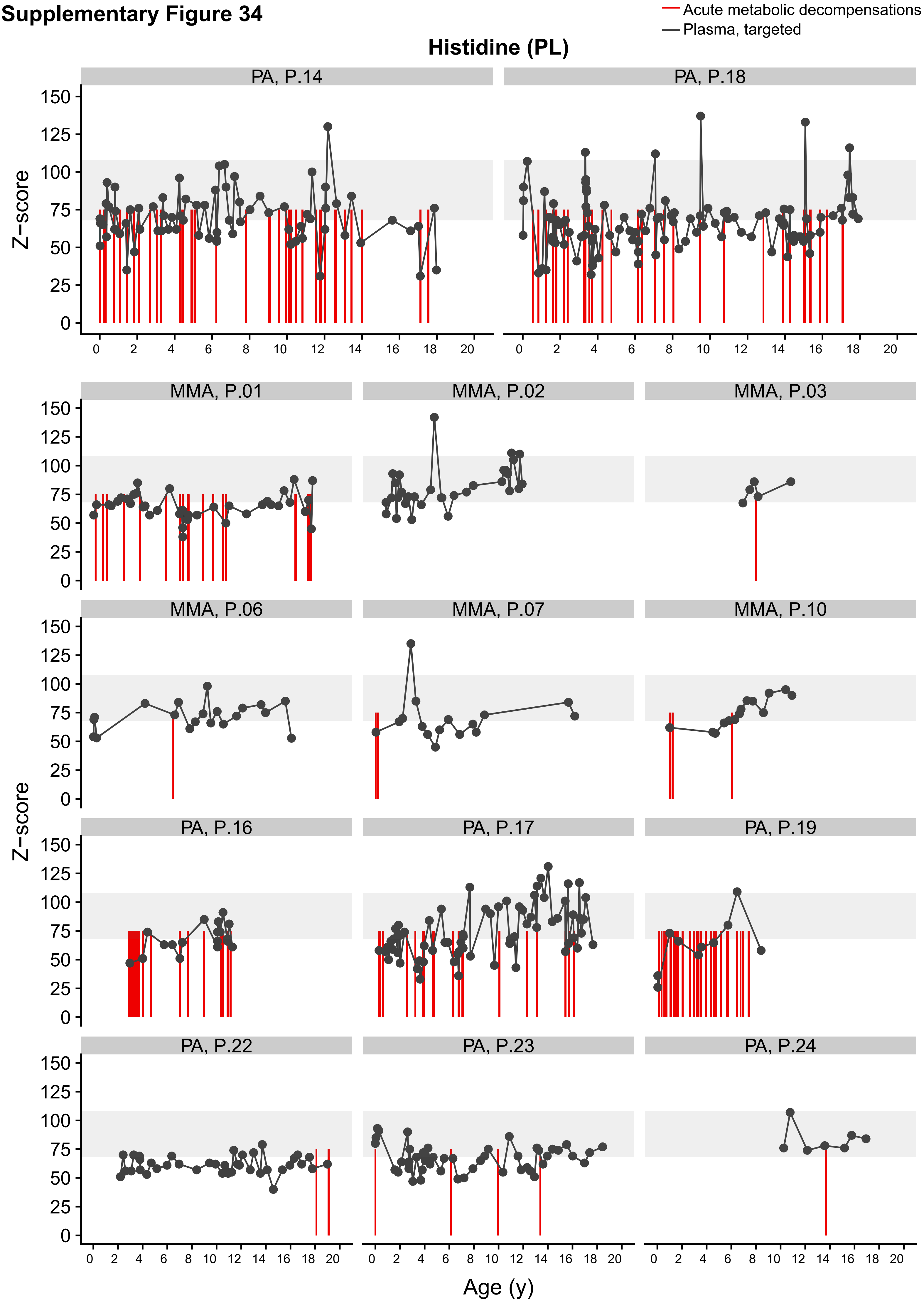

Supplementary Figure 35

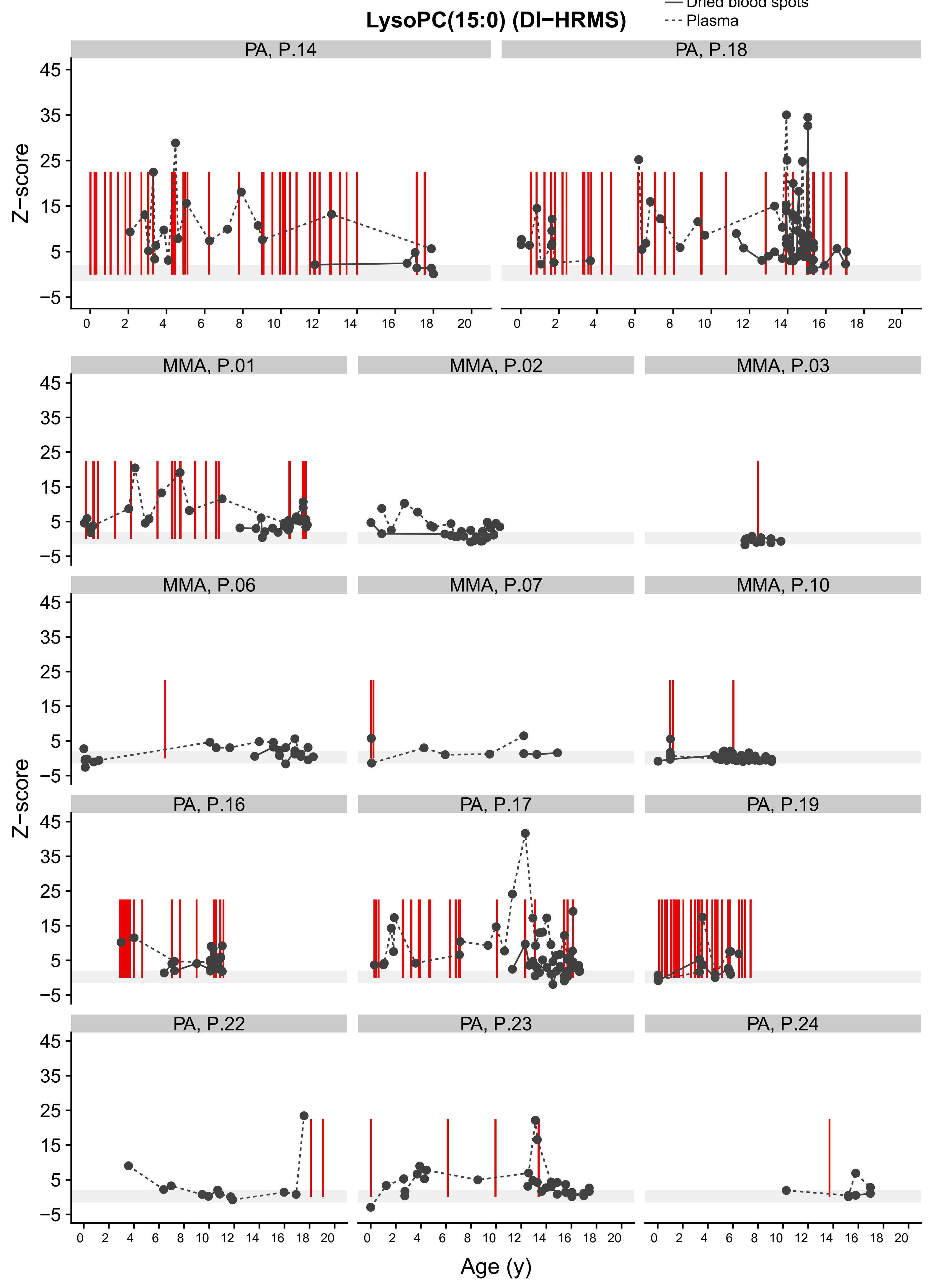

Supplementary Figure 36

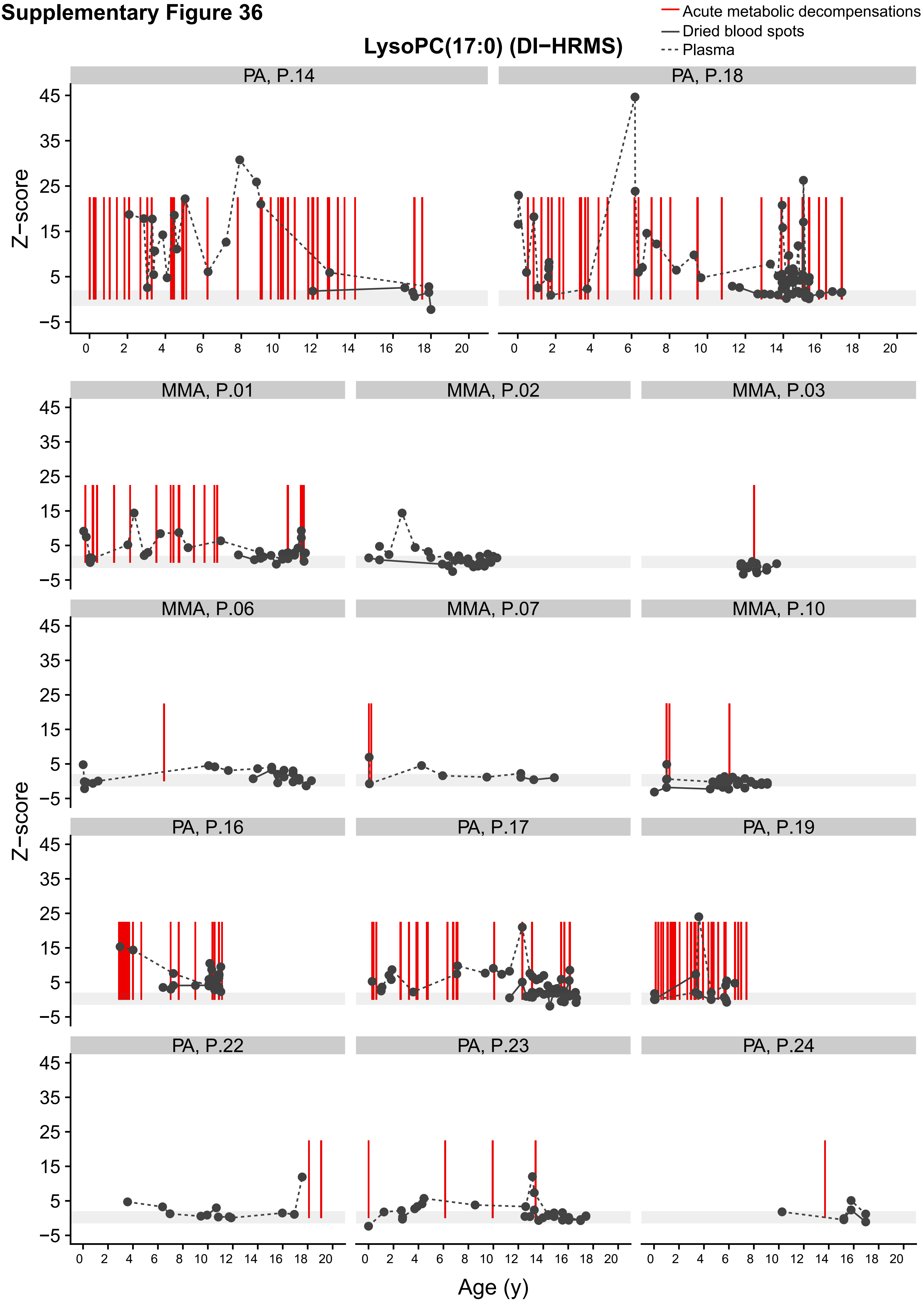

Supplementary Figure 37

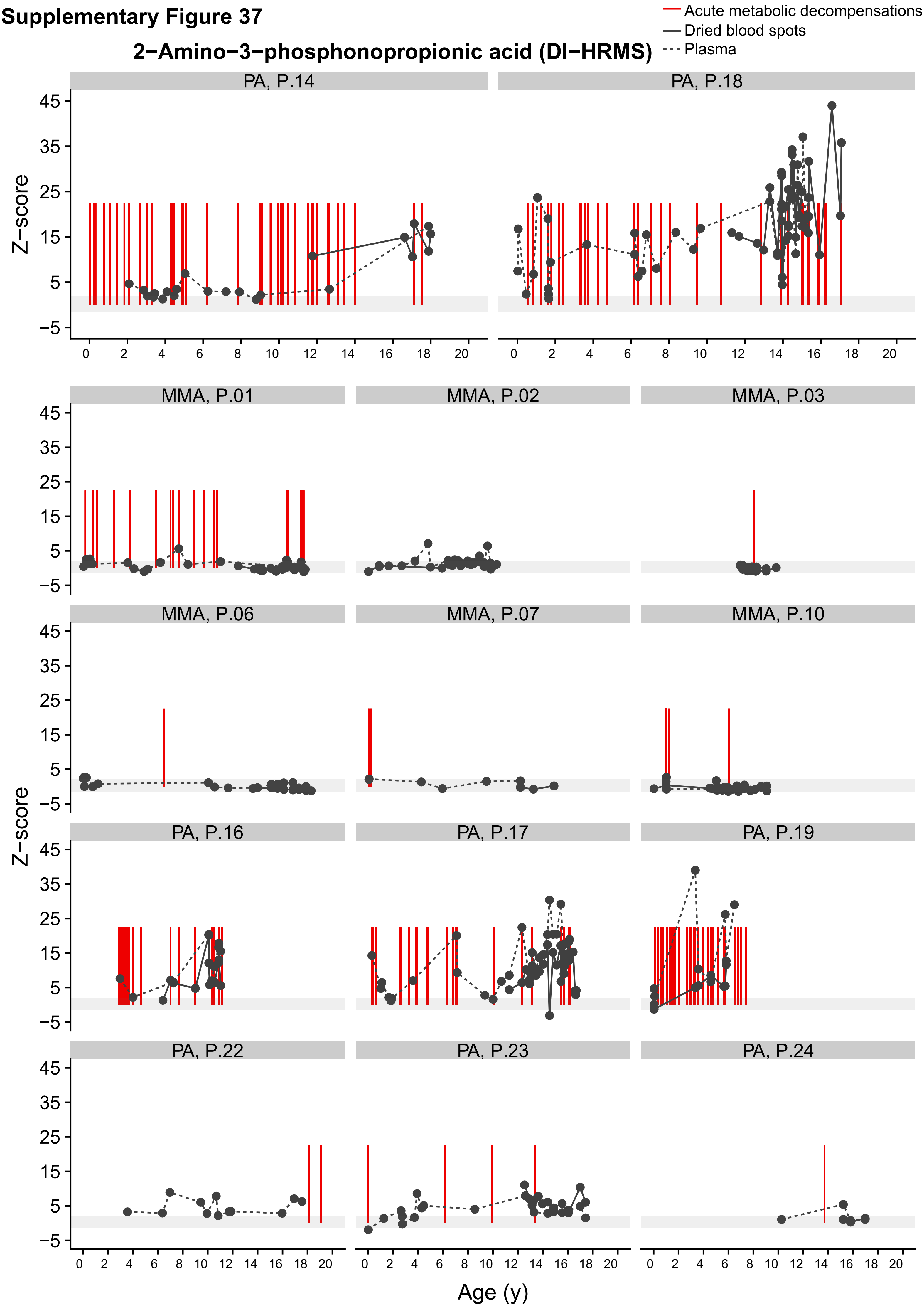

Supplementary Figure 38

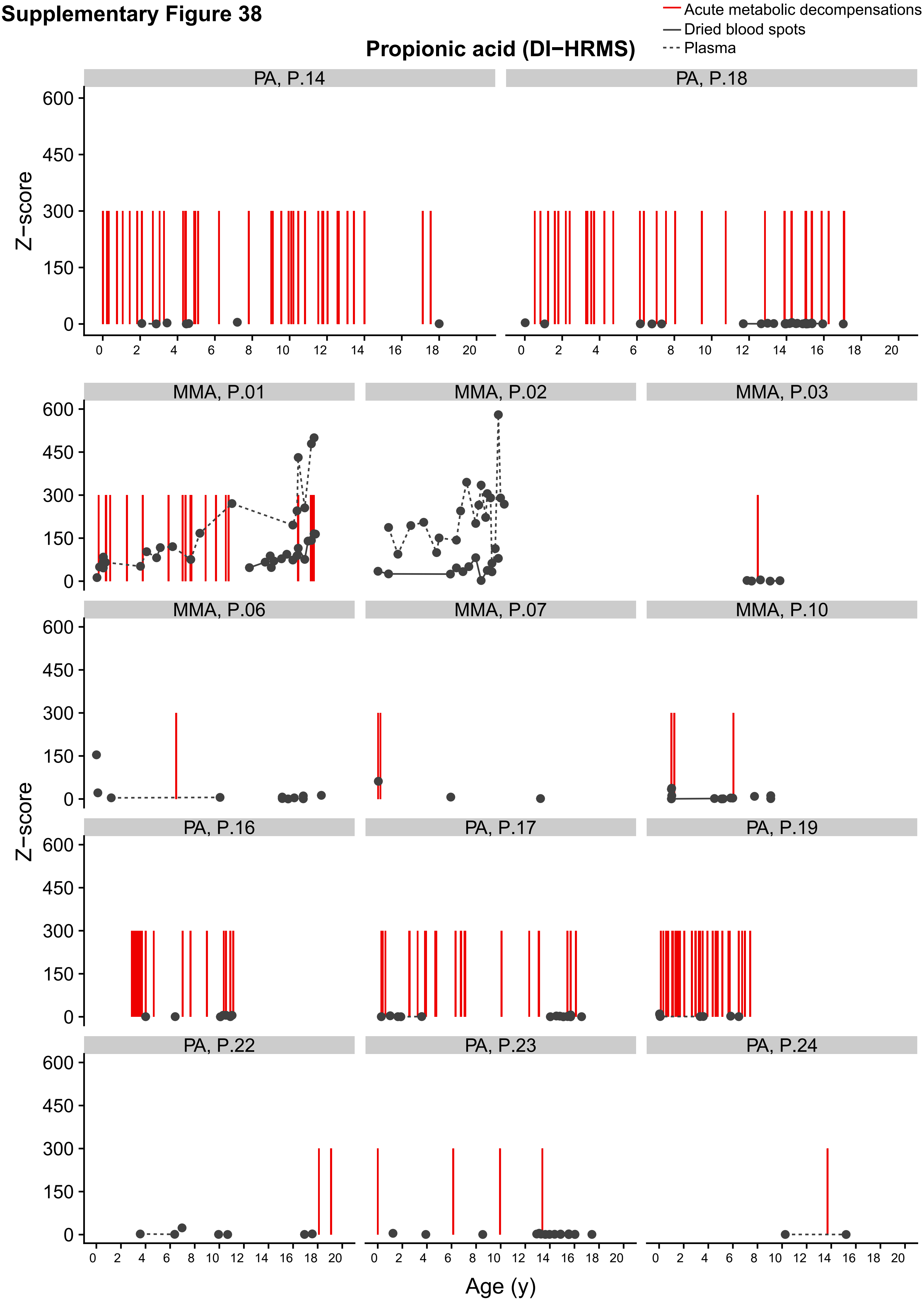

Supplementary Figure 39

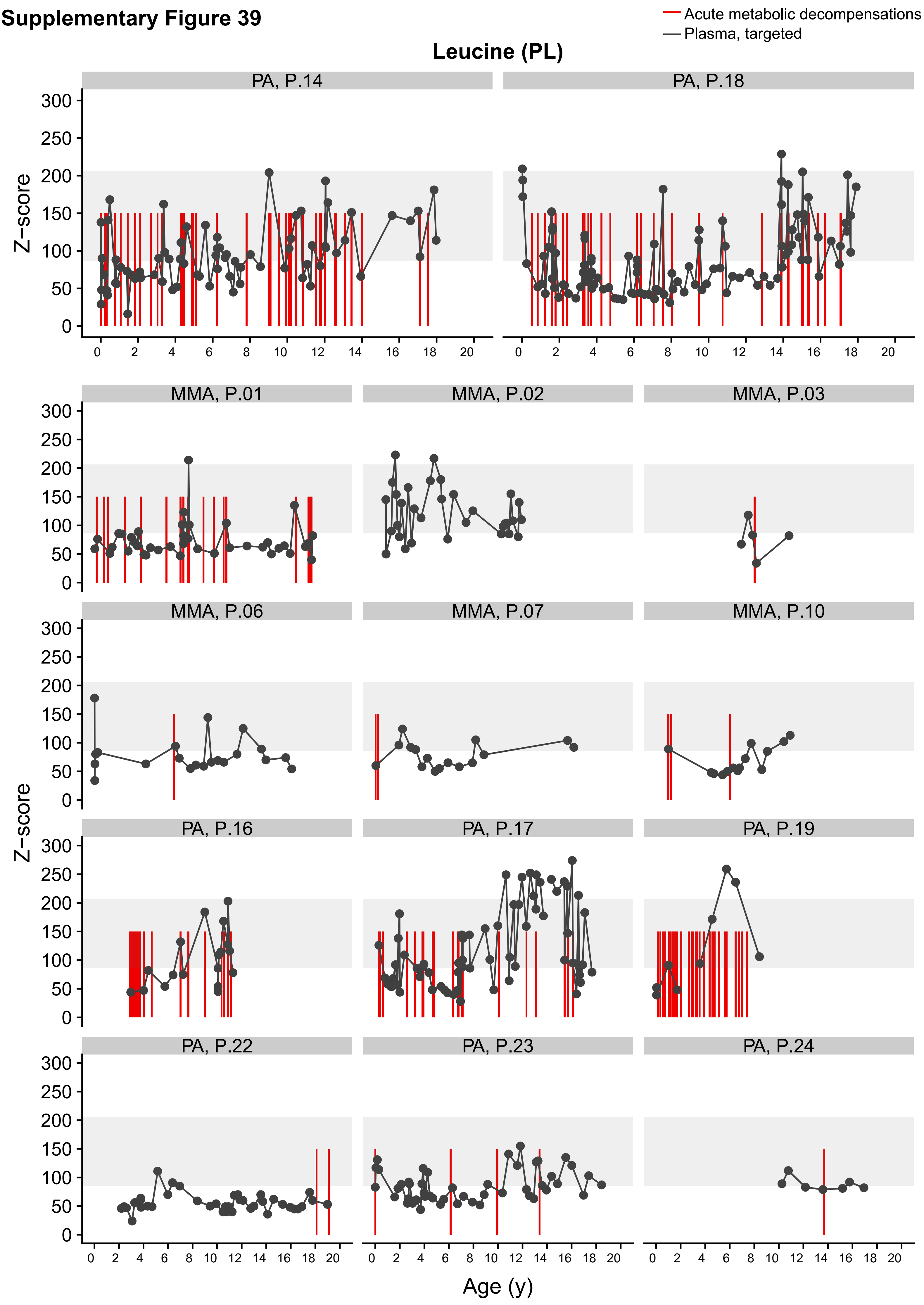

Supplementary Figure 40

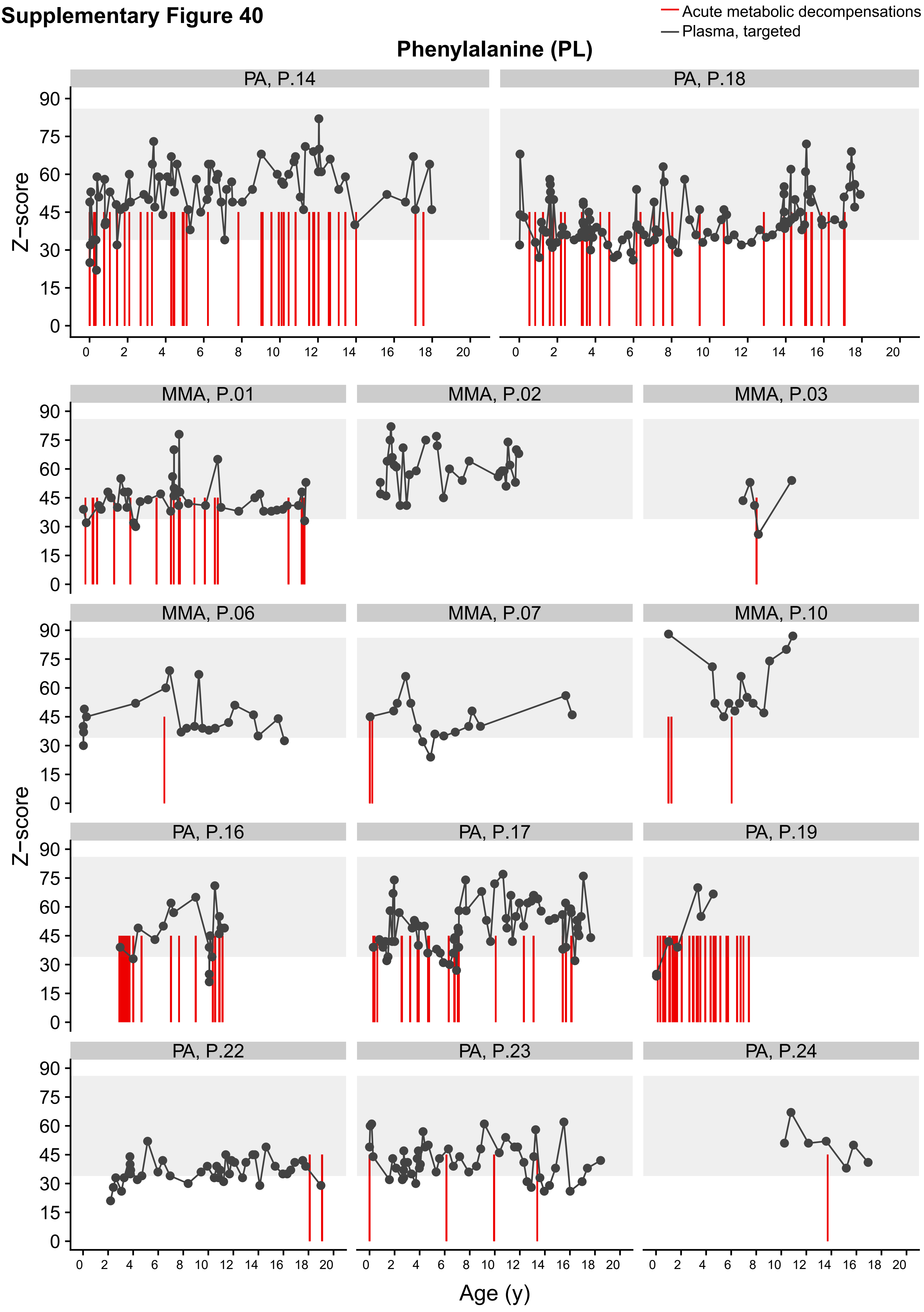

Supplementary Figure 41

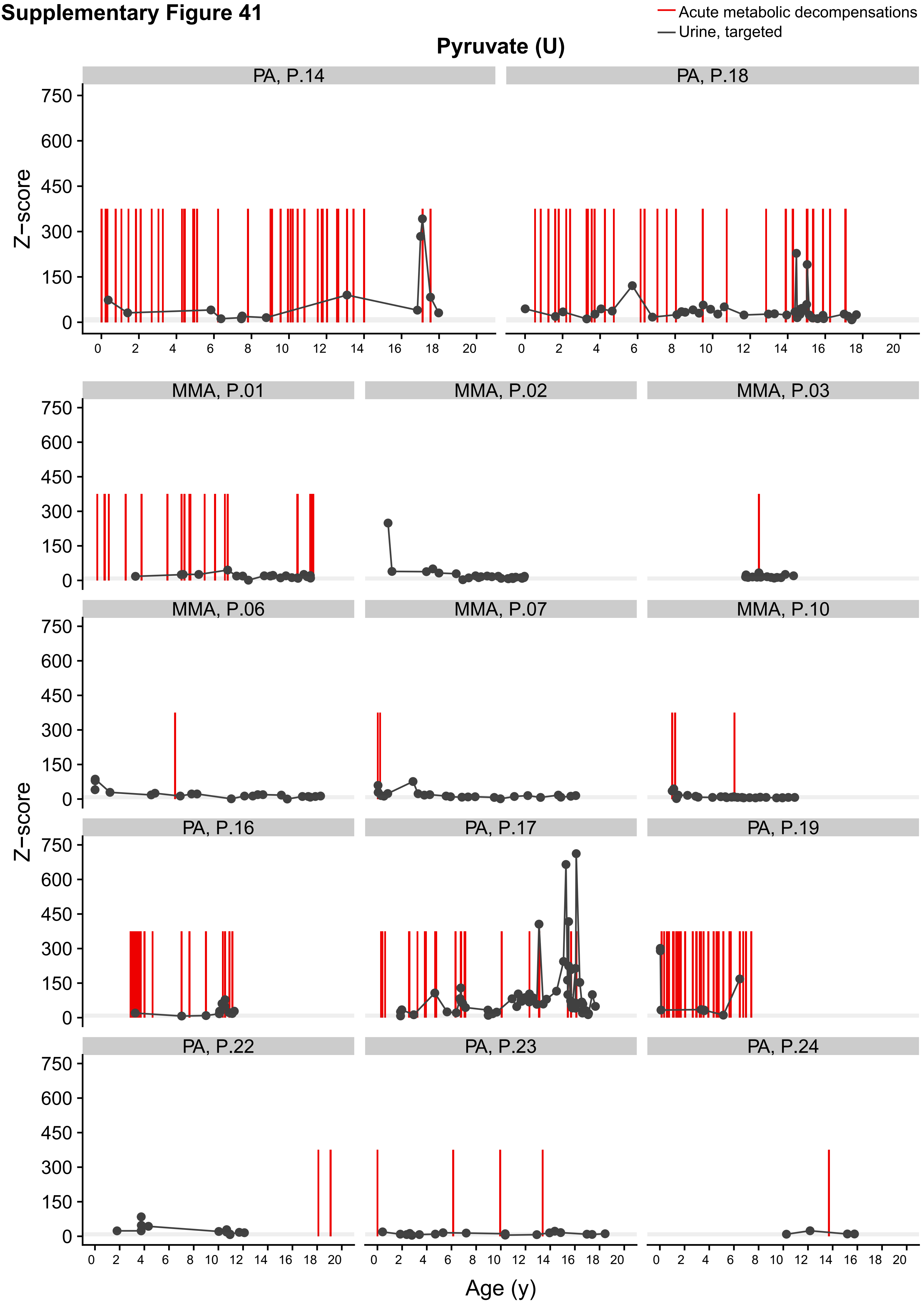

Supplementary Figure 42

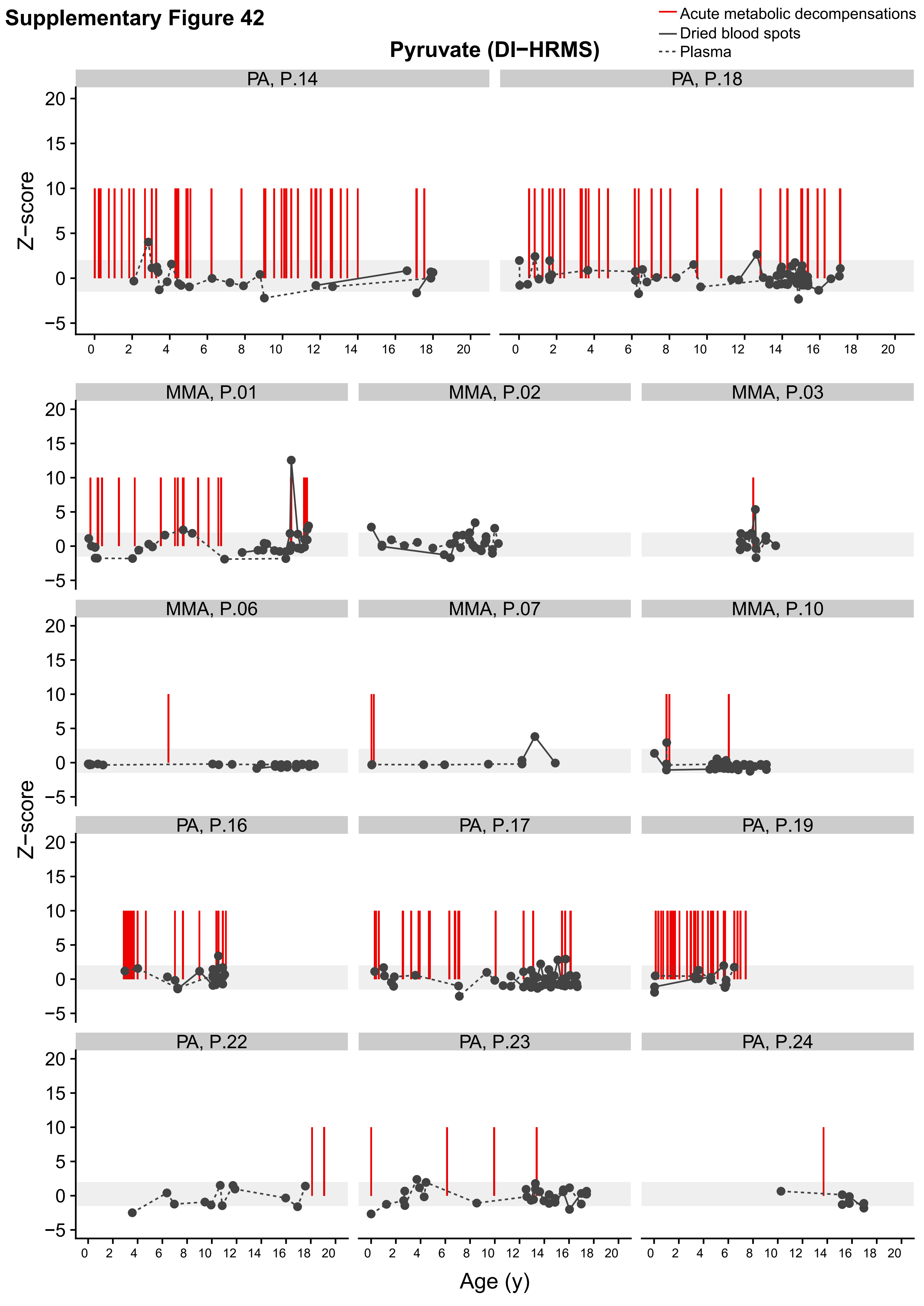

Supplementary Figure 43

PLS-DA, VIP-scores and heatmap for AMD samples versus no AMD samples, for PA and MMA

A) DBS

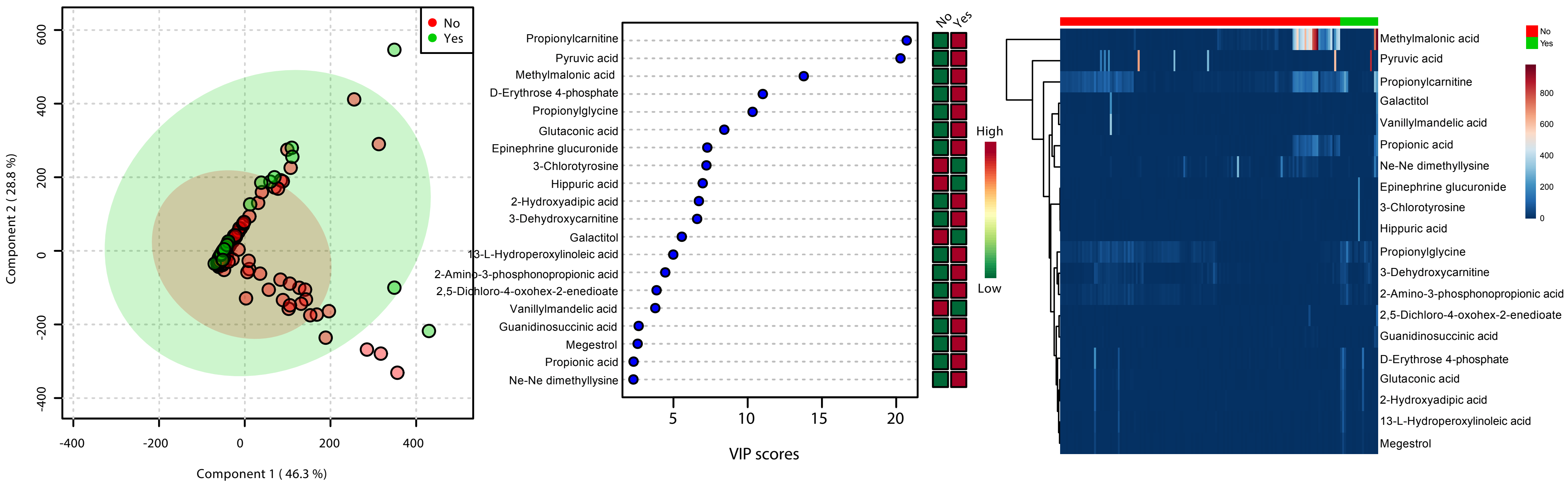

B) Plasma

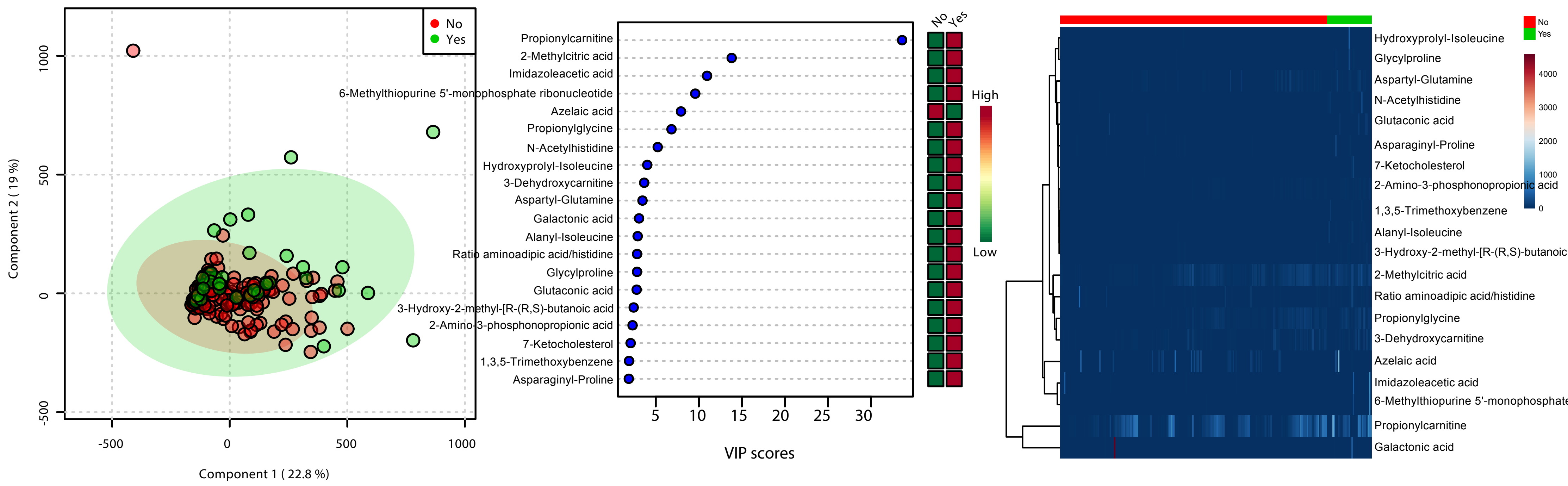

Supplementary Figure 44

PLS-DA, VIP-scores and heatmap for AMD samples versus no AMD samples, for PA only

A) DBS

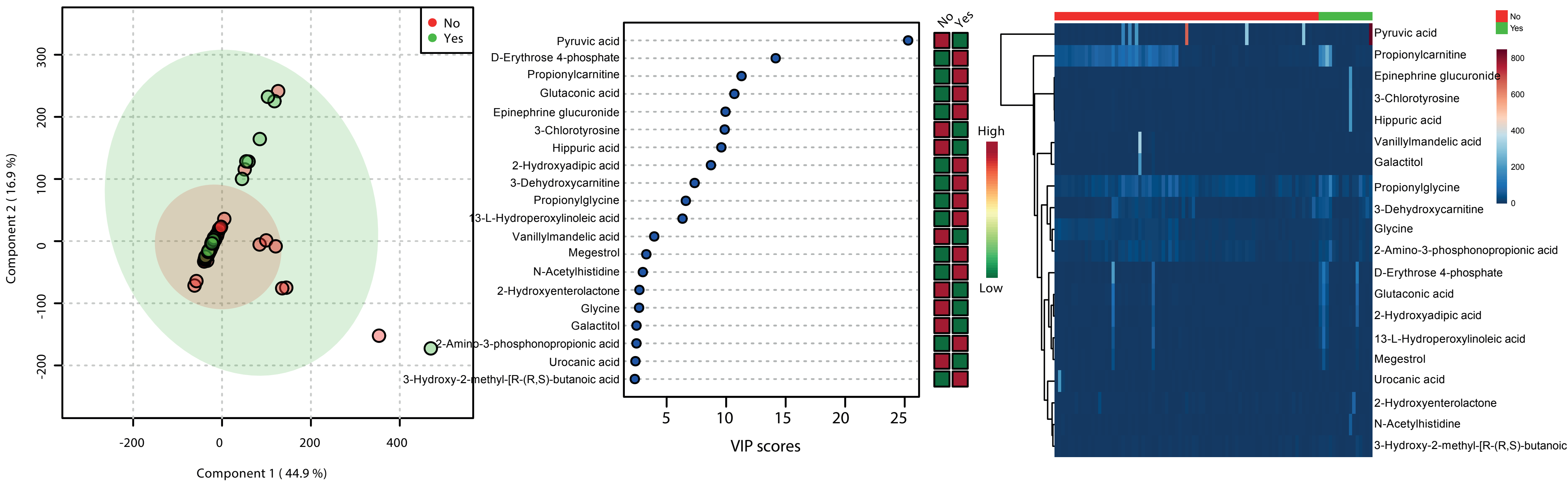

B) Plasma

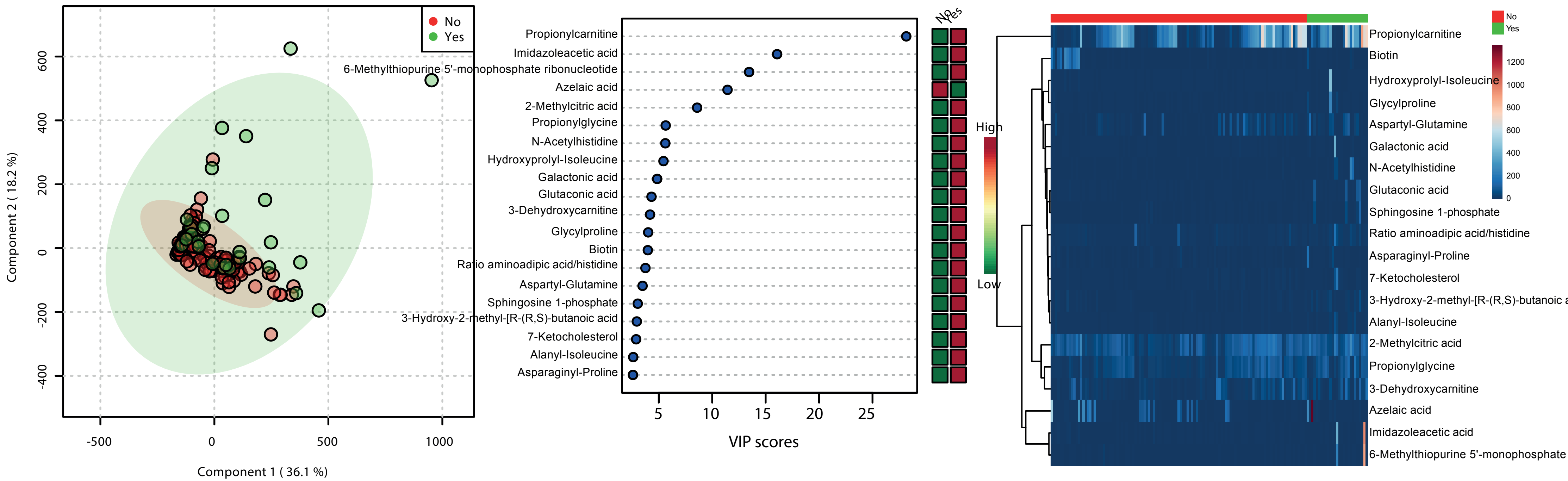

Supplement: Supplementary file 1 — Additional file 1. [file 13023_2020_1347_MOESM1_ESM.pdf]
