# Supplementary material for: Understanding acute metabolic decompensation in propionic and methylmalonic acidemias: a deep metabolic phenotyping approach
Source: Orphanet J Rare Dis. 2020 Mar 6;15:68. doi: 10.1186/s13023-020-1347-3 (PMC7060614; doi:10.1186/s13023-020-1347-3)
Supplement: Supplementary file 2 — Additional file 2. [file 13023_2020_1347_MOESM2_ESM.docx]

*Table S1, Reported biochemical parameters associated with PA or MMA, with the presence of AMD and with plasma ammonia in PA and MMA*

|  |  | **PROPIONIC ACIDURIA** | | **METHYLMALONIC ACIDURIA** | |
| --- | --- | --- | --- | --- | --- |
| **Analyte** | **Matrix** | **Alteration** | **References** | **Alteration** | **References** |
| *General disease biomarkers* |  |  |  |  |  |
| Glycine | Plasma, urine | Increase | Childs et al. 1961, Gompertz et al. 1970 | Increase | Rosenberg, Lilljeqvist and Hsia 1968 |
| Propionic acid | Plasma | Increase | Hommes et al. 1968, Ando et al. 1971 | Increase | Ando et al. 1971 |
| 2-Methylcitric acid | Urine | Increase | Ando et al 1972a, Chalmers et al. 1974 | Increase | Chalmers et al. 1974 |
| 3-Hydroxypropionic acid | Urine | Increase | Ando et al. 1972b | Increase | Ando et al. 1972b |
| Propionylglycine | Urine | Increase | Rasmussen et al. 1972a | Increase | Thompson and Chalmers 1990 |
| Tiglylglycine | Urine | Increase | Rasmussen et al. 1972b | Increase | Imen et al. 2012 |
| Tiglylcarnitine (C5:1-carn.) | Plasma, urine | Increase | Duran et al. 1982, Wikoff et al. 2007 | Increase | Duran et al. 1982, Wikoff et al. 2007 |
| Propionylcarnitine (C3-carn.) | Plasma | Increase | Roe et al. 1983 | Increase | Roe et al. 1983 |
| Heptadecanoylcarnitine | DBS | Increase | Malvagia et al. 2015 | Increase | Malvagia et al. 2015 |
| γ-butyrobetaine (C0-carn.) | Plasma | Increase | Wikoff et al. 2007 | Increase | Wikoff et al. 2007 |
| Acetylcarnitine (C2-carn.) | Plasma | Increase | Wikoff et al. 2007 | Increase | Wikoff et al. 2007 |
| Hexanoylcarnitine (C6-carn.) | Plasma | Increase | Wikoff et al. 2007 | Increase | Wikoff et al. 2007 |
| 2-Hexenoylcarnitine (C6:1-carn.) | Plasma | Increase | Wikoff et al. 2007 | Increase | Wikoff et al. 2007 |
| **3-Hydroxytetradecanoylcarnitine (C14-OH**-carn.**)** | Plasma | Increase | Wikoff et al. 2007 | Increase | Wikoff et al. 2007 |
| Tiglic acid | Urine | Increase | Nyhan et al. 1972 |  |  |
| Methylmalonic acid | Plasma, urine |  |  | Increase | Oberholzer et al. 1967, van den Berg, Boelkens and Hommes 1976 |
| Methylmalonylcarnitine (C4-DC-carn.) | Plasma, urine |  |  | Increase | Maeda et al. 2007 |
| Isovalerylcarnitine (C5-carn.) | Plasma |  |  | Increase | Wikoff et al. 2007 |
|  |  |  |  |  |  |
| *Biochemical parameters altered during AMD* |  |  |  |  |  |
| Ammonia | Plasma | Increase | Zwickler et al. 2014 | Increase | Zwickler et al. 2012 |
| Isoleucine | Plasma | Increase | Zwickler et al. 2014 | Increase | Zwickler et al. 2012 |
| Valine | Plasma | Increase | Zwickler et al. 2014 | Increase | Zwickler et al. 2012 |
| 3-Hydroxyisovaleric acid | Urine | Increase | Kuhara et al. 2012 | Increase | Kølvraa et al. 1980 |
| Lactic acid | Urine | Increase | Kuhara et al. 2012 | Increase | Kølvraa et al. 1980 |
| pH | Arterial blood gas | Decrease | Zwickler et al. 2014 | Decrease | Zwickler et al. 2012 |
| pCO_2_ | Arterial blood gas | Decrease | Zwickler et al. 2014 | Decrease | Zwickler et al. 2012 |
| Bicarbonate | Plasma | Decrease | Zwickler et al. 2014 | Decrease | Zwickler et al. 2012 |
| Base excess | Plasma | Decrease | Zwickler et al. 2014 | Decrease | Zwickler et al. 2012 |
| Glutamine | Plasma | Decrease | Zwickler et al. 2014 | Decrease | Zwickler et al. 2012 |
| Lactate | Plasma | Increase | Zwickler et al. 2014 |  |  |
| Anion gap | Plasma | Increase | Zwickler et al. 2014 |  |  |
| Glucose | Plasma | Increase | Zwickler et al. 2014 |  |  |
| Alanine | Plasma | Decrease | Zwickler et al. 2014 |  |  |
| Citrulline | Plasma | Decrease | Zwickler et al. 2014 |  |  |
| Glutamine + glutamate | Plasma | Decrease | Zwickler et al. 2014 |  |  |
| Free carnitine | Plasma | Decrease | Zwickler et al. 2014 |  |  |
| 3-Methylglutaconic acid | Urine | Increase | Kuhara et al. 2012 |  |  |
| Glutaric acid | Urine | Increase | Kuhara et al. 2012 |  |  |
| Ketone bodies  (3-hydroxybutyric acid and/or acetoacetate) | Urine | Increase | Zwickler et al. 2014 |  |  |
| Urea | Plasma |  |  | Increase | Zwickler et al. 2012 |
| Uric acid | Plasma |  |  | Increase | Zwickler et al. 2012 |
| Alanine aminotransferase (ALT) | Plasma |  |  | Increase | Zwickler et al. 2012 |
| 2-Methylcitric acid | Urine |  |  | Increase | Zwickler et al. 2012 |
| 3-Hydroxypropionic acid | Urine |  |  | Increase | Kølvraa et al. 1980 |
| Isovaleric acid | Urine |  |  | Increase | Kølvraa et al. 1980 |
| 2-Methylbutyric acid | Urine |  |  | Increase | Kølvraa et al. 1980 |
| Isobutyric acid | Urine |  |  | Increase | Kølvraa et al. 1980 |
| 2-Oxo acids | Urine |  |  | Increase | Kølvraa et al. 1980 |
| 3-Hydroxybutyric acid | Urine |  |  | Increase | Kølvraa et al. 1980 |
| 3-Hydroxyisobutyric acid | Urine |  |  | Increase | Kølvraa et al. 1980 |
|  |  |  |  |  |  |
| *Biochemical parameters correlated to plasma ammonia* |  |  |  |  |  |
| 2-Methylcitric acid | Urine  Plasma | Positive | Filipowicz et al. 2006  de Sain et al. 2014 |  |  |
| Propionylglycine | Urine | Positive | Filipowicz et al. 2006 |  |  |
| 3-Hydroxypropionic acid | Urine | Positive | Filipowicz et al. 2006 |  |  |
| Free carnitine | Urine | Positive | Filipowicz et al. 2006 |  |  |

The table lists, to the best of our knowledge, the first reports of general disease biomarkers for PA and MMA, of biochemical parameters associated with presence of AMD in PA and MMA and of biochemical parameters that correlate with plasma ammonia in PA and MMA. Analytes can have been reported also in other body fluids, and can have been reported also in other studies than the ones referred to.

*Table S2, diagnostic biomarkers for PA and isolated MMA*

|  |  | **PROPIONIC ACIDURIA** | | | | | | | **METHYLMALONIC ACIDURIA** | | | | | | |  |
| --- | --- | --- | --- | --- | --- | --- | --- | --- | --- | --- | --- | --- | --- | --- | --- | --- |
| **Analyte** | **Matrix** | **N** | **Median** | **SD** | **Min** | **Max** | **Ref. range** | **Known?** | **N** | **Median** | **SD** | **Min** | **Max** | **Ref. range** | **Known?** | |
| *Targeted metabolic assays* |  |  |  |  |  |  |  |  |  |  |  |  |  |  |  | |
| Propionylcarnitine | Plasma | 34 | 54.4 | 19.6 | 23.0 | 99.5 | 0.00-0.81 | Yes | 68 | 21.0 | 21.7 | 2.7 | 83.7 | 0.00-0.81 | Yes | |
| Glycine | Plasma | 73 | 1381 | 362 | 350 | 1962 | 166-330 | Yes | 77 | 492 | 369 | 168 | 1916 | 166-330 | Yes | |
| Isoleucine | Plasma | 73 | 21 | 9 | 7 | 48 | 34-106 | Yes, TH | 77 | 32 | 15 | 7 | 84 | 34-106 | Yes, TH | |
| Valine | Plasma | 73 | 66 | 26 | 20 | 183 | 155-343 | Yes, TH | 77 | 107 | 42 | 41 | 217 | 155-343 | Yes, TH | |
| Leucine | Plasma | 73 | 59 | 23 | 31 | 152 | 86-206 | Yes, TH | 77 | 77 | 39 | 34 | 223 | 86-206 | Yes, TH | |
| Threonine | Plasma | 73 | 72 | 29 | 29 | 212 | 102-246 | Yes, TH | 77 | 80 | 31 | 36 | 174 | 102-246 | Yes, TH | |
| Acetylcarnitine | Plasma | 34 | 10.1 | 7.5 | 1.6 | 42.2 | 0.69-9.72 | Yes | 68 | 9.2 | 5.8 | 2.1 | 29.9 | 0.69-9.72 |  | |
| Methylcitric acid | Urine | 3 | 697 | 383 | 426 | 968 |  | Yes | 0 |  |  |  |  |  |  | |
| 3-Hydroxy-propionic acid | Urine | 10 | 232 | 99 | 89 | 349 | 0-20 | Yes | 57 | 13 | 19 | 0 | 86 | 0-20 |  | |
| Histidine | Plasma | 73 | 65 | 14 | 32 | 107 | 68-108 | No | 77 | 72 | 15 | 45 | 142 | 68-108 |  | |
| Methylmalonic acid | Urine | 12 | 2.0 | 3.1 | 1.0 | 10.0 | 0-20 |  | 174 | 727 | 4651 | 64 | 21587 | 0-20 | Yes | |
| Methylmalonic acid | Plasma | 0 |  |  |  |  | 0.12-0.29 |  | 52 | 73.8 | 345.8 | 2.9 | 1327.4 | 0.12-0.29 | Yes | |
| Methylmalonylcarnitine | Plasma | 26 | 0.01 | 0.01 | 0.01 | 0.04 | 0.00-0.07 |  | 68 | 0.34 | 0.52 | 0.10 | 2.69 | 0.00-0.07 | Yes | |
| Methylcitric acid | Plasma | 0 |  |  |  |  | 0.00-0.83 |  | 11 | 2.2 | 2.8 | 1.0 | 10.9 | 0.00-0.83 | Yes | |
| Glutamine | Plasma | 73 | 494 | 105 | 252 | 793 | 457-857 |  | 77 | 380 | 126 | 190 | 736 | 457-857 | No | |
|  |  |  |  |  |  |  |  |  |  |  |  |  |  |  |  | |
| *Untargeted DI-HRMS analysis* |  |  |  |  |  |  | **P-value** |  |  |  |  |  |  | **P-value** |  | |
| 2-Methylcitric acid ( 3 isomers) | Plasma | 23 | 57.4 | 43.1 | -1.8 | 146.9 | <0.0001 | Yes | 51 | 6.1 | 7.6 | -0.6 | 25.5 | <0.0001 | Yes | |
| 3-Dehydroxycarnitine | Plasma | 23 | 15.9 | 53.2 | 0.4 | 203.4 | <0.0001 | Yes | 51 | 3.2 | 7.0 | -0.6 | 26.6 | <0.0001 | Yes | |
| Propionylcarnitine | Plasma | 23 | 12.0 | 80.8 | -2.8 | 362.6 | <0.0001 | Yes | 51 | 27.9 | 80.0 | -1.4 | 368.0 | <0.0001 | Yes | |
| Fructoseglycine | Plasma | 23 | 7.6 | 5.7 | -0.8 | 25.8 | <0.0001 | No | 51 | 2.2 | 2.2 | -1.2 | 7.6 | <0.0001 | No | |
| Isoleucyl-Isoleucine (3 isomers) | Plasma | 23 | 5.7 | 3.8 | -0.6 | 13.9 | <0.0001 | No | 51 | 3.4 | 2.8 | -1.0 | 10.8 | <0.0001 | No | |
| LysoPC(15:0) (2 isomers) | Plasma | 23 | 5.5 | 4.6 | -2.9 | 16.0 | <0.0001 | No | 51 | 2.5 | 4.1 | -1.7 | 20.5 | <0.0001 | No | |
| Glucosamine (2 isomers) | Plasma | 23 | 5.1 | 8.9 | -2.5 | 31.1 | <0.0001 | No | 51 | 2.2 | 4.8 | -0.7 | 22.2 | <0.0001 | No | |
| Threonic acid | Plasma | 23 | 2.1 | 1.6 | -2.8 | 3.6 | 0.0009 | No | 51 | 2.5 | 2.4 | -1.4 | 8.0 | <0.0001 | No | |
| DL-2-Aminooctanoic acid | Plasma | 23 | -1.6 | 0.1 | -1.7 | -1.3 | <0.0001 | No | 51 | -1.5 | 0.5 | -1.7 | 0.4 | <0.0001 | No | |
| 2-Methyl-3-ketovaleric acid (7 isomers) | Plasma | 23 | -1.8 | 0.3 | -2.8 | -1.3 | <0.0001 | No | 51 | -2.1 | 0.9 | -2.8 | 1.9 | <0.0001 | No | |
| L-Glutamine (4 isomers) | Plasma | 23 | -1.8 | 0.1 | -1.9 | -1.6 | <0.0001 | No | 51 | -1.5 | 0.9 | -2.1 | 1.5 | <0.0001 | No | |
| L-Methionine | Plasma | 23 | -2.0 | 05 | -2.5 | -0.5 | <0.0001 | No | 51 | -1.5 | 0.5 | -2.1 | 0.0 | <0.0001 | No | |
| L-Histidine | Plasma | 23 | -2.8 | 1.4 | -5.6 | -0.6 | <0.0001 | No | 51 | -1.7 | 1.3 | -5.2 | 1.6 | <0.0001 | No | |
| Propionylglycine (9 isomers) | Plasma | 23 | 28.5 | 29.3 | -3.0 | 91.1 | <0.0001 | Yes | 51 | 1.1 | 2.4 | -2.4 | 8.7 |  |  | |
| Glycine | Plasma | 23 | 9.5 | 4.8 | -2.4 | 18.5 | <0.0001 | Yes | 51 | 1.5 | 2.2 | -1.1 | 8.5 |  |  | |
| 2-Amino-3-phosphonopropionic acid | Plasma | 23 | 6.2 | 6.5 | -1.9 | 23.7 | <0.0001 | No | 51 | 0.8 | 1.3 | -1.3 | 7.1 |  |  | |
| 1-(sn-Glycero-3-phospho)-1D-myo-inositol | Plasma | 23 | 5.3 | 3.9 | -0.6 | 10.8 | <0.0001 | No | 51 | 0.3 | 4.8 | -2.7 | 25.5 |  |  | |
| LysoPC(17:0) (2 isomers) | Plasma | 23 | 4.1 | 5.3 | -2.3 | 17.3 | <0.0001 | No | 51 | 1.5 | 3.4 | -2.5 | 14.4 |  |  | |
| Homocysteine | Plasma | 23 | -1.7 | 1.2 | -3.5 | 2.1 | 0.0002 | No | 51 | -0.9 | 1.2 | -3.0 | 4.1 |  |  | |
| L-Isoleucine (6 isomers) | Plasma | 23 | -1.7 | 0.7 | -2.8 | 0.1 | <0.0001 | Yes | 51 | -1.3 | 0.9 | -2.8 | 2.6 |  |  | |
| Methylmalonic acid (3 isomers) | Plasma | 23 | 0.1 | 1.7 | -2.2 | 7.0 |  |  | 51 | 10.0 | 50.7 | 0.0 | 228.6 | <0.0001 | Yes | |
| Propionic acid (2 isomers) | Plasma | 23 | -0.3 | 4.4 | -2.4 | 20.1 |  |  | 51 | 6.9 | 79.1 | -1.1 | 344.9 | <0.0001 | No | |
| 3-Hydroxy-9-hexadecenoylcarnitine | Plasma | 23 | 3.1 | 3.1 | -1.7 | 8.8 |  |  | 51 | 3.6 | 7.6 | -0.9 | 40.0 | <0.0001 | No | |

N: number of samples. SD: standard deviation. Min: minimum value. Max: maximum value. Ref. range: reference range. TH: therapy related. DI-HRMS: direct-infusion high-resolution mass spectrometry. Results of targeted metabolic assays in plasma are presented in µmol/L, results of targeted metabolic assays in urine are presented in mmol/mol creatinine. All p-values were adjusted according to the Bonferroni method. A p-value <0.05 was considered statistically significant.
